# Interventions to reengage people living with HIV who are lost to follow-up from HIV treatment programs: A systematic review and meta-analysis

**DOI:** 10.1371/journal.pmed.1003940

**Published:** 2022-03-15

**Authors:** Ali Mirzazadeh, Ingrid Eshun-Wilson, Ryan R. Thompson, Atousa Bonyani, James G. Kahn, Stefan D. Baral, Sheree Schwartz, George Rutherford, Elvin H. Geng

**Affiliations:** 1 Division of Infectious Disease and Global Epidemiology, Department of Epidemiology and Biostatistics, and Institute for Global Health Sciences, University of California San Francisco, San Francisco, California, United States of America; 2 Division of Infectious Diseases, School of Medicine, Washington University at St Louis, St Louis, Missouri, United States of America; 3 Francis I. Proctor Foundation, University of California San Francisco, San Francisco, California, United States of America; 4 World Health Organization, Tehran, Iran; 5 University of California San Francisco, San Francisco, California, United States of America; 6 Department of Epidemiology, Johns Hopkins Bloomberg School of Public Health, Baltimore, Maryland, United States of America; 7 Center for Dissemination and Implementation, Institute for Public Health, Washington University in St. Louis, St Louis, Missouri, United States of America; University of Southampton, UNITED KINGDOM

## Abstract

**Background:**

Optimizing services to facilitate engagement and retention in care of people living with HIV (PLWH) on antiretroviral therapies (ARTs) is critical to decrease HIV-related morbidity and mortality and HIV transmission. We systematically reviewed the literature for the effectiveness of implementation strategies to reestablish and subsequently retain clinical contact, improve viral load suppression, and reduce mortality among patients who had been lost to follow-up (LTFU) from HIV services.

**Methods and findings:**

We searched 7 databases (PubMed, Cochrane, ERIC, PsycINFO, EMBASE, Web of Science, and the WHO regional databases) and 3 conference abstract archives (CROI, IAC, and IAS) to find randomized trials and observational studies published through 13 April 2020. Eligible studies included those involving children and adults who were diagnosed with HIV, had initiated ART, and were subsequently lost to care and that reported at least one review outcome (return to care, retention, viral suppression, or mortality). Data were extracted by 2 reviewers, with discrepancies resolved by a third. We characterized reengagement strategies according to how, where, and by whom tracing was conducted. We explored effects, first, among all categorized as LTFU from the HIV program (reengagement program effect) and second among those found to be alive and out of care (reengagement contact outcome). We used random-effect models for meta-analysis and conducted subgroup analyses to explore heterogeneity. Searches yielded 4,244 titles, resulting in 37 included studies (6 randomized trials and 31 observational studies). In low- and middle-income countries (LMICs) (*N =* 16), tracing most frequently involved identification of LTFU from the electronic medical record (EMR) and paper records followed by a combination of telephone calls and field tracing (including home visits), by a team of outreach workers within 3 months of becoming LTFU (*N =* 7), with few incorporating additional strategies to support reengagement beyond contact (*N =* 2). In high-income countries (HICs) (*N* = 21 studies), LTFU were similarly identified through EMR systems, at times matched with other public health records (*N =* 4), followed by telephone calls and letters sent by mail or email and conducted by outreach specialist teams. Home visits were less common (*N =* 7) than in LMICs, and additional reengagement support was similarly infrequent (*N* = 5). Overall, reengagement programs were able to return 39% (95% CI: 31% to 47%) of all patients who were characterized as LTFU (*n =* 29). Reengagement contact resulted in 58% (95% CI: 51% to 65%) return among those found to be alive and out of care (*N =* 17). In 9 studies that had a control condition, the return was higher among those in the reengagement intervention group than the standard of care group (RR: 1.20 (95% CI: 1.08 to 1.32, *P* < 0.001). There were insufficient data to generate pooled estimates of retention, viral suppression, or mortality after the return.

**Conclusions:**

While the types of interventions are markedly heterogeneity, reengagement interventions increase return to care. HIV programs should consider investing in systems to better characterize LTFU to identify those who are alive and out of care, and further research on the optimum time to initiate reengagement efforts after missed visits and how to best support sustained reengagement could improve efficiency and effectiveness.

## Author summary

**Why was this study done?:**

- Sustained engagement and retention in HIV care is critical for optimal HIV treatment outcomes and reduced HIV transmission.
- For many people living with HIV, disengagement is inevitable during the long course of HIV treatment.
- It remains unclear which combination of reengagement strategies are most effective and under what conditions.

**What did the researchers do and find?:**

- Our systematic review identified 37 studies (6 randomized trials and 31 observational studies), 16 of which were conducted in low- and middle-income countries.
- The majority of randomized trials were assessed as low risk (*N =* 3) or of some concern (*N* = 3) regarding the methodological quality. Most cohort studies that had a control condition were also assessed as good quality (*N* = 7 out of 10).
- Based on our meta-analysis, reengagement programs were able to return 39% of all patients who were characterized as lost to follow-up, reengagement contact resulted in 58% return among those found to be alive and out of care.
- In 9 studies that had a control condition, the return was 20% higher among those in the reengagement intervention group than the standard of care.

**What do these findings mean?:**

- Despite marked heterogeneity of intervention characteristics, reengagement interventions may increase return to care.
- Further research on the optimum time to initiate reengagement efforts after missed visits and how to best support sustained reengagement could improve efficiency and effectiveness.

## Introduction

While sustained engagement and retention in HIV care are critical for optimal HIV treatment outcomes and reduced HIV transmission, for many people living with HIV (PLWH), disengagement is inevitable during the long course of HIV treatment. Reasons for disengagement are varied in both high- and low-income settings, and include health system, structural, and psychosocial barriers to care [1–7]. Many PLWH will return to care after a short gap without intervention, while others will remain out of care for longer periods, resulting in clinical deterioration, persistent viremia, and ongoing HIV transmission in the community [2,8,9]. Reengagement interventions have the potential to hasten return by improving access to care and assisting PLWH to overcome barriers to return.

Although outreach to those who are lost to follow-up (LTFU) forms part of many HIV program operations, it remains unclear which combination of reengagement strategies are most effective and under what conditions. Difficulties faced by reengagement programs include as a first step enumerating those who have truly disengaged (are alive and out of care) as opposed to those characterized as LTFU (which frequently includes PLWH who have died or transferred care). Programs are then faced with a number of strategies to incorporate depending on expertise and resources. Reengagement strategies most frequently include attempts to contact patients and encourage return, in some cases followed by specific strategies that support return such as provider or patient notification systems [10], peer or provider navigation [11], intensive case management and outreach follow-up [12], and transport support interventions. Understanding which of the reengagement strategies’ components are most effective and when the reengagement should be started and for whom could aid the development of future reengagement programs.

To characterize reengagement strategies and explore the effectiveness of reengagement programs on return to the care, we conducted a systematic review and meta-analysis of interventions to improve return to care among PLWH lost to HIV programs globally.

## Methods

Our search, screening, study selection, analysis, and methods were described and registered a priori in PROSPERO (PROSPERO 2019 # CRD42019130436).

### Inclusion criteria and outcome definitions

We included studies conducted in PLWH on antiretroviral therapy (ART) of any age and considered LTFU (unknown treatment outcomes) by HIV programs. Reengagement interventions included strategies aimed at identifying care status among those LTFU and encouraging return to care among those found alive and out of care. Interventions may have been directed toward patients (such as peer or provider outreach into the community and navigation) and toward providers and clinics (e.g., through reminders and alerts). We included observational studies with or without comparators, randomized controlled trials (RCTs), and non-RCTs. Comparative arms included standard of care (SOC) or other reengagement interventions.

**Fig 1:**
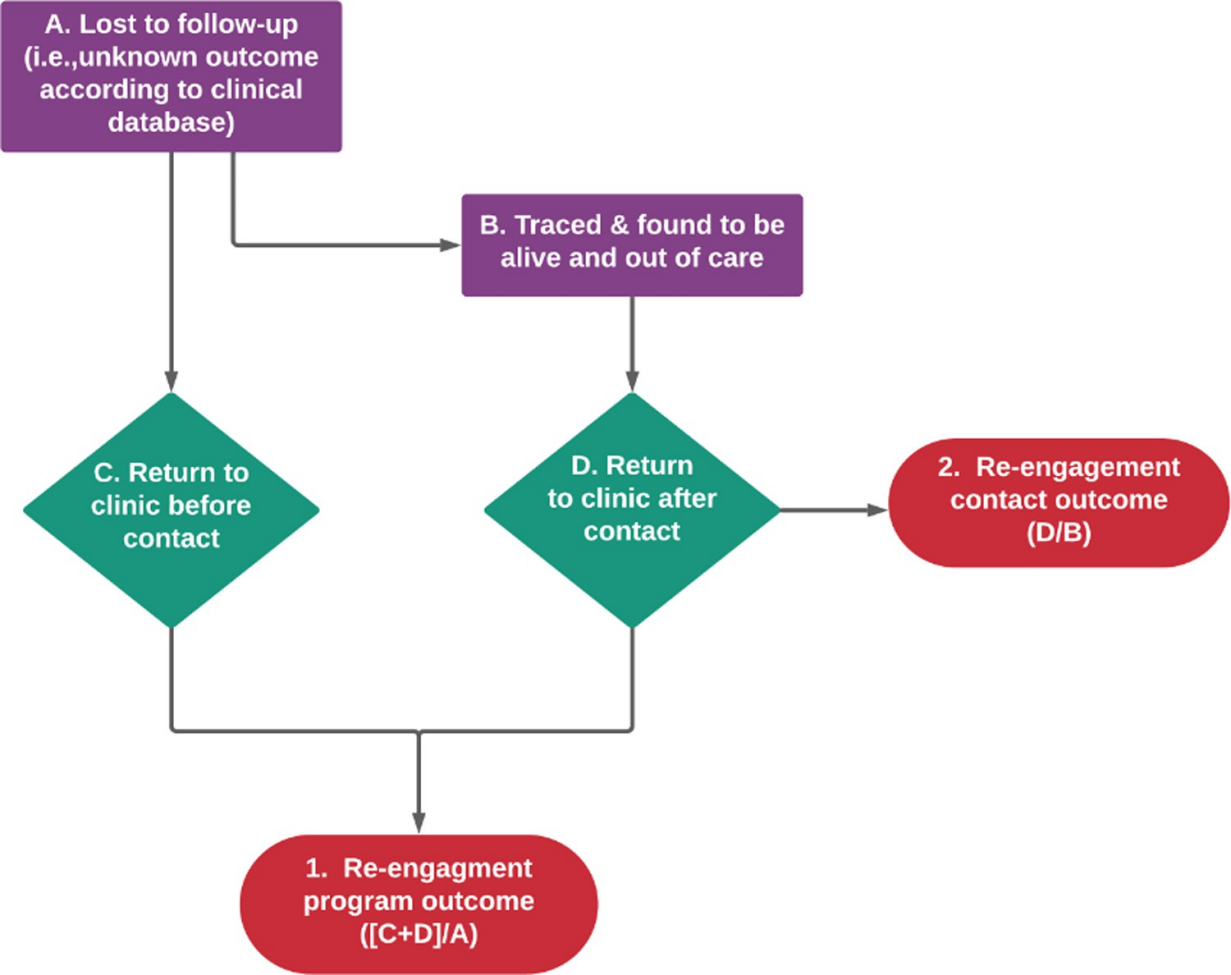
Flow diagram depicting reengagement outcomes: (1) reengagement program outcome; and (2) reengagement contact outcome.

We consider reengagement interventions to have 2 types of effects. First, the effect of the entire program of reengagement (including the filtering of records and tracing to identify true outcomes) as the effects of a “reengagement program” (**Fig 1**), which we broadly consider a measure of “effectiveness.” This is in part motivated by the fact that activities to ascertain outcomes often cannot be fully distinguished from activities to return patients (e.g., a telephone call to find the status of the patient could also influence return). In addition, the entire body of effort that goes into returning a patient must include identifying the patient who is out of care, and therefore efforts to classify is part of the programmatic reality of efforts to return patients. The second type of effects are those of actually contacting a patient who is out of care. We call this the effects of “reengagement contact,” which is analogous to “efficacy.” Additional study outcomes included retention on ART, viral suppression, and all-cause mortality following subsequent return at any time point as reported in the paper.

### Search strategy

We searched 7 databases, which included PubMed, the Cochrane Central Register of Controlled Trials, Education Resource Information Center (ERIC), PsycINFO, EMBASE, Web of Science, the WHO regional databases (using Global Index Medicus metasearch engine), and conference abstract archives on the websites of the Conference on Retroviruses and Opportunistic Infections (CROI), the International AIDS Conference (IAC), and the International AIDS Society Conference on HIV Science (since 2017). We also searched for ongoing trials in the National Institutes of Health’s trials registry at ClinicalTrials.gov and the WHO International Clinical Trial Registries Platform (ICTRP) and examined the bibliographies of included studies and other relevant references. Details of our search strategy are provided in **Appendix A in S1 Text.**

### Screening and data extraction

The abstract and full-text screening was done independently in Covidence [13] by 2 coauthors, and discrepancies were resolved by a third author. After confirming eligibility, a single author extracted data, verified by a second author. The following data were extracted from each included study: (1) location; (2) study design; (3) population (sample size, age, sex, proportion of key populations, inclusion/exclusion criteria); (4) intervention and comparator characteristics; and (5) outcomes: primary and secondary outcomes, extracted when possible with numerators, denominators, and/or measures of association (we extracted the number of all patients who were LTFU, traced and successfully contacted, died, moved out, transferred clinics, incarcerated, hospitalized, or lost for other reasons). Any discrepancies were resolved by discussion among the authors.

### Assessments of methodological quality, real-world relevance, and GRADE

We assessed the risk of bias according to the Cochrane Handbook [14] for RCTs or Newcastle–Ottawa Scale tool criteria [15] for observational studies. To further assess the generalizability of study findings, we used the PRECIS-2 checklist to assess how pragmatic or explanatory studies included in the comparative analyses were [16]. We applied the tool’s 9 domains (eligibility, recruitment/cohort selection, setting, organization, flexibility: delivery, flexibility: adherence, follow-up, primary outcome, primary analysis) to determine how applicable findings might be to real-world settings. We additionally evaluated the certainty of the body of evidence contributing to pooled effect estimates for comparative analyses using criteria recommended by the GRADE Working Group [17–21].

This study is reported as per the Preferred Reporting Items for Systematic Reviews and Meta-Analyses (PRISMA) guideline (**Appendix G in S1 Tex**t).

### Analysis

We calculated the proportion returned to care according to 2 outcome definitions (**Fig 1**). The “reengagement program outcome” was calculated as the number who returned to care at the original clinic out of all LTFU after the intervention was initiated, and the second definition was the number who returned to care at the original clinic after the intervention was initiated among those who were identified to be alive and out of care (disengaged). The proportions retained, virally suppressed, and died were calculated among those who returned to care. For each individual study, we calculated the proportion and the score (Wilson) confidence intervals [22] using the metaprop command in STATA (version 15.1). In studies that had a comparator (control condition), the adjusted risk ratio (RR) or risk difference (RD) point estimate and the lower and upper limits of the 95% confidence interval (CI) were extracted. We used random effect models based on the inverse variance method (metan command in STATA v. 15.1) to pool the proportions or effect measures. We conducted subgroup analyses by study design, country income, tracing type, time when tracing started, time when outcome measured, the definition of LTFU, number of tracing attempts, who traced the patient, and intervention subtype. We conducted subgroup analyses to explore heterogeneity in results; chi-squared tests for heterogeneity were used to check whether the true effect in all studies is the same. We also quantified the heterogeneity using the I-squared measure.

We assessed the publication bias visually using a funnel plot (the standard normal deviation of intervention effect estimates against its precision) [23] and by the regression-based Egger test for small-study effects [24]. The results are presented in **Appendix H in S1 Text**. Both funnel plot and Egger test indicated a publication bias for the reengagement program effects outcome (proportion LTFU returned to the original clinic; reengagement program versus no intervention or SOC). The funnel plot for this outcome is asymmetrical, which indicates possible publication bias. This may mean a bias toward more favorable results in the published literature since nonsignificant findings tend disproportionately not to be published [25].

## Results

### Characteristics of included studies

**Fig 2:**
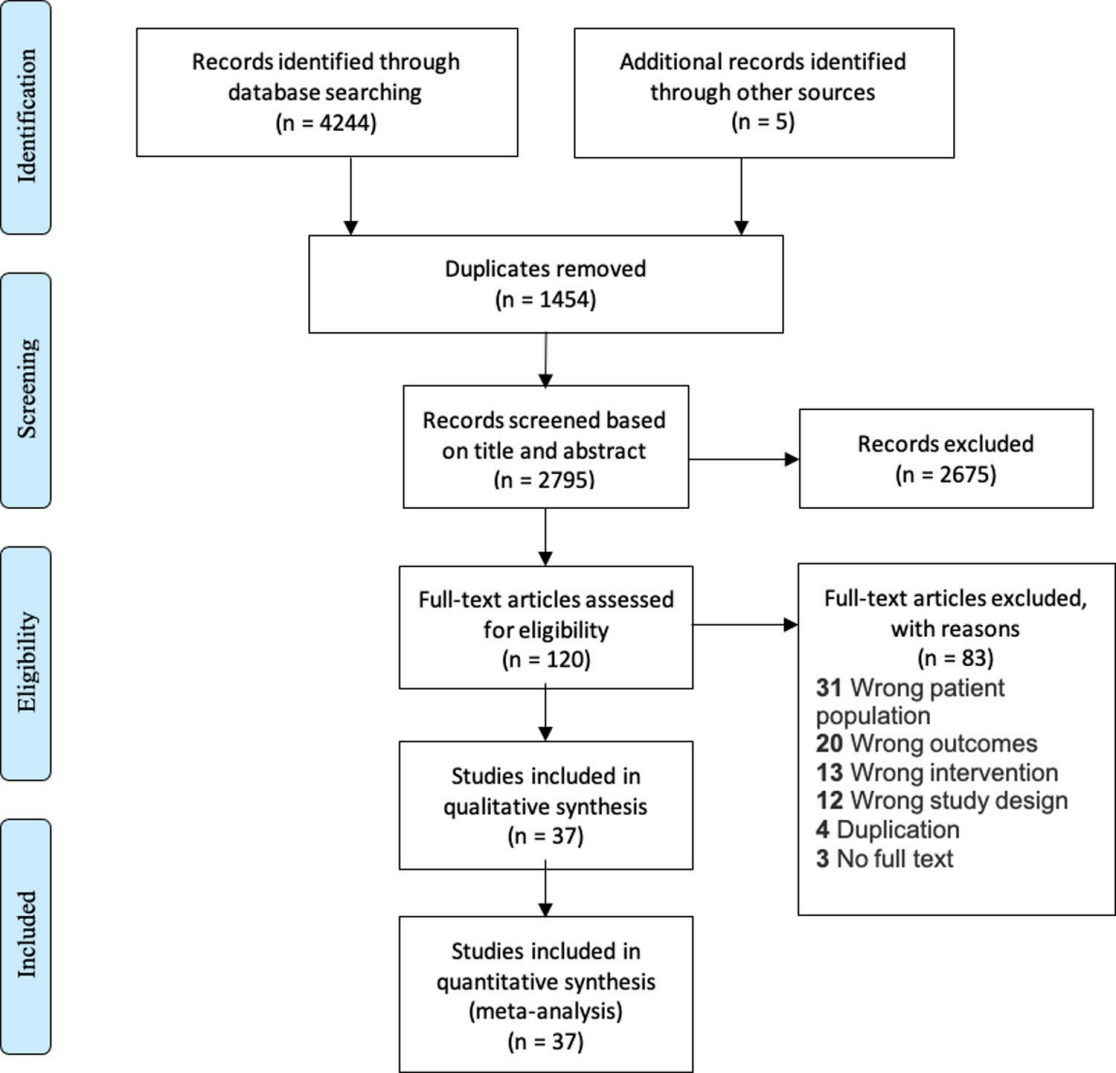
PRISMA diagram of included studies.

**Fig 3:**
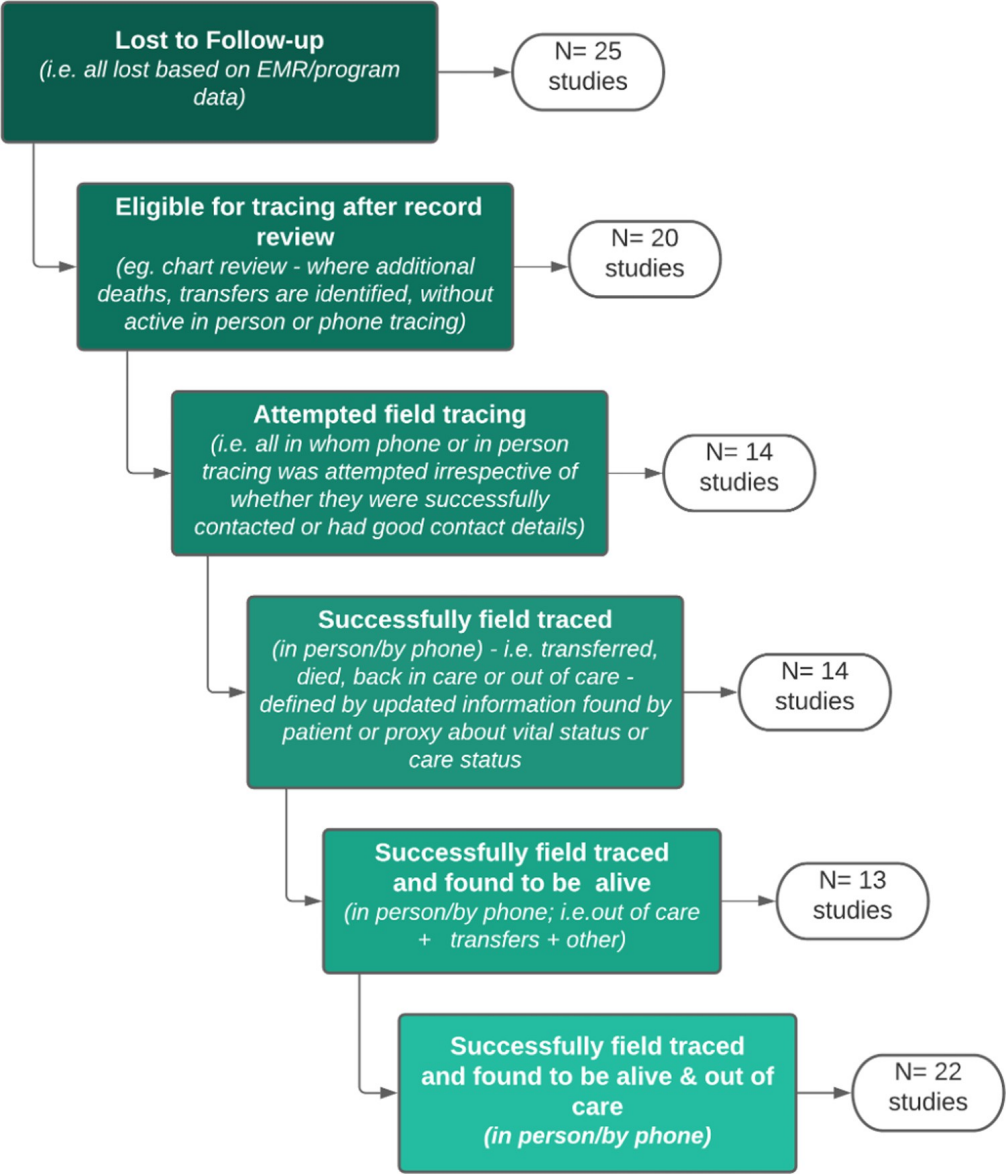
Variations in tracing numerators and denominators presented in included studies (*N* = 37 studies).

We found 4,244 records through our search in the 8 databases plus an additional 5 through other sources (**Fig 2**). We identified 120 records for full-text screening; of these, 37 (6 RCTs and 31 observational) studies met the eligibility criteria, and the other 83 full text articles were excluded for various reasons (**Fig 2**). All 37 studies (**Appendix F in S1 Text**) were included in the qualitative assessment and quantitative meta-analysis of proportions of patients returning to care. Studies reported variable metrics of tracing denominators with the majority reporting the number initially considered LTFU (25 studies), the number considered to be out of care after record review (*N =* 22) and/or the number found to be alive and out of care (*N* = 21) (**Fig 3**). Ten studies had an eligible comparator arm (no reengagement intervention or SOC services), these were included in comparative meta-analyses. Most studies were conducted in high-income countries (HICs) (21 studies, including 18 in the United States of America), recruited both male and female participants (36 studies) and included adults exclusively (20 studies). A total of 112,341 PLWH (60% men) participated in the studies. Nineteen studies included men who have sex with men (MSM) (range 13% to 88%), and 15 studies including people who inject drugs (PWID) (range 4% to 23%). The definition of LTFU varied markedly from any missed visit (4 studies) to no visit in 12 months or more (7 studies) but was most commonly characterized as missing clinic appointments (**Table 1, Appendix D in S1 Text**).

**Table 1:** Characteristics of included studies.

Study	Country	Income	Design	N	Sex	Age	% Male	% MSM	% PWID	LTFU definition
Alamo 2012	Uganda	LMIC	Cohort	579	All	Adult	40%			Absence from the clinic for 90 days after the expected last clinic visit
Alizadeh 2019	Uganda	LMIC	Cohort	691						Missed 2 monthly appointments (either pre- or post-ART initiation)
Ardura-Garcia 2015	Malawi	LMIC	Cohort	251	All	Peds	47%			Missed an scheduled appointment for ART collection of 3 weeks or more
Bean 2017	USA	HIC	Cohort	233	All	Adult	77%			Not retained in care for 1 year
Beres 2019	Zambia	LMIC	RCT	37,933	All	Adult	40%			Visit gap of >90 days from their last appointment
Bershetyn 2017	Uganda, Kenya, Tanzania	LMIC	RCT	5,781	All	Adult	35%			LTFU (>90 days late for last visit)
Bove 2015	USA	HIC	Cohort	1,399	All	Adult	84%	39%	20%	No CD4 or VLs reported for > = 1 year
Bupamba 2010	Tanzania	LMIC	Cohort	966	All					Missing 3 consecutive appointments
Chang 2019	USA	HIC	Cohort	408	All	All	86.0%	55.0%	10.0%	No CD4 or VL reported to surveillance for≥12 months or a VL of>500 copies/mL at last report ≥6 months after HIV diagnosis.
Chikuse 2019	Malawi	LMIC	Cohort	5,651	All					Missed appointment and lost for unknown duration
Deery 2014	South Africa	LMIC	Cohort	755	All		38%			No ART drug pickup within 1 month of the scheduled refill date
Donovan 2018	USA	HIC	Cohort	1,118	All	Adult	70%	40%	9%	Lack of attendance at a medical visit in the prior 6–9 months
Dufour 2018	UK	HIC	Cohort	377	All					Patients not seen in the HIV service for ≥8 months and without future appointments
Edwards 2019	Trinidad and Tobago	LMIC	Cohort	1,058	All	All	50%	12%		Patients who were not active in care for 3 months or more
Fanfair 2019	USA	HIC	RCT	1,894	All	Adult	74%	45%	20%	No CD4 or VL result in surveillance data for ≥6 months and no clinic visit for ≥6 months
Fernández-Luis 2019	Mozambique	LMIC	Cohort	269	All	Children	59.7%			Not attending the clinic for 120 days after last attended visit
Fox 2018	South Africa	LMIC	RCT	1,266	All	Adult	40%			No return for scheduled appointment within 5 to 90 days of appointment date
Healey 2018	Australia	HIC	Cohort	44	All	Adult	87%	80%		Patient not having attended for >4 months
Keller 2017	USA	HIC	Cohort	452	All	Adult	69%	46%	7%	Not attend clinic appointment for >9 months
Kunzweiler 2019	USA	HIC	Cohort	1,418	All	Adult	69.9%	27.7%	22.9%	No CD4 or VL test completed in the previous 6 months] or no CD4 or VL test completed within 3 months of being diagnosed
Lubelcheck 2016	USA	HIC	Cohort	55	All	Adult	73%	41%		No primary care visit in 7 months
Magnus 2012	USA	HIC	Cohort	419	All	Adult	63%	14%	4%	No CD4 or HIV VL monitoring in >1 year
McMahon 2015	Australia	HIC	Cohort	167	All		92%	67%		No VL in 9 months
Nabaggala 2018	Uganda	LMIC	Cohort	381	All		32%			Missed a scheduled visit
Naidoo 2019	South Africa	LMIC	Cohort	864	All	All	27%			1 or more missed monthly or biweekly visits
Nakiwogga-Muwanga 2015	Uganda	LMIC	Cohort	256	All	Adult	41%			Missed clinic appointment for 8–90 days
Rebeiro 2017	Kenya	LMIC	Cohort	34,522	All	Adult	32%			Missed scheduled appointment
Saafir-Callaway 2020	USA	HIC	Cohort	686	All	Adult	68.0%	42.7%	12.6%	No VL result, CD4 result, or care visit for the immediate past 6–12 months
Sharp 2019	USA	HIC	Cohort	166	All	Adult	70.0%			No CD4 or HIV-1 RNA tests during the last 14 months
Sitapati 2012	USA	HIC	Cohort	716	All	All	86%	62%	13%	Gaps in care for more than 6 months
Tesoriero 2017	USA	HIC	Cohort	1,155	All	Adult	61%	37%	14%	No prognostic or diagnostic laboratory results (VL, CD4, or genotype) in the prior 13 to 24 months
Tweya 2010	Malawi	LMIC	Cohort	3,098	All	All	44%			Missed clinic appointments by >3 weeks
Udeagu 2013	USA	HIC	Cohort	797	All	All	55%	15%	35%	No CD4 or VL during the most recent 9 months
Udeagu 2018	USA	HIC	Cohort	1,218	All	Adult	59%	23%	23%	No laboratory reports in the last 9 months
Udeagu 2019	USA	HIC	RCT	3,604	All		52%	21%	10%	No HIV VL or CD4 cell count > = 9 months
Villanueva 2019	USA	HIC	RCT	655	All		62%	30%	31%	“In-care” for 12 months followed by “out-of-care” for 6 months
Wohl 2016	USA	HIC	Cohort	1,139	All	Adult	78%	50%	33%	No HIV care visits in 6–12 months and last VL >200 copies per milliliter or no HIV care visits in 12 months

ART, antiretroviral therapy; HIC, high-income country; LMIC, low- and middle-income country; LTFU, lost to follow-up; MSM, men who have sex with men; PWID, people who inject drugs; RCT, randomized controlled trial; VL, viral load.

### Description of reengagement interventions

Reengagement programs conducted a diverse set of activities (**Table 2, Appendix D in S1 Text**), which varied by setting. Most programs used a combination of text messages, telephone calls, and in-person tracing to identify outcomes among lost patients and reengage those who were out of care. Tracers included peers, social workers, and other healthcare workers. The vast majority contacted and provided encouragement or counseling to those patients encountered who were out of care, but others reported the use of monetary incentives or transportation aid (11 studies), telephone calls (29 studies), letters (11 studies), or emails (7 studies). The number of tracing attempts and the time to initiation of tracing after identification of LTFU status were infrequently reported.

**Table 2:** Reengagement intervention implementation characteristics.

Study	Income	Tracing method	Trace by telephone call	Trace by letter	Trace by home visit	Trace by email	Trace by nonprimary visit*	Support strategy	Tracer	Tracing attempts	Time to tracing	Control	Outcome time point	Time point start date
**Alamo 2012**	LMIC	In-person	No	No	Yes	No	No		Peer	1 or 2	Same day as missed apt	None	18 months or longer	after LTFU
**Alizadeh 2019**	LMIC	In-person	No	No	Yes	No	No		Outreach worker	1		None	Unknown	
**Ardura-Garcia 2015**	LMIC	In-person + call	Yes	No	Yes	No	No		Outreach worker	≥3	1 w after LFTU	None	3 months	
**Beres 2019**	LMIC	In-person + call + reengagement support	Yes	No	Yes	No	No	visit escort	Peer	≥3	15 m (median) after LTFU	None	18 months or longer	
**Bershetyn 2017**	LMIC	In-person	No	No	Yes	No	No		Peer			SOC (no tracing)	12 months	after sampling
**Bupamba 2010**	LMIC	In-person + call + reengagement support	Yes	No	Yes	No	Yes	visit escort	Peer			None	6 months	
**Chikuse 2019**	LMIC	In-person + call	Yes	No	Yes	No	No		Peer			None	3 months or less	
**Deery 2014**	LMIC	In-person + call	Yes	No	Yes	No	No		Lay HCW	1 or 2		None	3 months or less	after home visit
**Edwards 2019**	LMIC	Phone/text/email/mail	Yes	No	No	No	No		SW	≥3		None	6 months	
**Fernández-Luis 2019**	LMIC	Phone/text/email/mail	Yes	No	No	No	No		Lay HCW	1		None	3 months or less	after intervention enrollment
**Fox 2018**	LMIC	In-person + call	Yes	No	Yes	No	No		Outreach worker		5–90 days after missed apt	SOC (no tracing)	3 months or less	
**Nabaggala 2018**	LMIC	In-person + call	Yes	No	Yes	No	No		Lay HCW	≥3		None	3 months or less	after contact
**Naidoo 2019**	LMIC	In-person	No	No	Yes	No	No		Community HCW	1		None	Unknown	
**Nakiwogga-Muwanga 2015**	LMIC	In-person + call	Yes	No	Yes	No	No		Lay HCW			None	3 months	after tracking
**Rebeiro 2017**	LMIC	In-person	No	No	Yes	No	No		Lay HCW		Early as 8 days after LTFU	SOC (Patients not found)	12 months	After missed appointment
**Tweya 2010**	LMIC	In-person + call	Yes	No	Yes	No	No		Lay HCW	≥3	>3 weeks after missed appointment	None	Unknown	
**Bean 2017**	HIC	In-person + call	Yes	Yes	Yes	No	No		Outreach coordinator			None	Unknown	
**Bove 2015**	HIC	In-person + call + reengagement support	Yes	No	No	Yes	Yes	visit escort, transportation assistance, or inpatient visits	Linkage specialist/case manager	≥3		SOC (no tracing)	12 months	
**Chang 2019**	HIC	Call + reengagement support	Yes	No	No	No	No	rescheduling new appointment	HCW			None	12 months	after intervention enrollment
**Donovan 2018**	HIC	In-person + call	Yes	Yes	No	No	Yes		Patient navigator/case manager			None	3 months or less	after contact
**Dufour 2018**	HIC	Phone/text/email/mail	Yes	No	No	No	No			1 or 2		None	Unknown	
**Fanfair 2019**	HIC	In-person							Disease intervention specialist			SOC (no tracing)	3 months or less	after randomization
**Healey 2018**	HIC	Phone/text/email/mail	Yes	Yes	No	Yes	No		SW			None	Unknown	
**Keller 2017**	HIC	In-person + call + reengagement support	Yes	Yes	No	No	No	Transportation assistance, and mental health and substance use services	Patient navigator/case manager			None	12 months	
**Kunzweiler 2019**	HIC	In-person + call + reengagement support	Yes	Yes	Yes	Yes	No	rescheduling new appointment	Field epidemiologist	1		None	3 months or less	after intervention enrollment
**Lubelcheck 2016**	HIC	In-person + call	Yes	No	No	No	Yes		Program coordinator		At attendance for non-HIV care	None	3 months	after alert
**Magnus 2012**	HIC	EMR alert + provider decision support					No		HCW			SOC (no tracing)	6 months	
**McMahon 2015**	HIC	Phone/text/email/mail	Yes	No	No	No	No					None	6 months	
**Saafir-Callaway 2020**	HIC	In-person + call	Yes	Yes	Yes	Yes	No					SOC (no tracing)	18 months or longer	After tracking
**Sharp 2019**	HIC	In-person + call	Yes	No	No	No	Yes		HCW/SW			SOC (no tracing)	6 months	After tracking
**Sitapati 2012**	HIC	Phone/text/email/mail	Yes	Yes	No	Yes	No		Retention specialist	≥3	Unspecified; out of care defined by a gap of 6 or more months	None	6 months	
**Tesoriero 2017**	HIC	In-person + call	Yes	Yes	Yes	No	No		Disease intervention specialist			None	6 months	
**Udeagu 2013**	HIC	In-person + call	Yes	Yes	Yes	No	No		Case workers			None	12 months	
**Udeagu 2018**	HIC	In-person + call	Yes	Yes	Yes	Yes	No		Case workers			None	12 months	
**Udeagu 2019**	HIC	In-person + call	Yes	No	Yes	No	No		Patient navigator			SOC (no tracing)	18 months or longer	
**Villanueva 2019**	HIC	In-person					No		Disease intervention specialist			SOC (no tracing)	3 months or less	after randomization
**Wohl 2016**	HIC	In-person + call + reengagement support	Yes	Yes	Yes	Yes	No	Assist scheduling and emergency referral	Navigator	≥3		None	12 months	after intervention enrollment

EMR, electronic medical record; HCW, healthcare worker; HIC, high-income country; LMIC, low- and middle-income country; LTFU, lost to follow-up; SOC, standard of care; SW, xxxx.

More specifically, in low- and middle-income country (LMIC) settings (16 studies), identification of those LTFU usually involved exploring data from electronic medical record (EMR) datasets combined with paper record chart reviews. Tracing most frequently included a combination of telephone calls and active (in-person) tracing involving a home visit to locate the patient and encourage return. Lack of telephones or incorrect telephone numbers identified in several studies necessitated home visits [26,27]. In-person field tracing frequently required transport subsidies [28], motorcycles [29], or cars and drivers for tracers to travel widely. In this setting, tracing was most commonly conducted by outreach teams including staff trained specifically to conduct outreach termed community health workers, lay health workers, or outreach workers who were frequently peers. Of the 10 LMIC studies that reported on the frequency of tracing attempts, 6 reported more than 3 tracing attempts, though it was variable as to whether these were telephone or in-person/field tracing attempts. The majority of LMIC studies initiated tracing efforts within 3 months of a missed visit, but this ranged from starting on the day of a missed visit to up to 15 months later. Two studies described additional support for reengagement in care after contact; in one case, tracers would accompany patients on their first visit back to the clinic if requested to do so, and in another peer tracers routinely assisted patients with navigation within the clinic during return visits [30].

In high-income country (HIC) settings (21 studies), identification of LTFU similarly involved exploring data from EMR data systems and cross-comparison of EMR data with individual medical records and other clinic record systems to identify those truly disengaged from care. Several studies in this setting additionally matched EMR visit data with other local public health surveillance data to determine if patients had reengaged in care elsewhere, had died, or were imprisoned [28,31–33]. Tracing included telephone calls in almost all cases, frequently combined with a letter reminding patients of their missed visit, and in some cases emails. Home visits were less frequent than in LMICs and only reported in 7 studies. In another system used in 3 studies, providers received an alert either through the EMR system or through text message when an identified out-of-care PLWH attended a non-HIV clinical visit in another service, allowing providers to initiate relinkage [10,34,35]. Tracing and the reengagement processes were most commonly conducted by a linkage specialist, case manager, tracer, or patient navigator, essentially staff who were focused on conducting outreach services and supporting patients; this frequently involved both a tracer and a case manager. Additional support for reengagement included transport assistance in the form of transport subsidies or transport provision, assistance with rescheduling clinic appointments, and in one case the additional provision of substance abuse [36] and mental health services [32].

### Methodological quality and external validity for comparative analysis

We used the Cochrane ROB-1 tool and the Newcastle–Ottawa tool to assess the risk of bias in RCTs (*N* = 6) and comparative cohort studies (*N* = 5). The majority of RCTs were assessed as low risk (*N* = 3) or of some concern (*N* = 3). Poor reporting of research methods resulted in an incomplete assessment of several studies (**Table 3**).

**Table 3:** Risk of biases in the included RCTs.

Study	Outcome	Sequence Generation	Allocation Concealment	Blinding Participants/Personnel	Blinding Outcome Assessor	Attrition Bias	Selective Reporting	Other Bias	Overall ROB
Udeagu 2019	Return to care	Unclear risk	Unclear risk	Unclear risk	Unclear risk	Low risk	High Risk	Low risk	Some concerns
Villanueva 2019	Return to care	Unclear risk	Unclear risk	Unclear risk	Unclear risk	Unclear risk	Low Risk	Low risk	Some concerns
Villanueva 2019	Viral suppression	Unclear risk	Unclear risk	Unclear risk	Unclear risk	Unclear risk	Low Risk	Low risk	Some concerns
Fox 2018	Return to care	Low risk	Unclear risk	Unclear risk	Unclear risk	Low risk	Low Risk	Low risk	Low risk
Fox 2018	Retention in Care	Low risk	Unclear risk	Unclear risk	Unclear risk	Low risk	Low Risk	Low risk	Low risk
Beres 2019	Return to care	Low risk	Unclear risk	Unclear risk	Unclear risk	Low risk	Low Risk	Low risk	Low risk
Bershetyn 2017	Return to care	Low risk	Unclear risk	Unclear risk	Unclear risk	Low risk	Low Risk	Low risk	Low risk
Fanfair 2019	Return to care	Unclear risk	Unclear risk	Unclear risk	Unclear risk	Unclear risk	Low Risk	Low risk	Some concerns

RCT, randomized controlled trial; ROB, risk of bias.

The majority of cohort studies were assessed as good quality after application of risk of bias tools; studies were assessed as fair quality or poor quality primarily due to the use of a historical comparison group, and in a few cases, there was minimal record review or a long lag time prior to tracing resulting in lack of clarity as to whether participants had already returned prior to tracing efforts (**Table 4**). The risk of bias for the included studies by the reported outcome is presented below. Detailed risk of bias assessments can be found in **Appendix B in S1 Text**.

**Table 4:** Risk of biases in the included cohort studies that had a control group.

Study	Outcome	Selection	Comparability	Outcome	Total	Overall ROB
Magnus 2012	Return to care	****	*	***	********	Good Quality
Magnus 2012	Retention in Care	****	*	***	*******	Good Quality
Bove 2015	Return to care	***	*	***	******	Good quality
Bove 2015	Viral suppression	**	*	**	*****	Fair Quality
Bove 2015	Retention in Care	***	*	***	******	Good Quality
Rebeiro 2017	Return to care	**	*	*	****	Poor Quality
Sharp 2019	Return to care	**	*	***	*****	Fair Quality
Saafir-Callaway 2020	Retention in Care	****	*	***	*******	Good Quality
Saafir-Callaway 2020	Retention in Care	****		***	*******	Good Quality
Saafir-Callaway 2020	Viral suppression	****	*	**	******	Good Quality

ROB, risk of bias.

Overall, studies were highly pragmatic—conducted in real-world settings (**Table 5**), with flexible approaches to intervention delivery and few additional measures to trace patients beyond what would occur in routine practice. Several studies were downgraded from highly pragmatic (score of 5) to lower scores due to the organization providing more resources than would be available in a real-world setting or the restriction of those who were traced to a specific subset of those lost (e.g., out of care for <6 months or no pregnant women). Details of PRECIS-2 scores are presented in **Appendix C in S1 Text.**

**Table 5:** PRECIS criteria/score.

Study	Eligibility	Recruitment/cohort selection	Setting	Organization	Flexibility: delivery	Flexibility: adherence	Follow-up	Primary Outcome	Primary Analysis
Beres 2019	5	5	5	5	4	5	5	5	5
Bershetyn 2015	5	5	5	4	5	5	5	5	5
Bove 2015	5	4	4	4	5	5	5	5	5
Fanfair 2019	4	5	5	2	5	5	5	5	5
Fox 2018	4	5	4	4	4	5	5	5	4
Magnus 2012	5	5	5	4	5	5	5	5	NA
Rebeiro 2017	4	5	5	5	5	5	5	5	3
Udeagu 2019	5	5	5	4	4	5	5	5	4
Villanueva 2019	3	5	5	3	5	5	5	5	5
Sharp 2019	5	4	3	4	5	5	5	5	3

*Darker colors represent pragmatic approaches; lighter colors represent explanatory approaches.

NA, not applicable.

### Reengagement program outcome: Return to original clinic among all identified as lost-to-follow-up

**Fig 4:**
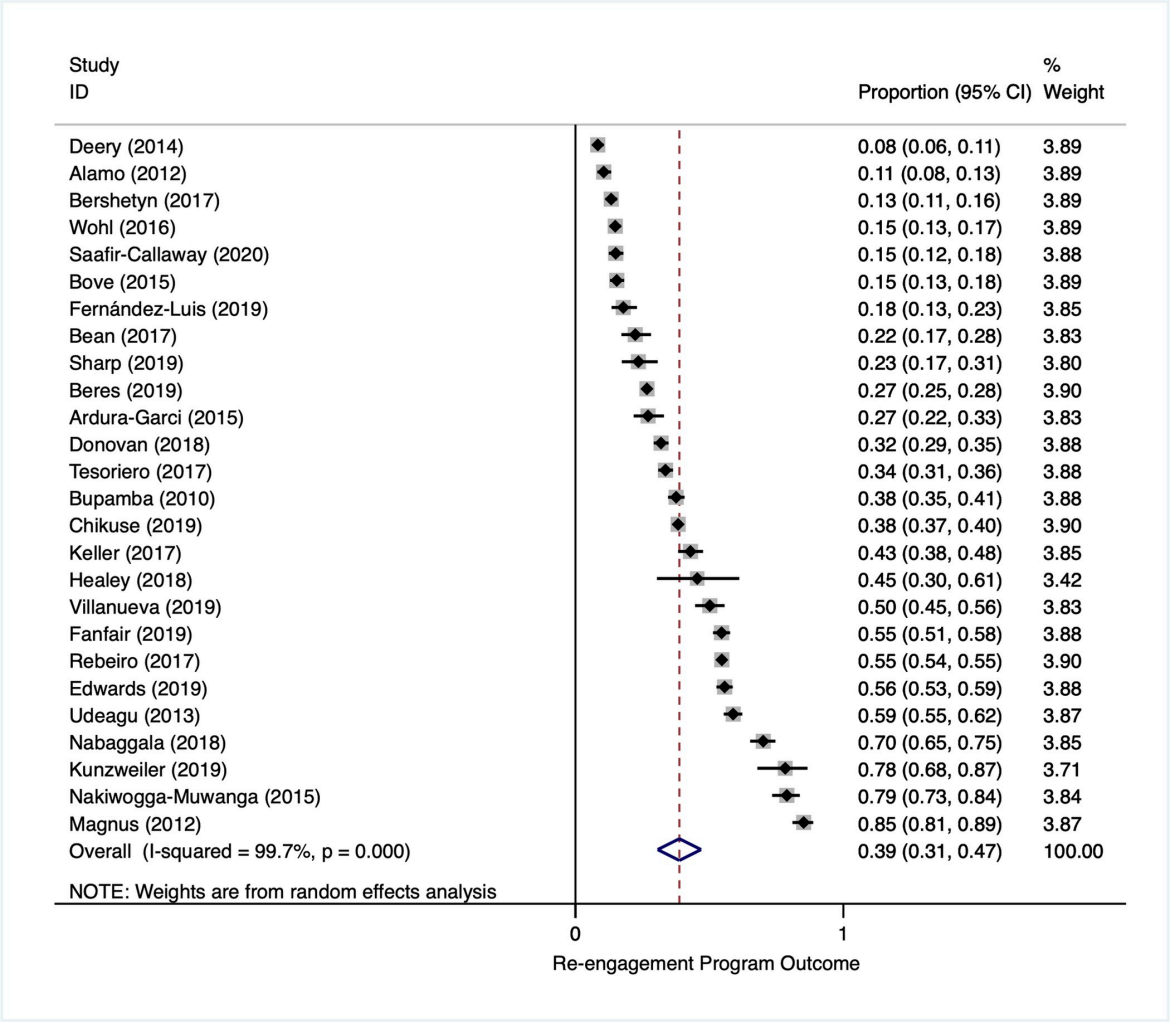
Reengagement program outcome: proportion returned to care among all identified as LTFU (in intervention study arms). LTFU, lost to follow-up.

Overall, 26 studies contributed to the descriptive estimate of the fraction of patients who were identified as LTFU that subsequently returned to the original clinic in settings with any kind of effort to identify and contact those who had not returned as expected to clinic for HIV treatment. Overall, across all studies, 39% (95% CI: 31% to 47%) of all patients who were LTFU in reengagement intervention arms returned to care (**Fig 4**). There was, however, substantial heterogeneity in the proportion who returned.

This heterogeneity persisted in subgroup analyses (**Table 6**). There appeared to be no large differences in subgroup estimates of the proportion who returned to care by study design, country income level, tracing method, time to tracing, or timing of the outcome measure. There did appear to be slightly higher reengagement in studies where tracing occurred after a minimum of one missed visit (0.48; 95% CI: 0.23 to 0.73) as compared to studies where patients were LTFU for longer periods, particularly those out of care for 12 months or more (0.32; 95% CI: 0.13 to 0.52).

**Table 6:** Reengagement program outcome: Proportion of all patients who were LTFU returned to the clinic by subgroups.

Subgroups	Number of studies	Proportion returned (95% CI)*	*P* value**	I-squared***
**Overall**	26	0.39 (0.31, 0.47)	0.001	99.70%
**Study Design**				
Observational	22	0.39 (0.30, 0.48)	0.001	99.70%
Randomized	4	0.36 (0.20, 0.52)	0.001	99.40%
**Country income**				
High income	14	0.41 (0.29, 0.52)	0.001	99.30%
Low–middle income	12	0.37 (0.24, 0.49)	0.001	99.80%
**Tracing type**				
Phone/text/email/mail	3	0.40 (0.10, 0.69)	0.001	98.80%
In-person tracing	5	0.37 (0.14, 0.59)	0.001	99.80%
In-person tracing + Call	11	0.37 (0.25, 0.49)	0.001	99.40%
Call + reengagement support	0	----	----	----
EMR alert + decision support	1	0.85 (0.81, 0.89)	----	----
In-person tracing + Call + reengagement support	6	0.35 (0.25, 0.45)	0.001	98.70%
**Time when tracing started after LTFU**				
Tracing started at 30 days or sooner	3	0.31 (0.01, 0.64)	0.001	99.80%
Tracing started after 30 days	1	0.27 (0.25, 0.28)	----	----
Unknown	22	0.40 (0.32, 0.49)	0.001	99.40%
**Outcome measured at**				
3 months or less	10	0.45 (0.32, 0.59)	0.001	99.40%
6 months	5	0.47 (0.28, 0.66)	0.001	99.30%
12 months	6	0.33 (0.13, 0.54)	0.001	99.80%
18 months or longer	3	0.17 (0.07, 0.28)	0.001	98.70%
Unknown	2	0.33 (0.10, 0.55)	0.001	87.00%
**Definition of LTFU**				
1 or more missed monthly or biweekly visits	5	0.48 (0.23, 0.73)	0.001	99.80%
No visit in 3 < 6 months	7	0.29 (0.18, 0.40)	0.001	99.20%
No visit in 6 < 12 months	7	0.47 (0.33, 0.62)	0.001	99.00%
No visit in 12 months or longer	6	0.32 (0.13, 0.52)	0.001	99.50%
Unknown duration	1	0.38 (0.37–0.40)	----	----
**Number of tracing attempts**				
1	2	0.48 (0.01, 0.99)	0.001	99.20%
2	2	0.09 (0.07, 0.11)	0.192	41.20%
3 or more	6	0.35 (0.21, 0.49)	0.001	99.40%
Unknown	16	0.43 (0.34, 0.52)	0.001	99.50%
**Who traced the patient**				
Peer	5	0.25 (0.15, 0.36)	0.001	99.30%
Social worker	9	0.30 (0.17, 0.43)	0.001	99.30%
Healthcare worker (i.e., nurse, physician)	12	0.51 (0.40, 0.61)	0.001	99.30%
**Intervention subtype**				
Active (tracing included any support)	6	0.35 (0.25, 0.45)	0.001	98.70%
Passive (no support reported)	20	0.40 (0.30, 0.49)	0.001	99.70%

*Random effect model.

**P value is for Heterogeneity. H0: Variation is only by chance.

***The variation in the proportion (outcome) attributable to heterogeneity.

CI, confidence interval; EMR, electronic medical record; LTFU, lost to follow-up.

**Fig 5:**
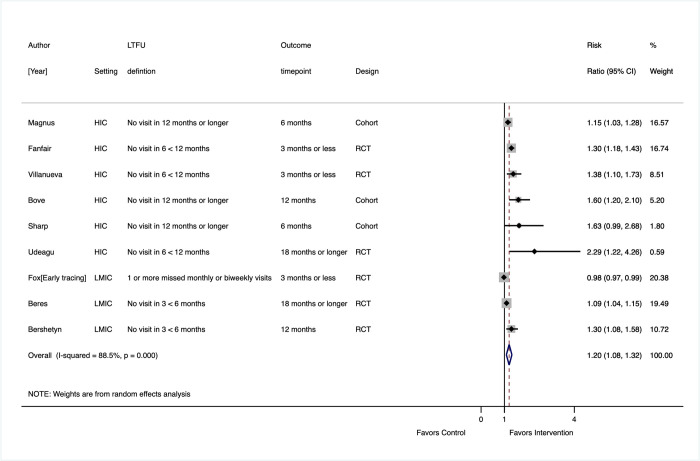
Reengagement program effects (proportion LTFU returned to original clinic), reengagement program versus no intervention or SOC. CI, confidence interval; HIC, high-income country; LMIC, low- and middle-income country; LTFU, lost to follow-up; RCT, randomized controlled trial; SOC, standard of care.

In 9 studies (3 observational and 6 randomized) that had a control condition (i.e., SOC or no tracing) (**Fig 5**), the relative risk of return to care among those traced and found out of care was 1.20 (95% CI: 1.08, 1.32). This effect was stronger in RCTs (RR 1.17, 95% CI: 1.04, 1.31) compared to observational studies (RR 1.36, 95% CI: 0.99, 1.72) and in HIC settings (RR 1.30, 95% CI: 1.16, 1.44) compared to LMICs (RR 1.07, 95% CI: 0.96, 1.19). While the pooled estimate showed effectiveness, these size of effect estimates from individual studies varied substantially (I^2^ 88.5%). One cluster RCT conducted in 24 clinics across 4 provinces in South Africa assessed the effect of an early field tracing intervention integrated into routine clinic practice with minimal oversight and found no evidence of benefit compared to SOC [37]. There was, however, lack of comparability of the intervention and control arms in this study; those who were traced had been out of care for longer period (85 days) compared to controls (29 days). In contrast, a US-based study reporting the highest intervention effect (RR 2.29) included a multistep tracing process, with extensive work done to obtain locating information and the use of both navigators and disease intervention specialists, as well as biweekly case management meetings of staff to address challenges [32].

Heterogeneity of effects persisted within all subgroup analyses, including by risk of bias assessment and implementation characteristics (**Appendix E in S1 Text**).

### Reengagement contact outcome (return to original clinic among those found to be alive and out of care)

**Fig 6:**
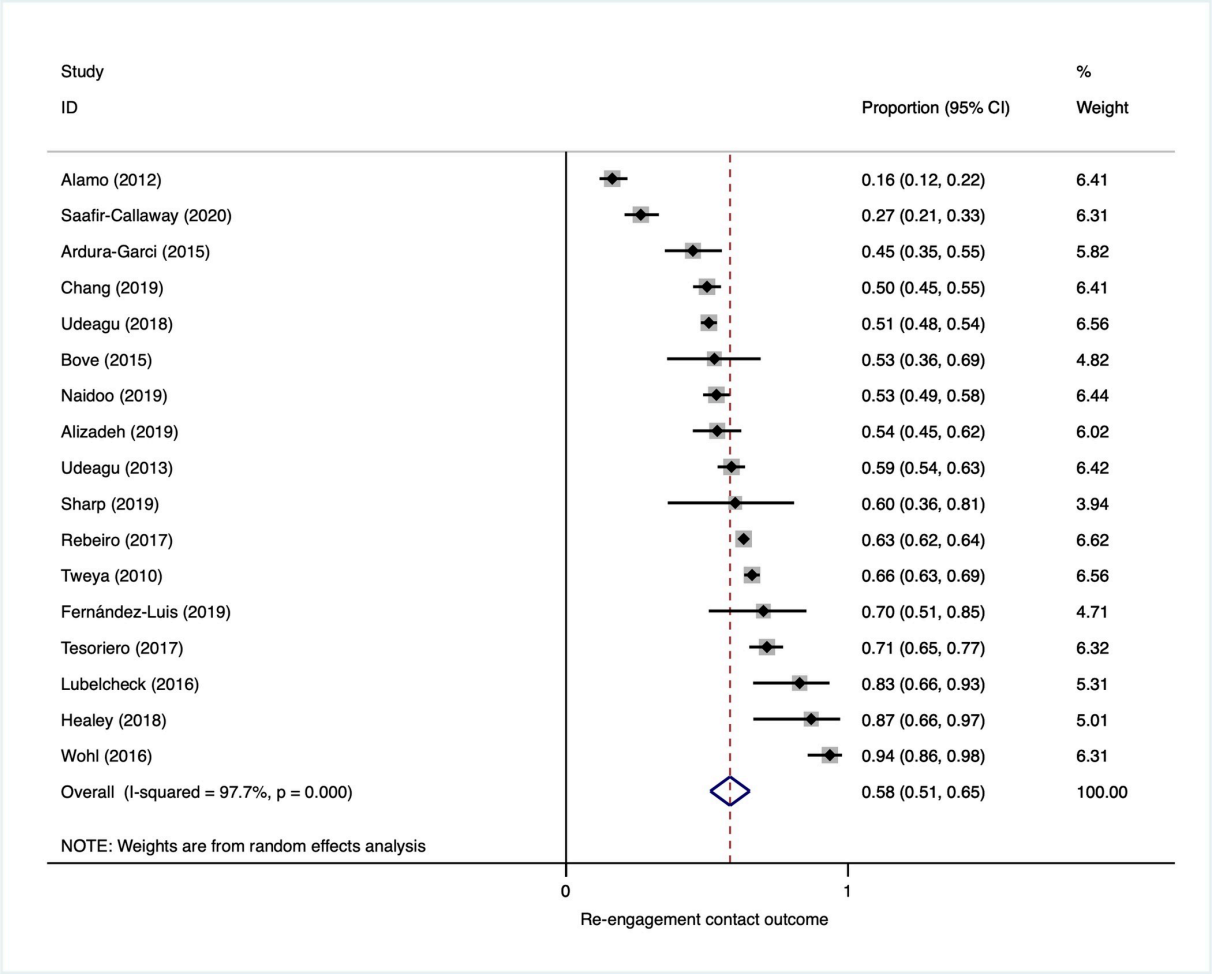
Reengagement contact outcome: proportion found alive and out of care who returned to care (in reengagement intervention study arms).

Among those who were found to be alive and out of care through reengagement program efforts (17 studies), 58% reengaged in care after being contacted in person or by telephone (95% CI, 51% to 65%) (**Fig 6**). There was similarly marked heterogeneity in this analysis with return to care ranging from 16% to 94%, making the pooled estimate less relevant to any particular program or setting. Heterogeneity was explored in subgroup analysis; this, however, did not explain heterogeneity (**Appendix E in S1 Text**).

**Fig 7:**
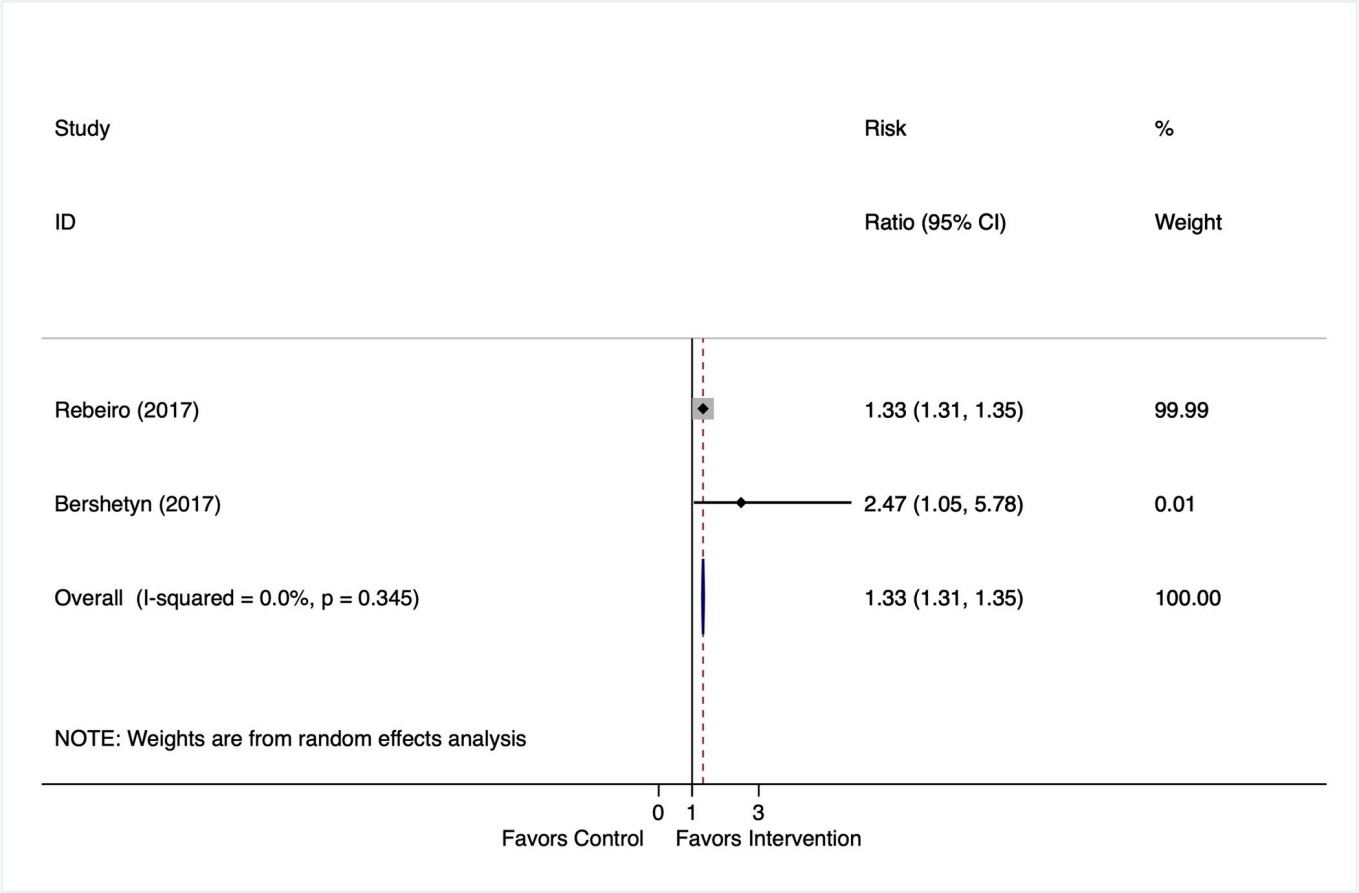
Reengagement contact effects: proportion returned to care among patients who were contacted and found out of care in comparative studies.

In 2 studies that had a control condition and evaluated the reengagement contact outcome (**Fig 7**), return to care was greater among those in the reengagement intervention arm as compared to SOC or not tracing (RR 1.33, 95% CI: 1.31, 1.35).

### Retention in care

**Fig 8:**
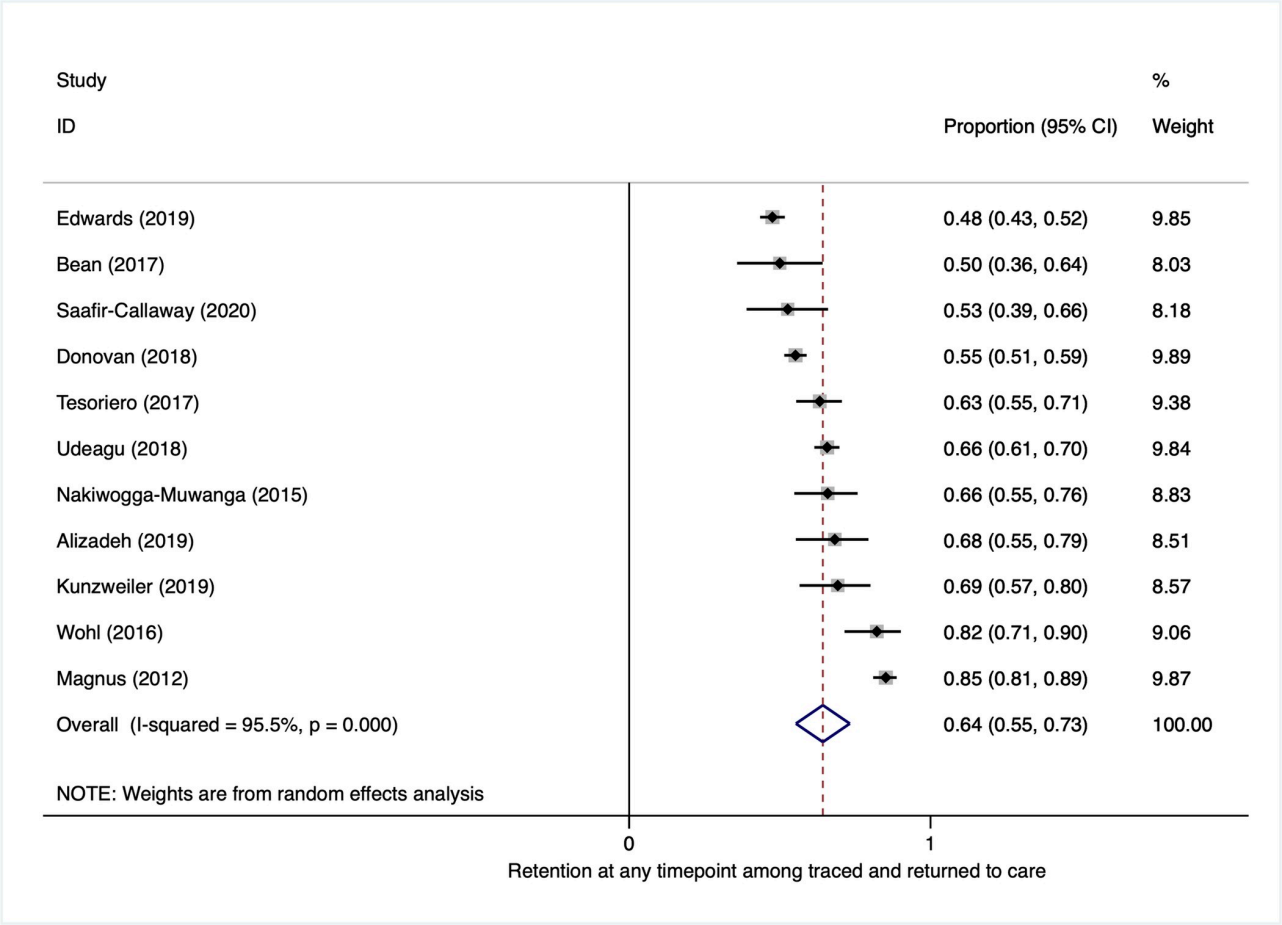
Proportion retained in care after return to care.

Overall, the proportion retained among PLWH who were traced and subsequently returned to care at any time after becoming lost to care (range 48% to 64%) was 64% (95% CI: 55 to 73) (**Fig 8**). This proportion was consistent across subgroups (**Appendix E in S1 Text**).

Two studies that had a control condition reported comparative estimates; however, due to the variation in the outcome definitions and marked difference in retention estimates, data could not be pooled for comparative assessment. One RCT in South Africa [37] showed no difference in 12-month retention between those who had missed a visit by 5 days or more in the intervention arm compared to the control arm (RR 0.88, 95% CI: 0.75, 1.04). Another cohort study [28] showed better retention (≥2 consecutive visits ≥3 months apart) in the reengagement arm (adjusted RR 2.4, 95% CI: 1.5, 3.9) compared to a historical cohort in the US.

### Viral suppression

**Fig 9:**
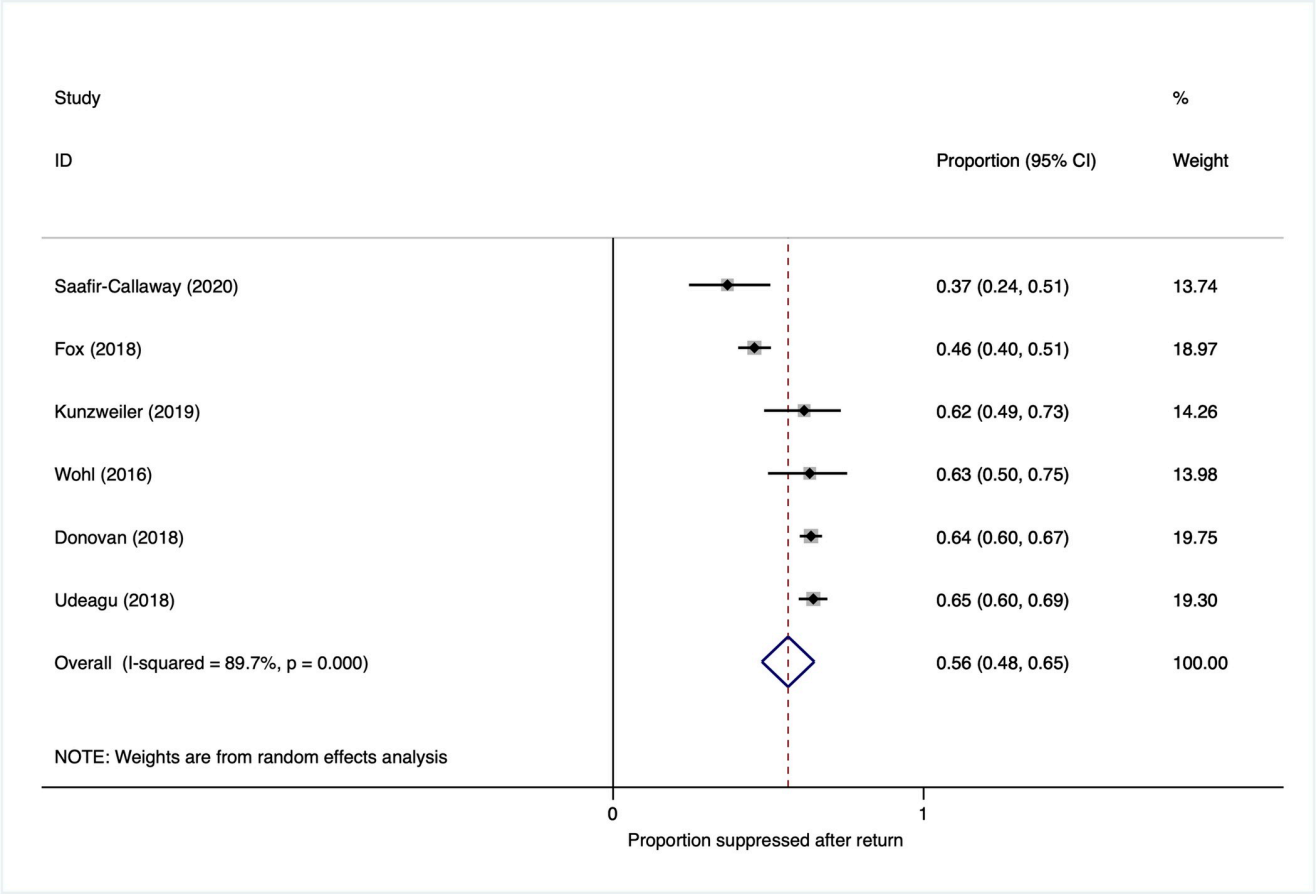
Proportion virally suppressed after return to care.

Overall, viral suppression among PLWH who were traced and subsequently returned to care was 56% (95% CI, 48% to 65%) (**Fig 9**). As with all analyses, there was substantial heterogeneity of viral suppression rates, which persisted within subgroup analysis by country income, tracing methods, and LTFU definition (**Appendix E in S1 Text**).

One study reported a risk ratio comparing the proportion virally suppressed (among PLWH who were traced and subsequently returned to care) in the reengagement intervention versus a preintervention historical control, showing no difference in viral suppression after return to care RR: 1.60 (95% CI: 0.97 to 2.60) [28].

### Mortality

We identified 2 cohort studies that reported mortality after return to care. In one, the 18-month mortality risk among individuals after returning to care was 5% (4/85), compared to 2% (2/117) in a historical cohort (RD 3%, 95% CI: −2, 8%) [27]. In another, the mortality rate was reported as 4% at 1 year, 6% at 2 years, 10% at 3 years, 11% at 4 years, and 14% at 5 years after reengagement among individuals returning to care [38].

### GRADE evidence certainty

To establish the overall certainty of the evidence contributing to the pooled comparative estimates, we applied the GRADE framework across 5 domains (**Table 7**). For the main analysis of the reengagement program effect (return to care at original clinic among all LTFU), there was low certainty that reengagement interventions may improve return to care. This overall program effect was downgraded twice due to marked qualitative and quantitative heterogeneity of reengagement strategy features, contexts, and effect estimates. Data restricted to HIC settings was, however, graded as high certainty evidence as findings within this subgroup were more consistent than those seen in the heterogenous and limited data from LMIC settings. The reengagement contact outcome was assessed in a few studies with the majority of the pooled estimate driven by one observational study resulting in an overall GRADE assessment of very low certainty evidence for this outcome. Details of PRECIS-2 scores are presented in **Appendix C on S1 Text.**

**Table 7:** GRADE evidence profile.

Certainty assessment							№ of patients		Effect		Certainty	
№ of studies	Study design	Risk of bias	Inconsistency	Indirectness	Imprecision	Other	reengagement interventions (tracing or certain types of interventions)	SOC or not tracing	Relative (95% CI)	Absolute (95% CI)		
**Reengagement program effects (effectiveness): return to care among LTFU (reengagement intervention vs no reengagement intervention)**												
9	randomized trials	not serious	very serious ^a^	not serious	not serious	none	16,924/26,749 (63.3%)	24,168/61,415 (39.4%)	**RR 1.20** (1.08 to 1.32)	**79 more per 1,000** (from 31 more to 126 more)	⨁⨁◯◯ LOW	
**Reengagement program effects (effectiveness): return to care among LTFU (reengagement intervention vs no reengagement intervention)—RCT**												
6	randomized trials	not serious	very serious^a^	not serious	not serious	none	16,499/25,557 (64.6%)	23,695/60,114 (39.4%)	**RR 1.17** (1.04 to 1.31)	**67 more per 1,000** (from 16 more to 122 more)	⨁⨁◯◯ LOW	
**Reengagement program effects (effectiveness): return to care among LTFU (reengagement intervention vs no reengagement intervention)—Observational**												
3	Observational studies^b^	not serious	serious^a^	not serious	not serious	none	425/1,192 (35.7%)	473/1,301 (36.4%)	**RR 1.36** (0.99 to 1.72)	**131 more per 1,000** (from 4 fewer to 262 more)	⨁◯◯◯ VERY LOW	
**Reengagement program effects (effectiveness): return to care among LTFU (reengagement intervention vs no reengagement intervention)—LMIC**												
3	randomized trials	not serious	serious^c^	not serious	serious^d^	none	15,802/24,078 (65.6%)	22,214/55,440 (40.1%)	**RR 1.07** (0.96 to 1.99)	**28 more per 1,000** (from 16 fewer to 397 more)	⨁⨁◯◯ LOW	
**Reengagement program effects (effectiveness): return to care among LTFU (reengagement intervention vs no reengagement intervention)—HIC**												
6	randomized trials	not serious	not serious	not serious	not serious	none	1122/2671 (42.0%)	1954/5975 (32.7%)	**RR 1.30** (1.16 to 1.44)	**98 more per 1,000** (from 52 more to 144 more)	⨁⨁⨁⨁ HIGH	
**Reengagement contact outcome (efficacy): return to care among those among traced and found out of care (reengagement intervention vs no reengagement intervention)**												
2	observational studies^b^	serious^e^	not serious	serious^f^	not serious	none	12,397/16,322 (76.0%)	9,839/20,390 (48.3%)	**RR 1.33** (1.31 to 1.35)	**159 more per 1,000** (from 150 more to 169 more)	⨁◯◯◯ VERY LOW	
***Explanations*** a. Marked unexplained heterogeneity of effect estimates between studies and within subgroups. b. Observational studies automatically downgraded by one point in the GRADE system. c. Marked unexplained heterogeneity with one study (Fox) showing worse outcomes in the intervention group. d. Wide confidence interval including no effect and benefit. e. Rebeiro 2017 used imputed vital status values from those who were contacted to revise outcomes for those who were not found and the remaining untraced clinic population. This relies on the assumption that those who are not found/not traced early will have the same vital status as those who were found within 8 days which may not be valid. f. Although 2 included studies, most of the estimate is based on data from only one study, which may not be externally valid for all settings.												

CI, confidence interval; HIC, high-income country; LMIC, low- and middle-income country; LTFU, lost to follow-up; RCT, randomized controlled trial; RR, risk ratio; SOC, standard of care.

## Discussion

This systematic review, which included 21 studies from HICs and 16 studies from LMICs, found that across settings reengagement programs resulted in 39% of PLWH categorized as LTFU at baseline reengaging in care at their original clinic at any time point. We also found that among those who were truly disengaged (alive and out of care) a higher percentage (58%) returned. Compared to the SOC, reengagement interventions resulted in 20 percentage greater return to care beyond what would have happened in routine practice. Findings were more robust for HIC settings and RCTs, but even within these subgroups, there was substantial heterogeneity of estimates across individual studies; this heterogeneity persisted in subgroup analyses by study design and implementation characteristics. Studies used a variety of methods to establish LTFU status prior to tracing, with most reviewing EMR data and in some cases cross referencing EMR records against other public records such as death registries and prison records. Definitions of LTFU ranged from one or more missed biweekly visits to being out of care for 12 months or longer. Telephone calls combined with field tracing were conducted in the majority of studies; this was, however, more common in LMICs and HICs used more passive approaches after telephone calls, such as email and mail, to contact patients in some instances. Overall studies reported few additional support measures beyond encouraging return to care, with only 4 studies facilitating the return visit with transport vouchers or navigation and one study reporting addressing long-term barriers to retention.

These data suggest that, to enhance the efficiency of reengagement programs, it will be critical for health services to invest in systems to improve recording of transfers and deaths. Across studies, efforts to reengage patients who were out of care invariably started with activities to enumerate those who had not returned as expected and identify (through filtering out transfers and deaths) those who were actually alive and not in care. Many patients who had not returned resulted from undocumented deaths, or unknown transfers to another facility. Therefore, in many cases, those who had “truly disengaged” (were alive and out of care) and who were the true targets of reengagement activities were a relatively small number of the total identified as LTFU (i.e., with unknown outcomes) at baseline, resulting in what appeared to be a low overall return to care by reengagement programs. Reengagement efforts targeted at those truly disengaged showed high return rates. Improving health systems to facilitate transfers and optimize information systems could allow for more targeted reengagement interventions in the future.

In our systemic review, we found that the definition of LTFU varied markedly from any missed visit to no visit in 12 months or more but was most commonly characterized as missing clinic appointments. Harmonizing definitions of LTFU will be beneficial to this field of research or program evaluation. A standardized universal definition(s), such as the one defined as more than 180 days since the last clinical visit [39], could enable us to conduct more reliable systematic review and comparable program evaluation worldwide.

Understanding the optimum intensity and time to initiate reengagement activities remains unclear and is a possible area for future inquiry. Reengagement program effectiveness is highly sensitive to the fact that returns among those defined as LTFU is subject to site-to-site variability in the definition of LTFU. Programs with a low threshold for labeling patients as lost (including many who are simply a little late and likely to return) may falsely appear to have highly effective reengagement programs. It may, however, be inefficient to initiate extensive early tracing processes for those who will return quickly, but at the same time, early tracing interventions could reduce the duration of gaps in care, improving treatment outcomes and reducing community HIV transmission. Across studies, LTFU definitions that triggered reengagement activities varied markedly with tracing occurring within a few days of a missed visit in some studies and more than 1 year later in others. Analyses stratified by LTFU definition, however, did not show any clear benefit of one LTFU definition over another (possibly due to the marked heterogeneity of several other study features), and there was no head-to-head comparisons of early versus late reengagement efforts. Identifying whom to trace at which time point after a missed visit could improve the efficiency and impact of reengagement interventions and could be critical next steps in designing optimum strategies to improve reengagement in care.

In addition to identifying and contacting PLWH who have disengaged from care, supporting reengagement after the return is a further attribute of reengagement interventions that could improve long-term outcomes; i.e., those who disengage may have ongoing barriers to care, which put them at risk for repeated disengagement episodes and poor treatment outcomes. Data from Zambia suggest that among PLWH established on ART who disengage, those with repeated disengagement episodes have higher mortality risk [40]. In this review, there were insufficient studies reporting on retention or viral suppression after return to draw firm conclusions on long-term intervention effects, and only one study reported specifically addressing barriers to care after return. The importance of providing additional reengagement support is gaining importance globally. Medicens Sans Frontiers in South Africa established a “Welcome Service” approach in 2018 aimed at facilitating successful reengagement by reorganizing the triage to streamline services and conducting training to address negative staff attitudes and authoritarian behaviors [41]. However, changing staff attitude to provide specialized services for those patients who returned was a challenge, highlighting that successful reengagement strategies will need to address both individual and health system barriers (including staff attitude) to engagement in care [42].

### Limitations

We found marked heterogeneity of intervention characteristics, settings, and outcome reporting; this remained unexplained by subgroup analysis and made pooled estimates less reliable. The majority of included studies had no comparison arm, and, therefore, there were few studies that contributed to estimates of effectiveness. Two included studies from New York [38,43] and two included studies from Malawi [44,45] had the possible risk of overlapping populations as they had some overlapping time of enrollment; we were not able to remove or check the effect of these overlaps in our analysis.

## Conclusions

Despite limitations and substantial heterogeneity, our systematic review and meta-analysis of the few studies that had control conditions revealed that reengagement interventions may increase return among PLWH who have disengaged from care and identified several gaps in reengagement services that should be addressed to improve efficiency and effectiveness. First, health systems should consider investing in information systems to improve the recording of deaths and transfers and better characterize LTFU. Second, research exploring varying reengagement strategies for different patient profiles and gaps in care could aid in the development of more targeted and efficient interventions in settings where resource limitations influence the breadth and intensity of available reengagement services. And, lastly, in addition to identifying the best combination of strategies to encourage return, strategies to retain PLWH in care after return should be explored to improve long-term outcomes after return.

## Supporting information

S1 Text Appendix A. Search Strategies. Appendix B. Risk of bias of included studies. Appendix C. Detailed PRECIS-2 scores. Appendix D. Detailed included studies. Appendix E. Supplementary figures and tables. Appendix F. List of included studies. Appendix G. PRISMA. Appendix H. Publication bias.
(DOCX)

## Abbreviations:

ART: antiretroviral therapy
CI: confidence interval
CROI: Conference on Retroviruses and Opportunistic Infections
EMR: electronic medical record
HIC: high-income country
IAC: International AIDS Conference
ICTRP: International Clinical Trial Registries Platform
LMIC: low- and middle-income country
LTFU: lost to follow-up
MSM: men who have sex with men
PLWH: people living with HIV
PWID: people who inject drugs
RCT: randomized controlled trial
RD: risk difference
RR: risk ratio
SOC: standard of care

## References

[1] Eshun-Wilson I, Rohwer A, Hendricks L, Oliver S, Garner P. Being HIV positive and staying on antiretroviral therapy in Africa: A qualitative systematic review and theoretical model. PLoS ONE. 2019;14(1):e0210408. doi: 10.1371/journal.pone.0210408 30629648 PMC6328200

[2] Sikazwe I, Eshun-Wilson I, Sikombe K, Czaicki N, Somwe P, Mody A, et al. Retention and viral suppression in a cohort of HIV patients on antiretroviral therapy in Zambia: Regionally representative estimates using a multistage-sampling-based approach. PLoS Med. 2019;16(5):e1002811. Epub 2019/06/01. doi: 10.1371/journal.pmed.1002811 ; PubMed Central PMCID: PMC6544202. 31150380 PMC6544202

[3] Geng EH, Odeny TA, Lyamuya R, Nakiwogga-Muwanga A, Diero L, Bwana M, et al. Retention in Care and Patient-Reported Reasons for Undocumented Transfer or Stopping Care Among HIV-Infected Patients on Antiretroviral Therapy in Eastern Africa: Application of a Sampling-Based Approach. Clin Infect Dis. 2016;62(7):935–44. doi: 10.1093/cid/civ1004 ; PubMed Central PMCID: PMC4787603. 26679625 PMC4787603

[4] Ware NC, Wyatt MA, Geng EH, Kaaya SF, Agbaji OO, Muyindike WR, et al. Toward an understanding of disengagement from HIV treatment and care in sub-Saharan Africa: a qualitative study. PLoS Med. 2013;10(1):e1001369; discussion e. Epub 2013/01/24. doi: 10.1371/journal.pmed.1001369 ; PubMed Central PMCID: PMC3541407. 23341753 PMC3541407

[5] Quinn KG, Reed SJ, Dickson-Gomez J, Kelly JA. An Exploration of Syndemic Factors That Influence Engagement in HIV Care Among Black Men. Qual Health Res. 2018;28(7):1077–87. Epub 2018/02/27. doi: 10.1177/1049732318759529 ; PubMed Central PMCID: PMC5962406. 29478406 PMC5962406

[6] Cargill VA. Linkage, engagement, and retention in HIV care among vulnerable populations: “Im sick and tired of being sick and tired”. Top Antivir Med. 2013;21(4):133–7. . 24225079 PMC6148843

[7] Walcott M, Kempf M-C, Merlin JS, Turan JM. Structural community factors and sub-optimal engagement in HIV care among low-income women in the Deep South of the USA. Cult Health Sex. 2016;18(6):682–94. doi: 10.1080/13691058.2015.1110255 26670722 PMC6047529

[8] Mody A, Eshun-Wilson I, Sikombe K, Schwartz SR, Beres LK, Simbeza S, et al. Longitudinal engagement trajectories and risk of death among new ART starters in Zambia: A group-based multi-trajectory analysis. PLoS Med. 2019;16(10):e1002959. Epub 2019/10/30. doi: 10.1371/journal.pmed.1002959 . 31661487 PMC6818762

[9] Lee H, Hogan JW, Genberg BL, Wu XK, Musick BS, Mwangi A, et al. A state transition framework for patient-level modeling of engagement and retention in HIV care using longitudinal cohort data. Stat Med. 2018;37 (2):302–19. doi: 10.1002/sim.7502 ; PubMed Central PMCID: PMC5735035. 29164648 PMC5735035

[10] Magnus M, Herwehe J, Gruber D, Wilbright W, Shepard E, Abrams A, et al. Improved HIV-related outcomes associated with implementation of a novel public health information exchange. Int J Med Inform. 2012;81(10):e30–8. Epub 2012/08/14. doi: 10.1016/j.ijmedinf.2012.06.005 . 22883431

[11] Bradford JB, Coleman S, Cunningham W. HIV System Navigation: an emerging model to improve HIV care access. AIDS Patient Care STDS. 2007;21(Suppl 1):S49–58. Epub 2007/08/01. doi: 10.1089/apc.2007.9987 . 17563290

[12] Bean MC, Scott L, Kilby JM, Richey LE. Use of an Outreach Coordinator to Reengage and Retain Patients with HIV in Care. AIDS Patient Care STDS. 2017;31(5):222–6. Epub 2017/05/11. doi: 10.1089/apc.2016.0318 . 28488904

[13] Covidence. Covidence systematic review software, Veritas Health Innovation, Melbourne, Australia. Available from: www.covidence.org. [cited 2020 Mar 25].

[14] Higgins J, Green S. Cochrane Handbook for Systematic Reviews of Interventions Version 5.1.0 [updated March 2011]. The Cochrane Collaboration. Available from: http://handbook.cochrane.org. [cited 2020 Mar 25].

[15] Wells G, Shea B, O’Connell D, Peterson J, Welch V, Losos M, et al. The Newcastle–Ottawa Scale (NOS) for Assessing the Quality of Non-Randomized Studies in Meta-Analysis. Available from: http://www.ohri.ca/programs/clinical_epidemiology/oxford.asp. [cited 2020 Mar 25].

[16] Gile KJ, Johnston LG, Salganik MJ. Diagnostics for Respondent-driven Sampling. J R Stat Soc Ser A Stat Soc. 2015;178(1):241–69. Epub 2015/01/01. doi: 10.1111/rssa.12059 ; PubMed Central PMCID: PMC4877136. 27226702 PMC4877136

[17] Guyatt GH, Oxman AD, Kunz R, Brozek J, Alonso-Coello P, Rind D, et al. GRADE guidelines 6. Rating the quality of evidence—imprecision. J Clin Epidemiol. 2011;64(12):1283–93. doi: 10.1016/j.jclinepi.2011.01.012 21839614

[18] Guyatt GH, Oxman AD, Kunz R, Woodcock J, Brozek J, Helfand M, et al. GRADE guidelines: 8. Rating the quality of evidence—indirectness. J Clin Epidemiol. 2011;64(12):1303–10. doi: 10.1016/j.jclinepi.2011.04.014 21802903

[19] Guyatt GH, Oxman AD, Kunz R, Woodcock J, Brozek J, Helfand M, et al. GRADE guidelines: 7. Rating the quality of evidence—inconsistency. J Clin Epidemiol. 2011;64(12):1294–302. Epub 2011/08/02. doi: 10.1016/j.jclinepi.2011.03.017 . 21803546

[20] Guyatt GH, Oxman AD, Montori V, Vist G, Kunz R, Brozek J, et al. GRADE guidelines: 5. Rating the quality of evidence—publication bias. J Clin Epidemiol. 2011;64(12):1277–82. Epub 2011/08/02. doi: 10.1016/j.jclinepi.2011.01.011 . 21802904

[21] Guyatt GH, Oxman AD, Vist G, Kunz R, Brozek J, Alonso-Coello P, et al. GRADE guidelines: 4. Rating the quality of evidence—study limitations (risk of bias). J Clin Epidemiol. 2011;64(4):407–15. Epub 2011/01/21. doi: 10.1016/j.jclinepi.2010.07.017 . 21247734

[22] Newcombe RG. Two-sided confidence intervals for the single proportion: comparison of seven methods. Stat Med. 1998;17(8):857–72. Epub 1998/05/22. doi: 10.1002/(sici)1097-0258(19980430)17:8&lt;857::aid-sim777&gt;3.0.co;2-e/(sici)1097-0258(19980430)17:8<857::aid-sim777>3.0.co;2-e 9595616

[23] Sterne JAC, Sutton AJ, Ioannidis JPA, Terrin N, Jones DR, Lau J, et al. Recommendations for examining and interpreting funnel plot asymmetry in meta-analyses of randomised controlled trials. BMJ. 2011;343:d4002. doi: 10.1136/bmj.d4002 21784880

[24] Harbord RM, Egger M, Sterne JA. A modified test for small-study effects in meta-analyses of controlled trials with binary endpoints. Stat Med. 2006;25(20):3443–57. Epub 2005/12/14. doi: 10.1002/sim.2380 . 16345038

[25] Kicinski M. Publication Bias in Recent Meta-Analyses. PLoS ONE. 2013;8(11):e81823. doi: 10.1371/journal.pone.0081823 24363797 PMC3868709

[26] Nabaggala MS, Parkes-Ratanshi R, Kasirye R, Kiragga A, Castlenuovo B, Ochaka I, et al. Re-engagement in HIV care following a missed visit in rural Uganda. BMC Res Notes. 2018;11(1):762. Epub 2018/10/26. doi: 10.1186/s13104-018-3865-9 ; PubMed Central PMCID: PMC6202822. 30359290 PMC6202822

[27] Nakiwogga-Muwanga A, Musaazi J, Katabira E, Worodria W, Talisuna SA, Colebunders R. Patients who return to care after tracking remain at high risk of attrition: experience from a large HIV clinic, Uganda. Int J STD AIDS. 2015;26(1):42–7. Epub 2014/03/22. doi: 10.1177/0956462414529098 . 24648320

[28] Bove JM, Golden MR, Dhanireddy S, Harrington RD, Dombrowski JC. Outcomes of a Clinic-Based Surveillance-Informed Intervention to Relink Patients to HIV Care. J Acquir Immune Defic Syndr. 2015;70(3):262–8. Epub 2015/06/13. doi: 10.1097/QAI.0000000000000707 ; PubMed Central PMCID: PMC4607589. 26068720 PMC4607589

[29] Alizadeh F, Mfitumuhoza G, Stephens J, Habimaana C, Myles K, Baganizi M, et al. Identifying and Reengaging Patients Lost to Follow-Up in Rural Africa: The “Horizontal” Hospital-Based Approach in Uganda. Glob Health Sci Pract. 2019;7(1):103. doi: 10.9745/GHSP-D-18-00394 30926739 PMC6538125

[30] Beres LK, Mody A, Sikombe K, Nicholas LH, Schwartz S, Eshun-Wilson I, et al. The effect of tracer contact on return to care among adult, “lost to follow-up” patients living with HIV in Zambia: an instrumental variable analysis. J Int AIDS Soc. 2021;24:e25853. doi: 10.1002/jia2.25853 34921515 PMC8683971

[31] Keller J, Heine A, LeViere AF, Donovan J, Wilkin A, Sullivan K, et al. HIV patient retention: the implementation of a North Carolina clinic-based protocol. AIDS Care. 2017;29(5):627–31. Epub 2016/09/04. doi: 10.1080/09540121.2016.1226478 . 27590445

[32] Udeagu C, Huang J, Eason L, Pickett L. Health department-HIV clinic integration of data and human resources to re-engage out of care HIV-positive persons into clinical care in a New York City locale. AIDS Care. 2019;31(11):1420–6. Epub 2019/03/02. doi: 10.1080/09540121.2019.1587373 . 30821484

[33] Kunzweiler C, Kishore N, John B, Roosevelt K, Lewis S, Klevens RM, et al. Using HIV Surveillance and Clinic Data to Optimize Data to Care Efforts in Community Health Centers in Massachusetts: The Massachusetts Partnerships for Care Project. J Acquir Immune Defic Syndr. 2019;82(Suppl 1):S33–s41. Epub 2019/08/20. doi: 10.1097/QAI.0000000000002019 . 31425393

[34] Lubelchek RJ, Fritz ML, Finnegan KJ, Trick WE. Use of a Real-Time Alert System to Identify and Re-Engage Lost-to-Care HIV Patients. J Acquir Immune Defic Syndr. 2016;72(2):e52–5. Epub 2016/02/27. doi: 10.1097/QAI.0000000000000973 . 26918542

[35] Sharp J, Angert CD, McConnell T, Wortley P, Pennisi E, Roland L, et al. Health Information Exchange: A Novel Re-linkage Intervention in an Urban Health System. Open Forum Infect Dis. 2019;6(10):ofz402. Epub 2019/10/30. doi: 10.1093/ofid/ofz402 ; PubMed Central PMCID: PMC6785665. 31660364 PMC6785665

[36] Sitapati AM, Limneos J, Bonet-Vazquez M, Mar-Tang M, Qin H, Mathews WC. Retention: building a patient-centered medical home in HIV primary care through PUFF (Patients Unable to Follow-up Found). J Health Care Poor Underserved. 2012;23(3 Suppl):81–95. Epub 2012/08/17. doi: 10.1353/hpu.2012.0139 . 22864489

[37] Fox MP, Pascoe SJS, Huber AN, Murphy J, Phokojoe M, Gorgens M, et al. Effectiveness of interventions for unstable patients on antiretroviral therapy in South Africa: results of a cluster-randomised evaluation. Trop Med Int Health. 2018;23(12):1314–25. Epub 2018/10/04. doi: 10.1111/tmi.13152 . 30281882

[38] Udeagu CN, Shah S, Misra K, Sepkowitz KA, Braunstein SL. Where Are They Now? Assessing if Persons Returned to HIV Care Following Loss to Follow-Up by Public Health Case Workers Were Engaged in Care in Follow-Up Years. AIDS Patient Care STDS. 2018;32(5):181–90. Epub 2018/05/12. doi: 10.1089/apc.2018.0004 . 29750551

[39] Chi BH, Yiannoutsos CT, Westfall AO, Newman JE, Zhou J, Cesar C, et al. Universal Definition of Loss to Follow-Up in HIV Treatment Programs: A Statistical Analysis of 111 Facilities in Africa, Asia, and Latin America. PLoS Med. 2011;8(10):e1001111. doi: 10.1371/journal.pmed.1001111 22039357 PMC3201937

[40] Holmes CB, Sikazwe I, Sikombe K, Eshun-Wilson I, Czaicki N, Beres LK, et al. Estimated mortality on HIV treatment among active patients and patients lost to follow-up in 4 provinces of Zambia: Findings from a multistage sampling-based survey. PLoS Med. 2018;15(1):e1002489. doi: 10.1371/journal.pmed.1002489 ; PubMed Central PMCID: PMC5766235. 29329301 PMC5766235

[41] Arendse KD, Pfaff C, Makeleni-Leteze T, T. D, Keene CM, Mantangana N, et al. Addressing disengagement from HIV healthcare services in Khayelitsha, South Africa, through Médecins Sans Frontières’ Welcome Service approach: comprehensive clinical and patient centered care; Available from: https://theprogramme.ias2021.org/Abstract/Abstract/1662. [cited 2021 Dec 27]. 11th IAS Conference on HIV Science 2021.

[42] Nhemachena T, Cassidy T, Spath C, Keene C, Zokufa N, Arensdse KD, et al., editors. Between empathy and anger: healthcare workers’ perspectives on patient disengagement from antiretroviral treatment in Khayelitsha, Cape Town. Available from: https://theprogramme.ias2021.org/Abstract/Abstract/649 [cited 27 December 2021]. 11th IAS Conference on HIV Science 2021; Ther Ber. 10.1186/s12875-022-01957-8 PMC9878968 36698083

[43] Udeagu CC, Webster TR, Bocour A, Michel P, Shepard CW. Lost or just not following up: public health effort to re-engage HIV-infected persons lost to follow-up into HIV medical care. AIDS. 2013;27(14):2271–9. Epub 2013/05/15. doi: 10.1097/QAD.0b013e328362fdde . 23669157

[44] Ardura-Garcia C, Feldacker C, Tweya H, Chaweza T, Kalulu M, Phiri S, et al. Implementation and Operational Research: Early Tracing of Children Lost to Follow-Up From Antiretroviral Treatment: True Outcomes and Future Risks. J Acquir Immune Defic Syndr (1999). 2015;70(5):e160–e7. doi: 10.1097/QAI.0000000000000772 . 26218409 PMC4645964

[45] Tweya H, Gareta D, Chagwera F, Ben-Smith A, Mwenyemasi J, Chiputula F, et al. Early active follow-up of patients on antiretroviral therapy (ART) who are lost to follow-up: the “Back-to-Care” project in Lilongwe, Malawi. Trop Med Int Health. 2010;15(Suppl 1):82–9. Epub 2010/07/14. doi: 10.1111/j.1365-3156.2010.02509.x . 20586965

