# Supplementary material for: Interventions to reengage people living with HIV who are lost to follow-up from HIV treatment programs: A systematic review and meta-analysis
Source: PLoS Med. 2022 Mar 15;19(3):e1003940. doi: 10.1371/journal.pmed.1003940 (PMC8923443; doi:10.1371/journal.pmed.1003940)
Supplement: S1 Text — Appendix A. Search Strategies. Appendix B. Risk of bias of included studies. Appendix C. Detailed PRECIS-2 scores. Appendix D. Detailed included studies. Appendix E. Supplementary figures and tables. Appendix F. List of included studies. Appendix G. PRISMA. Appendix H. Publication bias. (DOCX) [file pmed.1003940.s001.docx]

## List of Appendixes

## Appendix A – Search Strategies

## Appendix B - Risk of bias of included studies

## Appendix C - Detailed PRECIS-2 scores

## Appendix D - Detailed included studies

## Appendix E - Supplementary Figures and Tables

## Appendix F – List of included studies

## Appendix G – PRISMA

## Appendix H – Publication Bias

## Appendix A – Search Strategies

**PubMed:**

("Lost to Follow-Up"[Mesh] OR “lost to follow-up”[tw] OR “loss to follow up”[tw] OR "Patient Dropouts"[Mesh] OR “patient dropout”[tw] OR “patient dropouts”[tw] OR “loss to care”[tw] OR "lost to care" [tw] OR out-of-care [tw] OR “out of care”[tw] OR missed clinic visits [tw] OR lost [tw] OR drop-out [tw] OR drop-outs[tw] OR “lost cohort”[all fields])

AND

("Retention in Care"[Mesh] OR retention*[tiab] OR retain*[tiab] OR engage*[tiab] OR “Continuity of Patient Care”[mh] OR continuity of care[tiab] OR continuum[tiab] OR “treatment cascade” [tiab] OR reengagement[tiab] OR “re-engagement”[tiab] OR link*[tiab] OR relink*[tiab] OR “return to care”[tiab])

AND

(HIV Infections[Mesh] OR HIV[Mesh] OR HIV[tiab] OR HIV/AIDS OR hiv-1[tiab] OR hiv1[tiab] OR human immunodeficiency virus[tiab] OR human immune deficiency virus[tiab] OR human immuno-deficiency virus[tiab] OR human immune-deficiency virus[tiab] OR ((human immun*) AND (deficiency virus[tiab])) OR acquired immunodeficiency syndromes[tiab] OR acquired immune deficiency syndrome[tiab] OR acquired immuno-deficiency syndrome[tiab] OR acquired immunedeficiency syndrome[tiab] OR ((acquired immun*) AND (deficiency syndrome[tiab])))

AND

(“randomized controlled trial”[pt] OR “controlled clinical trial”[pt] OR “randomized controlled trials” OR “random allocation”[Mesh] OR random*[tiab] OR trial*[tiab] OR “prospective studies”[Mesh] OR intervention*[tiab] OR Case-Control Studies[mh] OR case-control*[tiab] OR “Cross-Sectional Studies”[mh] OR cross-section*[tiab] OR "Observational Study" [pt] OR observational[tiab] OR “Cohort studies”[Mesh] OR “Program evaluation”[Mesh] OR non-random*[tiab] OR nonrandom*[tiab] OR “before and after”[tiab] OR “time series”[tiab] OR cohort*[tiab] OR intervention*[tiab] OR prospective*[tiab] OR cluster*[tiab] OR longitud*[tiab] OR systematic review*[ti] OR meta-analysis[ti] OR metaanalysis[ti] OR (systematic*[tiab] AND review*[tiab]) OR systemic review[tiab] OR systematic[sb] OR Meta-analysis[pt])

**Web of Science:**

("Lost to Follow-Up"[Mesh] OR “lost to follow-up” OR “loss to follow up” OR "Patient Dropouts"[Mesh] OR “patient dropout” OR “patient dropouts” OR “loss to care” OR "lost to care" OR out-of-care OR “out of care” OR missed clinic visits OR lost OR drop-out OR drop-outs OR “lost cohort”[all fields])

AND

("Retention in Care"[Mesh] OR retention* OR retain* OR engage* OR “Continuity of Patient Care”[mesh] OR continuity of care OR continuum OR “treatment cascade” OR reengagement OR “re-engagement” OR link* OR relink* OR “return to care”)

AND

(HIV Infections[Mesh] OR HIV[Mesh] OR HIV OR HIV/AIDS OR hiv-1 OR hiv1 OR human immunodeficiency virus OR human immune deficiency virus OR human immuno-deficiency virus OR human immune-deficiency virus OR ((human immun*) AND (deficiency virus)) OR acquired immunodeficiency syndromes OR acquired immune deficiency syndrome OR acquired immuno-deficiency syndrome OR acquired immunedeficiency syndrome OR ((acquired immun*) AND (deficiency syndrome)))

AND

(“randomized controlled trial” OR “controlled clinical trial” OR “randomized controlled trials” OR “random allocation”[Mesh] OR random* OR trial* OR “prospective studies”[Mesh] OR intervention* OR Case-Control Studies[mesh] OR case-control* OR “Cross-Sectional Studies”[mesh] OR cross-section* OR "Observational Study" OR observational OR “Cohort studies”[Mesh] OR “Program evaluation”[Mesh] OR non-random* OR nonrandom* OR “before and after” OR “time series” OR cohort* OR intervention* OR prospective* OR cluster* OR longitud* OR systematic review* OR meta-analysis OR metaanalysis OR (systematic* AND review*) OR systemic review OR systematic OR Meta-analysis)

**Embase** search in translation:

((('follow up'/exp OR 'lost to follow-up':ab,ti OR 'loss to follow up':ti,ab,kw OR 'patient dropouts' OR 'patient dropout':ti,ab,kw OR 'patient dropouts':ti,ab,kw OR 'loss to care':ti,ab,kw OR 'lost to care':ti,ab,kw OR 'out of care':ti,ab,kw OR missed) AND clinic AND visits:ti,ab,kw OR lost:ti,ab,kw OR 'drop out':ti,ab,kw OR 'drop outs':ti,ab,kw OR 'lost cohort':ab,ti) AND (('retention in care'/exp OR retention*:ab,ti OR retain*:ab,ti OR engage*:ab,ti OR 'patient care' OR continuity) AND of AND care:ab,ti OR continuum:ab,ti OR 'treatment cascade':ab,ti OR reengagement:ab,ti OR 're-engagement':ab,ti OR link*:ab,ti OR relink*:ab,ti OR 'return to care':ab,ti) AND (('human immunodeficiency virus infection' OR 'human immunodeficiency virus' OR 'human immunodeficiency virus 1') OR ((((((((('hiv 1':ab,ti OR hiv1:ab,ti OR human) AND immunodeficiency AND virus:ab,ti OR human) AND immune AND deficiency AND virus:ab,ti OR human) AND 'immuno deficiency' AND virus:ab,ti OR human) AND 'immune deficiency' AND virus:ab,ti OR (human AND immun* AND deficiency AND virus:ab,ti) OR acquired) AND immunodeficiency AND syndromes:ab,ti OR acquired) AND immune AND deficiency AND syndrome:ab,ti OR acquired) AND 'immuno deficiency' AND syndrome:ab,ti OR acquired) AND immunedeficiency AND syndrome:ab,ti OR (acquired AND immun* AND deficiency AND syndrome:ab,ti)))) AND ('clinical trial'/de OR 'cohort analysis'/de OR 'controlled clinical trial'/de OR 'controlled study'/de OR 'cross-sectional study'/de OR 'longitudinal study'/de OR 'major clinical study'/de OR 'multicenter study'/de OR 'observational study'/de OR 'prospective study'/de OR 'qualitative research'/de OR 'randomized controlled trial'/de OR 'retrospective study'/de OR 'systematic review'/de)

**Global index medicus (Regional databases)**

(tw:(HIV OR human immunodeficiency)) AND (tw:("lost to follow-up" OR "loss to follow up" OR "patient dropout" OR "patient dropouts" OR "loss to care" OR "lost to care" OR "out-of-care" OR "out of care" OR "missed clinic visits" OR "drop-out" OR "drop-outs" OR "lost cohort"))

**PsycINFO/ERIC/Sociological Abstracts**

("lost to follow-up" OR "loss to follow up" OR "patient dropout" OR "patient dropouts" OR "loss to care" OR "lost to care" OR out-of-care OR "out of care" OR "missed clinic visits" OR drop-out OR drop-outs OR "lost cohort")

AND ("Retention in Care" OR retention OR retain OR engage OR engagin OR "Continuity of Patient Care" OR "continuity of care" OR continuum OR "treatment cascade" OR reengagement OR "re-engagement" OR link OR linking OR relink OR relinking OR "return to care")

AND ("HIV Infections" OR HIV OR HIV OR hiv-1 OR hiv1 OR "human immunodeficiency virus" OR "human immune deficiency virus" OR "human immuno-deficiency virus" OR "human immune-deficiency virus" OR ((human immun*) AND (deficiency virus)) OR acquired immunodeficiency syndromes OR acquired immune deficiency syndrome OR acquired immuno-deficiency syndrome OR acquired immunedeficiency syndrome OR ((acquired immun*) AND (deficiency syndrome)))

**Cochrane CENTRAL**

HIV OR "Human immunodeficiency" in Title Abstract Keyword AND ("lost to follow-up" OR "loss to follow up" OR "patient dropout" OR "patient dropouts" OR "loss to care" OR "lost to care" OR "out-of-care" OR "out of care" OR "missed clinic visits" OR "drop-out" OR "drop-outs" OR "lost cohort") in Title Abstract Keyword - (Word variations have been searched

## Appendix B - Risk of bias of included studies

## a. RCTs

| Study | Outcome | Sequence Generation | Allocation Concealment | Blinding Participants / Personnel | Blinding Outcome Assessor | Attrition Bias | Selective Reporting | Other Bias | Overall ROB |
| --- | --- | --- | --- | --- | --- | --- | --- | --- | --- |
| Udeagu 2019 | Return to care | Unclear risk | Unclear risk | Unclear risk | Unclear risk | Attrition not relevant in this study | Only adjusted odds ratio reported with no details of how adjustment was made | Low risk | Some concerns |
| Villanueva 2019 | Return to care | Unclear risk | Unclear risk | Unclear risk | Unclear risk | Attrition not relevant in this study | Both primary and secondary outcomes were reported | Low risk | Some concerns |
| Villanueva 2019 | Viral suppression | Unclear risk | Unclear risk | Unclear risk | Unclear risk | Attrition not relevant in this study | All major outcomes were reported | Low risk | Some concerns |
| Fox 2018 | Return to care | Computer randomization was used to allocate one to  be an intervention site | Unclear risk | Unclear risk | Unclear risk | Attrition not relevant in this study | All major outcomes were reported | Low risk | Low risk |
| Fox 2018 | Retention in Care | Computer randomization was used to allocate one to  be an intervention site | Unclear risk | Unclear risk | Unclear risk | Attrition not relevant in this study | All three major outcomes were reported | Low risk | Low risk |
| Fox 2018 | Viral suppression | Computer randomization was used to allocate one to  be an intervention site | Unclear risk | Unclear risk | Unclear risk | Attrition not relevant in this study | All three major outcomes were reported | Low risk | Low risk |
| Bershetyn 2017 | Return to care | A random selection for tracing as an instrument variable | Unclear risk | Unclear risk | Unclear risk | Attrition not relevant in this study | Main outcome was reported. | Low risk | Low risk |
| Beres 2019 | Return to care | Using randomization to tracing efforts as an instrumental variable | Unclear risk | Unclear risk | Unclear risk | Attrition not relevant in this study | Main outcome was reported | Low risk | Low risk |
| Fanfair 2019 | Return to care | Unclear risk | Unclear risk | Unclear risk | Unclear risk | Attrition not relevant in this study | Main outcome was reported | Low risk | Some concerns |

## b. Cohort studies (Newcastle Ottawa ROB tool for comparative studies

| Study | Outcome | Selection1Exposed | Selection2NonExposed | Selection3AscertainExposure | Selection4OutcomeNotPresentAtStart | **SelectionStars** | Comparability | **ComparabilityStars** | Outcome1Assessment | Outcome2FollowupLongEnough | Outcome3AdequacyofFollowup | **OutcomeStars** | **Totalstars** | **OverallROB** |
| --- | --- | --- | --- | --- | --- | --- | --- | --- | --- | --- | --- | --- | --- | --- |
| Magnus 2012 | Return to care | Somewhat Representative(1*) | Same source as exposed(1*) | Secure Record(1*) | Yes(1*) | **** | Controlled for other factors(1*) | * | Record Linkage (1*) | Yes (1*) | Subjects Lost to Follow-Up; unlikely bias (1*) | *** | ******** | Good Quality |
| Magnus 2012 | Retention in Care | Somewhat Representative(1*) | Same source as exposed(1*) | Secure Record(1*) | Yes(1*) | **** | Controlled for other factors(1*) | * | Record Linkage (1*) | Yes (1*) | Retention is the outcome | *** | ******* | Good Quality |
| Bove 2015 | Return to care | Somewhat Representative (1*) | Different Source | Secure Record (1*) | Yes (1*) | *** | Controlled for age, sex +-marital status (1*) | * | Record Linkage (1*) | Yes (1*) | Return to care is the outcome | *** | ****** | Good quality |
| Bove 2015 | Viral suppression | Somewhat Representative (1*) | Different Source | Secure Record (1*) | No | ** | Controlled for age, sex +-marital status (1*) | * | Record Linkage (1*) | Yes (1*) | No Statement | ** | ***** | Fair Quality |
| Bove 2015 | Retention in Care | Somewhat Representative (1*) | Different Source | Secure Record (1*) | Yes (1*) | *** | Controlled for age, sex +-marital status (1*) | * | Record Linkage (1*) | Yes (1*) | Retention is the outcome | *** | ****** | Good Quality |
| Rebeiro 2017 | Return to care | Somewhat Representative (1*) | Different Source | Secure Record (1*) | No | ** | Controlled for age, sex +-marital status (1*) | * | Record Linkage (1*) | No Statement | Return to care is the outcome | * | **** | Poor Quality |
| Sharp 2019 | Return to care | Selected group | Same source as exposed (1*) | Secure Record(1*) | No | ** | Controlled for age, sex +-marital status (1*) | * | Record Linkage (1*) | Yes (1*) | Return to care is the outcome | *** | ***** | Fair Quality |
| Saafir-Callaway 2020 | Retention in Care | Somewhat Representative (1*) | Same source as exposed (1*) | Secure Record (1*) | Yes(1*) | **** | Controlled for age, sex +-marital status (1*) | * | Record Linkage (1*) | Yes (1*) | Retention is the outcome | *** | ******* | Good Quality |
| Saafir-Callaway 2020 | Retention in Care | Somewhat Representative (1*) | Same source as exposed (1*) | Secure Record (1*) | Yes(1*) | **** | Controlled for age, sex +-marital status (1*) |  | Record Linkage (1*) | Yes (1*) | Retention is the outcome | *** | ******* | Good Quality |
| Saafir-Callaway 2020 | Viral suppression | Somewhat Representative (1*) | Same source as exposed (1*) | Secure Record (1*) | Yes(1*) | **** | Controlled for age, sex +-marital status (1*) | * | Record Linkage (1*) | Yes (1*) | Follow-up <80% and no description | ** | ****** | Good Quality |
| Udeagu 2013 | Return to care | Somewhat Representative(1*) | N/A | Secure Record(1*) | Yes(1*) | *** | N/A | N/A | Record Linkage (1*) | Yes (1*) | Retention is the outcome | *** | ***** | N/A |
| Udeagu 2013 | Viral suppression | Somewhat Representative(1*) | N/A | Secure Record(1*) | Yes(1*) | *** | N/A | N/A | Record Linkage (1*) | No | Subjects Lost to Follow-Up; unlikely bias (1*) | ** | ***** | N/A |
| Udeagu 2013 | Retention in Care | Somewhat Representative(1*) | N/A | Secure Record(1*) | Yes(1*) | *** | N/A | N/A | Record Linkage (1*) | Yes (1*) | Retention is the outcome | *** | ***** | N/A |
| Udeagu 2018 | Return to care | Somewhat Representative(1*) | N/A | Secure Record(1*) | Yes(1*) | *** | N/A | N/A | Record Linkage (1*) | Yes (1*) | Subjects Lost to Follow-Up; unlikely bias (1*) | *** | ****** | N/A |
| Udeagu 2018 | Viral suppression | Somewhat Representative(1*) | N/A | Secure Record(1*) | Yes(1*) | *** | N/A | N/A | Record Linkage (1*) | Yes (1*) | Subjects Lost to Follow-Up; unlikely bias (1*) | *** | ****** | N/A |
| Udeagu 2018 | Retention in Care | Somewhat Representative(1*) | N/A | Secure Record(1*) | Yes(1*) | *** | N/A | N/A | Record Linkage (1*) | Yes (1*) | Subjects Lost to Follow-Up; unlikely bias (1*) | *** | ****** | N/A |
| Udeagu 2018 | Mortality | Somewhat Representative(1*) | N/A | Secure Record(1*) | Yes(1*) | *** | N/A | N/A | Record Linkage (1*) | Yes (1*) | Follow-up <80% and no description | ** | ***** | N/A |
| Nabaggala 2018 | Return to care | Somewhat Representative(1*) | N/A | Secure Record(1*) | Yes(1*) | *** | N/A | N/A | Record Linkage (1*) | Yes (1*) | Retention is the outcome | *** | ***** | N/A |
| Keller 2017 | Return to care | Somewhat Representative(1*) | N/A | Secure Record(1*) | Yes(1*) | *** | N/A | N/A | Record Linkage (1*) | Yes (1*) | Return to care is the outcome | *** | ***** | N/A |
| Deery 2014 | Return to care | Somewhat Representative(1*) | N/A | Secure Record(1*) | Yes(1*) | *** | N/A | N/A | Record Linkage (1*) | Yes (1*) | Return to care is the outcome | *** |  |  |
| Wohl 2016 | Return to care | Somewhat Representative(1*) | N/A | Secure Record(1*) | Yes(1*) | *** | N/A | N/A | Record Linkage (1*) | Yes (1*) | Subjects Lost to Follow-Up; unlikely bias (1*) | *** |  |  |
| Wohl 2016 | Viral suppression | Somewhat Representative(1*) | N/A | Secure Record(1*) | No | ** | N/A | N/A | Record Linkage (1*) | Yes (1*) | Follow-up <80% and no description | ** |  |  |
| Wohl 2016 | Retention in Care | Somewhat Representative(1*) | N/A | Secure Record(1*) | Yes(1*) | *** | N/A | N/A | Record Linkage (1*) | Yes (1*) | Retention is the outcome | *** |  |  |
| Alamo 2012 | Return to care | Somewhat Representative(1*) | N/A | Secure Record(1*) | Yes(1*) | *** | N/A | N/A | Record Linkage (1*) | Yes (1*) | Return to care is the outcome | *** |  |  |
| Ardura-Garci 2015 | Return to care | Somewhat Representative(1*) | N/A | Secure Record(1*) | No | ** | N/A | N/A | Record Linkage (1*) | No Statement | Return to care is the outcome | ** |  |  |
| Aebi-Popp 2016 | Return to care | Somewhat Representative (1*) | N/A | Secure Record (1*) | Yes (1*) | *** | N/A | N/A | Record Linkage (1*) | Yes (1*) | Subjects Lost to Follow-Up; unlikely bias (1*) | *** |  |  |
| Aebi-Popp 2016 | Viral suppression | Somewhat Representative (1*) | N/A | Secure Record (1*) | N/A | ** | N/A | N/A | Record Linkage (1*) | Yes (1*) | Subjects Lost to Follow-Up; unlikely bias (1*) | *** |  |  |
| Bean 2017 | Return to care | Somewhat Representative (1*) | N/A | Secure Record (1*) | Yes (1*) | *** | N/A | N/A | Record Linkage (1*) | Yes (1*) | Subjects Lost to Follow-Up; unlikely bias (1*) | *** |  |  |
| Bean 2017 | Retention in Care | Somewhat Representative (1*) | N/A | Secure Record (1*) | Yes (1*) | *** | N/A | N/A | Record Linkage (1*) | Yes (1*) | Subjects Lost to Follow-Up; unlikely bias (1*) | *** |  |  |
| Bupamba 2010 | Return to care | Truly Representative (1*) | N/A | Secure Record (1*) | Yes (1*) | *** | N/A | N/A | No Description | Yes (1*) | Subjects Lost to Follow-Up; unlikely bias (1*) | ** |  |  |
| Chikuse 2019 | Return to care | Truly Representative (1*) | N/A | Secure Record (1*) | No | ** | N/A | N/A | Record Linkage (1*) | Yes (1*) | Subjects Lost to Follow-Up; unlikely bias (1*) | *** |  |  |
| Donovan 2018 | Return to care | Somewhat Representative (1*) | N/A | Secure Record (1*) | Yes (1*) | *** | N/A | N/A | Record Linkage (1*) | Yes (1*) | Subjects Lost to Follow-Up; unlikely bias (1*) | *** |  |  |
| Donovan 2018 | Viral suppression | Truly Representative (1*) | N/A | Secure Record (1*) | Yes (1*) | *** | N/A | N/A | Record Linkage (1*) | Yes (1*) | No Statement | ** |  |  |
| Donovan 2018 | Retention in Care | Truly Representative (1*) | N/A | Secure Record (1*) | Yes (1*) | *** | N/A | N/A | Record Linkage (1*) | Yes (1*) | Subjects Lost to Follow-Up; unlikely bias (1*) | *** |  |  |
| Dufour 2018 | Return to care | Somewhat Representative (1*) | N/A | Secure Record (1*) | Yes (1*) | *** | N/A | N/A | Self-Report | No Statement | Return to care is the outcome | * |  |  |
| Edwards 2019 | Return to care | Truly Representative (1*) | N/A | Secure Record (1*) | Yes (1*) | *** | N/A | N/A | Record Linkage (1*) | Yes (1*) | Subjects Lost to Follow-Up; unlikely bias (1*) | *** |  |  |
| Edwards 2019 | Retention in Care | Truly Representative (1*) | N/A | Secure Record (1*) | Yes (1*) | *** | N/A | N/A | Record Linkage (1*) | No | Subjects Lost to Follow-Up; unlikely bias (1*) | ** |  |  |
| Healey 2018 | Return to care | Somewhat Representative (1*) | N/A | Secure Record (1*) | Yes (1*) | *** | N/A | N/A | No Description | No Statement | Subjects Lost to Follow-Up; unlikely bias (1*) | * |  |  |
| Lubelcheck 2016 | Return to care | Truly Representative (1*) | N/A | Secure Record (1*) | Yes (1*) | *** | N/A | N/A | Record Linkage (1*) | Yes (1*) | Complete Follow-Up (1*) | *** |  |  |
| McMahon 2015 | Return to care | Somewhat Representative (1*) | N/A | No Description | Yes (1*) | ** | N/A | N/A | Record Linkage (1*) | No Statement | Subjects Lost to Follow-Up; unlikely bias (1*) | ** |  |  |
| Nakiwogga-Muwanga 2015 | Return to care | Truly Representative (1*) | Same source as exposed (1*) | Secure Record (1*) | No | *** | N/A | N/A | Record Linkage (1*) | Yes (1*) | Subjects Lost to Follow-Up; unlikely bias (1*) | *** |  |  |
| Nakiwogga-Muwanga 2015 | Retention in Care | Truly Representative (1*) | Same source as exposed (1*) | Secure Record (1*) | Yes (1*) | **** | N/A | N/A | Record Linkage (1*) | Yes (1*) | Subjects Lost to Follow-Up; unlikely bias (1*) | *** |  |  |
| Nakiwogga-Muwanga 2015 | Mortality | Truly Representative (1*) | Same source as exposed (1*) | Secure Record (1*) | Yes (1*) | **** | N/A | N/A | Record Linkage (1*) | Yes (1*) | Subjects Lost to Follow-Up; unlikely bias (1*) | *** |  |  |
| Saafir-Callaway 2015 | Return to care | Truly Representative (1*) | N/A | No Description | Yes (1*) | ** | N/A | N/A | Record Linkage (1*) | No | Return to care is the outcome | * |  |  |
| Saafir-Callaway 2015 | Viral suppression | Truly Representative (1*) | N/A | No Description | No | * | N/A | N/A | Record Linkage (1*) | Yes (1*) | No Statement | ** |  |  |
| Saafir-Callaway 2015 | Retention in Care | Truly Representative (1*) | N/A | No Description | Yes (1*) | ** | N/A | N/A | Record Linkage (1*) | Yes (1*) | Retention is the outcome | *** |  |  |
| Sitapati 2012 | Return to care | Truly Representative (1*) | N/A | Secure Record (1*) | Yes (1*) | *** | N/A | N/A | Record Linkage (1*) | Yes (1*) | Subjects Lost to Follow-Up; unlikely bias (1*) | *** |  |  |
| Tesoriero 2017 | Return to care | Truly Representative (1*) | N/A | Secure Record (1*) | Yes (1*) | *** | N/A | N/A | Record Linkage (1*) | Yes (1*) | Subjects Lost to Follow-Up; unlikely bias (1*) | *** |  |  |
| Tesoriero 2017 | Retention in Care | Truly Representative (1*) | N/A | Secure Record (1*) | Yes (1*) | *** | N/A | N/A | Record Linkage (1*) | Yes (1*) | Subjects Lost to Follow-Up; unlikely bias (1*) | *** |  |  |
| Tweya 2010 | Return to care | Truly Representative (1*) | N/A | Secure Record (1*) | Yes (1*) | *** | N/A | N/A | Record Linkage (1*) | Yes (1*) | Subjects Lost to Follow-Up; unlikely bias (1*) | *** |  |  |
| Kunzweiler 2019 | Return to care | Somewhat Representative(1*) | N/A | Secure Record(1*) | Yes(1*) | *** | N/A | N/A | Record Linkage (1*) | Yes (1*) | Subjects Lost to Follow-Up; unlikely bias (1*) | *** |  |  |
| Kunzweiler 2019 | Retention in Care | Somewhat Representative(1*) | N/A | Secure Record(1*) | Yes(1*) | *** | N/A | N/A | Record Linkage (1*) | Yes (1*) | Subjects Lost to Follow-Up; unlikely bias (1*) | *** |  |  |
| Kunzweiler 2019 | Viral suppression | Somewhat Representative(1*) | N/A | Secure Record(1*) | Yes(1*) | *** | N/A | N/A | Record Linkage (1*) | Yes (1*) | Subjects Lost to Follow-Up; unlikely bias (1*) | *** |  |  |
| Fernández-Luis 2019 | Return to care | Somewhat Representative (1*) | N/A | Structured interview (1*) | Yes (1*) | *** | N/A | N/A | Record Linkage (1*) | Yes (1*) | Return to care is the outcome | *** |  |  |
| Alizadeh 2019 | Return to care | Somewhat Representative (1*) | N/A | Secure Record(1*) | Yes (1*) | *** | N/A | N/A | Record Linkage (1*) | Yes (1*) | Return to care is the outcome | *** |  |  |
| Alizadeh 2019 | Retention in Care | Somewhat Representative (1*) | N/A | Secure Record(1*) | Yes (1*) | *** | N/A | N/A | Record Linkage (1*) | Yes (1*) | Retention is the outcome | *** |  |  |
| Naidoo 2019 | Return to care | Somewhat Representative(1*) | N/A | Structured interview (1*) | Yes(1*) | *** | N/A | N/A | Record Linkage (1*) | No | Return to care is the outcome | ** |  |  |
| Chang 2019 | Return to care | Somewhat Representative(1*) | N/A | Secure Record(1*) | Yes(1*) | *** | N/A | N/A | Record Linkage (1*) | Yes (1*) | Return to care is the outcome | ** |  |  |

##

## Appendix C - Detailed PRECIS-2 scores

| **Study** | **Eligibility** | | **Recruitment / Cohort selection** | | **Setting** | | **Organization** | | **Flexibility: delivery** | | **Flexibility: adherence** | | **Follow-up** | | **Primary Outcome** | | **Primary Analysis** | |
| --- | --- | --- | --- | --- | --- | --- | --- | --- | --- | --- | --- | --- | --- | --- | --- | --- | --- | --- |
|  | Score | Justification | Score | Justification | Score | Justification | Score | Justification | Score | Justification | Score | Justification | Score | Justification | Score | Justification | Score | Justification |
| Beres 2019 | 5 | No exclusions | 5 | no recruitment | 5 | Routine care in Zambia | 5 | CIDRZ - NGO supported but in Zambia nearly all facilities are NGO supported | 4 | Done with usual care cadres, managed differently, but purpose was outcome ascertainment | 5 | Wide berth for real world tracing considerations | 5 | No activities undertaken for measurement that could influence behavior | 5 | Re-engagement relevant to patients | 5 | Intention to treat analysis |
| Bershetyn 2015 | 5 | No exclusions | 5 | A random subset of lost patients was selected for tracing. | 5 | 14 clinic sites in Eastern Africa located in 5 geographical settings | 4 | Clinics in the IEDEA network; potentially sights with better resources than others | 5 | Real world approach to tracing patients with all the flexibility required | 5 | Wide berth for real world tracing considerations | 5 | No activities undertaken for measurement that could influence behavior | 5 | Re-engagement relevant to patients | 5 | Intention to treat analysis |
| Bove 2015 | 5 | Approximates eligbility criteria for tracing in real world setting | 4 | Used clinic data to identify disengaged and incorporated health department HIV surveillance data to refine the list | 4 | Large public health clinic with significant support from public health department for identifying LTFU | 4 | A linkage specialist (LS) was employed to conduct outreach, not all systems may have the funds to employ additional personnel | 5 | Real world approach to tracing patients with all the flexibility required | 5 | Wide berth for real world tracing considerations | 5 | No activities undertaken for measurement that could influence behavior | 5 | Re-engagement relevant to patients | 5 | Intention to treat analysis |
| Fanfair 2019 | 4 | Broadly applicable, but included those LTFU 6 months (+1 mo lag) and not those out of care for longer periods | 5 | Intervention delivery appears to be delivered uniformly across eligible participants until sample size reached | 5 | Multi-site including public health and private clinics | 2 | Additional public health staff required to implement the intervention | 5 | A bit difficult to tell based on the poster, but from what is there not very prescriptive | 5 | End users had full flexibility in how they engaged with the intervention. | 5 | Based on EMR/lab records | 5 | Re-engagement & VS both highly relevant to patient | 5 | From what is there seems like all data used, but hard to read/and limited info |
| Fox 2018 | 4 | Largely applicable, however pregnant women and children excluded, as well as those not in the catchment area, though tracing interventions for those patients are likely to have feasiblity issues | 5 | CRT design with no patient contact, everyone meeting eligibility criteria in time period was included (waiver of consent was sought) | 4 | Multi-site public clinics; only high volume clinics selected (though mix of urban/rural) and presumably intervention would be applied in high and low volume settings. | 4 | Clinic staff delivered intervention; received minimal training, no feedback on quality or outcomes of interventions | 4 | Guidelines in place as to how the interventions should be delivered, but this was the nature of the interventions themselves | 5 | Nothing required of patient | 5 | EMR, patient records used | 5 | Return to care by 3 months relevant to those with prolonged missed visits | 4 | Retention at 12-months is a more distal outcome for patients but likely driven by the early nature of tracing |
| Magnus 2012 | 5 | Pragmatic among intervention group; control group retrospectively identified | 5 | Through EMR data and a rolled out alert system (La Phie) | 5 | Multi-clinic, where intervention being implemented | 4 | Some training for providers and providers engaged in peer training, but largely enacted as the new standard of care | 5 | Although providers received clinical guidance for how to manage the specific case, they had flexibility in how they ultimately managed and the person was reflagged if no action taken | 5 | Nothing required of patient | 5 | EMR | 5 | CD4<200, VL, ART all directly relevant to patients | Can't assess | Authors consider this a case-cohort study, but looks to actually (based on sampling) be a study using historical controls. Problematic in terms of comparator but implementation was pragmatic. |
| Rebeiro 2017 | 4 | Eligibility for analyses among those with known outcomes/gaps not everyone lost to care if outcome unknown, though sensitivity analyses were conducted. All were eligible for the actual tracing intervention. | 5 | Standard of care applied in these settings; everyone was found and EMR data used | 5 | Multi-site, SOC | 5 | Evaluation of the updated SOC | 5 | Tracing was routine, underspecified in paper, but does not seem to be overly rigid | 5 | Wide berth for real world tracing considerations | 5 | EMR data | 5 | Return to care over time | 3 | Return to care by tracing status; not ITT |
| Udeagu 2019 | 5 | Everyone determined to be OOC, though by definition of the intervention, this definition varied by comparison group in this observational design | 5 | Everyone eligible | 5 | Multi-site (some implementing enhanced intervention, others not) | 4 | Reported to use existing team members; though the determination of who was OOC was more intensive and took greater resources | 4 | Repeated attempt at delivery if unsuccessful, but contacting the patient was the core of the intervention so understandable why | 5 | Wide berth for real world tracing considerations | 5 | EMR | 5 | Re-engagement in care | 4 | All included, but those receiving enhanced efforts by definition of intervention identified differently |
| Villanueva 2019 | 3 | Excluded those on ART for <12 months; and those out of care for <6 months | 5 | Identified and randomized based on EMR status | 5 | Multi-site | 3 | Required hiring of DIS specialists with training | 5 | DIS specialists identify barriers and solutions with patients | 5 | Wide berth for real world tracing considerations | 5 | EMR | 5 | Re-engagement in care within 90 days | 5 | Everyone included |
| Sharp 2019 | 5 | Patients reporting in care excluded as not target population; patients admitted to hospital also excluded as they received a more intensive intervention | 4 | HIE alerts sent for everyone, but social workers only there daytime weekday hours so not everyone was recruited and there may be differences in those attending night and day, but pragmatic in terms of implementation and staffing constraints; given a 4 as children excluded. | 3 | Grady Health System is the largest HIV provider in Georgia, but not representative of health systems | 4 | 2 social workers added to implement intervention | 5 | Hours constrained to typical business hours, flexibility in case management approach to identify barriers and address them | 5 | Wide berth for real world tracing considerations | 5 | EMR | 5 | Linkage to care; viral suppression | 3 | Everyone included - those who did not receive (but had an alert) were treated as the controls in this observational study; not an ITT as the treatment was what drove the comparator arm |

## Appendix D - Detailed included studies

| **First author, year** | **Methods** | **Participants** | **Interventions** | **Outcomes** |
| --- | --- | --- | --- | --- |
| **Alamo 2012** | **Design:** Cohort  **Participants in the Cohort**: HIV+ patients in Kampala, Uganda  **Unit of randomization/study**: Patients  **Duration:** 31 May 2001 to 31 May 2010 | **City/State/Country:** Kampala, Uganda  **Setting:** Conducted in Kampala, Uganda, at the Reach Out Mbuya HIV/AIDS Initiative  **Inclusion criteria:** HIV+ patients 18 years of age or older. Lost to follow-up (90 days late or later for scheduled clinic appointment) between 5/31/2001 and 5/31/2010.  **Participants at baseline:** 579 patients sampled for tracing from 2933 lost to follow-up. 481 found alive and successfully contacted. Of those 481: 40.3% male.  **Age at baseline**: 9.6% were 18-24 years of age, 40.3% were 25-34 years old, 36.8% were 35-44 years old, 13.3% were 45 or older.  **Race/ethnicity at baseline**: Not given.  **Median viral load at last known lab:** Not given.  **CD4 levels:** 11.6% CD4 less than 100 cells per µL, 19.5% CD4 level between 100 and 250 cells per µL, 68.8% CD4 levels greater than 250 cells per µL. | **Intervention Description:** Community-based care, defined catchment area around clinic, task shifting, pre-ART counseling sessions, family-based care, provision of free services and financial support. with additional tracing component using the electronic health records. Trained peer tracers would attempt to contact patients every 1-4 weeks.  through a home visit or the same day as the missed appointment to encourage engagement in care.  **Control:** Not applicable | **Primary:** Returned to care. Defined as successfully traced and resumed receipt of care.  **Secondary:** Unable to contact; transferred clinic; median time from contact to return to care  **Assessed at:** the median time from LTFU to return into care was 911 days (2.5 years). |
| **Ardura-Garcia 2015** | **Design:** Cohort  **Participants in the Cohort**: HIV+ patients in urban Malawi  **Unit of randomization/study**: Patients  **Duration:** April 2006 to December 2010 | **City/State/Country:** Lilongwe, Malawi  **Setting:** Two HIV/ART clinics integrated inside hospitals in Lilongwe, Malawi.  **Inclusion criteria:** HIV+ children 15 or younger at ART initiation who missed ART dispensing visit by three weeks or more.  **Exclusion criteria:** Transferred-in patients were excluded.  **Participants at baseline:** 985 patients, of whom 251 HIV+ attendees identified as out-of-care. 47.0% male.  **Age at baseline**: Median age: 84 months (IQR: 38-132 months).  **Race/ethnicity at baseline**: Not given.  **Median viral load at last known lab:** Not given.  **Median CD4 at last known lab:** 286 cells per µL (IQR: 132-510). | **Intervention Description:** Team of three field tracers and a receptionist. Create a list every week of patients lost to follow-up (missed ART dispensing appointment by 3 weeks or more). Uses clinic records to verify lost to follow-up. Lost to follow-up patients are contacted 3 times by phone to encourage to return to care. If unsuccessful, up to 3 home visit attempts are made.  **Control:** Not applicable. | **Primary:** Returned to care. Defined as returning to one of the clinics for an appointment.  **Secondary:** Time to return to care after contact  **Assessed at:** 84 days (median time between last visit and first tracing attempt) |
| **Bean 2017** | **Design:** Cohort  **Participants in the Cohort**: out-of-care patients  **Unit of randomization/study**: Patients  **Duration:** 1 October 2014 until 31 December 2015 | **City/State/Country:** South Carolina, USA  **Setting:** The Medical University of South Carolina’s HIV clinic. Funded by Health Resources and Services Administration.  **Inclusion criteria:** Attended at least one clinic visit at The Medical University of South Carolina from 2009 to 2014. Not retained in care in for 12 months.  **Participants at baseline:** 233 out-of-care patients. Mean age: 42y, 77% Male  Mean CD4 count: 446, 72% African American **Age at baseline**: Mean age: 44 years (overall). 42 years (out-of-care population).**Median viral load at last known lab:** Not given. | **Intervention Description:** Coordinators investigated eligible patients to determine if deceased, incarcerated, moved, or out of care. Out of care patients were first called to re-engage. If unable to be contacted, a letter was written. If still unable to contact the patient after a call and letter, home visits were made to try to encourage patients to reengage in care.  **Control:** Not applicable . | **Primary:** Re-engaged in care. Defined as having a clinic visit in 2015.  **Secondary:** Retained in care. Defined as having two or more clinic visits 90 days or more apart.  Transferred care; deceased; incarcerated; responded to phone call; responded to letter; responded to home visit  **Assessed at:** 12 months (end of 2015) |
| **Beres 2019** | **Design:** RCT  **Participants in the Cohort**: HIV+ patients in Zambia  **Unit of randomization/study**: Patients  **Duration:** 1 August 2013 until 31 July 2015 | **City/State/Country:** Zambia  **Setting:** 71 clinics across 4 provinces in Zambia in Sub-Saharan Africa: Lusaka, Southern, Eastern, and Western provinces.  **Inclusion criteria:** HIV+ patients attending one of the 71 participating clinics. At least one study visit from 1 August 2013 to 31 July 2015. Having 90 days or more between most recent visit, unknown status since last clinic visit. Patients needed an electronic medical record (2015).  **Participants at baseline:**  33,533 not randomized to tracing: 39.5% male, 10.9% 18-24, 37.3% 25-34, 33.4% 35-44, 18.4% 45+  4,380 randomized for tracing: 39.6% male, 10.9% 18-24, 36.4% 25-34, 33.4% 35-44, 19.3% 45+**Race/ethnicity at baseline**: Not given. | **Intervention Description:** Patients out-of-care identified using electronic health records, and random sample selected for intervention. Trained peer educator traced selected patients, attempting to contact via home visits and phone calls. If contact was successful, tracers encouraged return to care, and could escort or meet patients at their visits.  **Control:** Not tracing | **Primary:** Returned to care, defined as having a study visit within 2 years after date of loss  **Secondary:** median time from loss to follow-up to tracer contact  **Assessed at:** 2 years (primary) |
| **Bershetyn 2017** | **Design:** Cohort  **Participants in the Cohort**: HIV/AIDS patients who attended 1 of 14 clinic in East Africa.  **Unit of randomization/study**: Patient  **Duration:** Not reported | **City/State/Country:** Uganda, Kenya, and Tanzania.  **Setting:** 14 clinic sites around Mbarara, Uganda; Kampala, Uganda; Morogoro, Tanzania; Kisumu, Kenya; and Eldoret, Kenya.  **Inclusion criteria:** Attending one of 14 participating clinics in Kenya, Uganda or Tanzania. Attended clinic within the last 2.5 years prior to the data of sampling. Considered lost to follow-up, defined as greater than 90 days late for last scheduled appointment.  From eligible patients, a random subset was chosen for inclusion.  **Participants at baseline:**  4790 not randomly sample for tracing: eligible patients: 34.6% Male, Median age: 34 years (IQR 28-41), Median CD4 at ART initiation: 138 (IQR 57-222)  991 randomly sampled for tracing: 38.0% Male, Median age: 34 years (IQR: (28-41. Median CD4 at ART initiation: 136 (IQR 60-225) | **Intervention Description:** Patient tracing by community health workers at the 14 clinic sites. Tracers were given lists of participants to follow-up with, and visited patients at their homes. Contacted patients were interviewed with 3-5 scripted questions to determine current care levels, reasons for leaving clinic, and encouraging returning to care.  **Control:** Patients in the control group were those identified as lost to follow-up, but were not traced or contacted by clinic workers for returning to care. | **Primary:** Return to care after loss to follow-up from HIV clinic.  **Assessed at:** One year after sampling. |
| **Bove 2015** | **Design:** Cohort  **Participants in the Cohort**: HIV Patients.  **Unit of randomization/study**: Patients  **Duration:** Intervention: 1 November 2012 to 1 November 2013 (observation period, but the intervention still ongoing at time of publication of Bove study) | **City/State/Country:** Seattle, Washington, USA  **Setting:** The Madison Clinic, part of the Habrborview Medical Center. This is the largest HIV clinic in Washington state.  **Inclusion criteria:** HIV-infected, has completed one or more visits in the past 1000 days, but has not completed a visit in the prior 12 months. Identified as out of care from 1 November 2012 to 1 November 2013.  **Exclusion criteria:** Died or transferred clinics.  **Participants at baseline:** 646 patients in the “historical” control cohort, 753 in the re-linkage intervention cohort. 363 patients were included in both cohorts. 39% MSM, 6% IDU, 16% heterosexual, 14% MSM-IDU 84% male.  **Age at baseline**: 10% younger than 30, 24% 30-39 years of age, 33% 40-49 years of age, 33% 50 years of age or older.  **Race/ethnicity at baseline**: 60% white, 21% black, 12% Hispanic, 4% Native American, 0% (N=2) Pacific Islander.  **Median viral load at last known lab:** Not given  **Median CD4 at last known lab:**  Not given. 5% CD4 levels below 500 cells/µL, 2% CD4 levels above 500 cells/µL, 93% missing. | **Intervention Description:** Two parts to the intervention: use of HIV surveillance data to identify out-of-care patients, and then contact/outreach by a designated linkage specialist to help patients re-engage in care. Contacts included 3 phone calls, 1 email attempt, 1 outside agency contact, and 1 emergency contact. If not successful, additional case investigation and outreach efforts were done by the county relinkage outreach team. When needed, the linkage specialist offers to  meet patients outside of the clinic, assists with transportation,  or in the case of hospitalized patients, visits patients in the  inpatient unit.  **Control:** Patients out-of-care at the Madison Clinic between November 1 2011 and November 1 2012. Standard of care, no intervention received. Some patients were in both cohorts. | **Primary:** Number of patients re-engaged in HIV care. Defined as a patient returning to the clinic for an appointment.  **Secondary:** Time from contact to relinkage; proportion of patients contacted who relinked to care.  **Assessed at:** 12 months (end of observation period) |
| **Bupamba 2010** | **Design:** Cohort  **Participants in the Cohort**: HIV patients in Tanzania  **Unit of randomization/study**: Patients  **Duration:** October 2008 to March 2009 | **City/State/Country:** Tanzania (national)  **Setting:** 20 Care and Treatment Clinics across Tanzania. Clinics are supported by the Columbia University’s International Center for AIDS Care and Treatment Programs (ICAP) Tanzania.  **Inclusion criteria:** Patients with missing 3 ore more appointments in row  **Participants at baseline:** 966 patients identified as missing appointments.  **Age at baseline**: Not given.  **Race/ethnicity at baseline**: Not given.  **Median viral load at last known lab:** Not given.  **Median CD4 at last known lab:** Not given. | **Intervention Description:**  Peer educators selected and trained. Training included risk reduction, ART side effects, HIV education, and psychosocial and adherence support, as well as support and supervision by clinic staff. They support linkage in the community, escort patients on visits, run counselling sessions.  Peer educators would also receive lists of patients identified as out-of-care (missed three or more appointments in a row) and attempt to trace them and encourage them to return to care. Tracing could involve phone calls or home visits to patients.  **Control:** Not applicable. | **Primary:** Re-engaged in care. Defined as attending a clinic visit.  **Secondary:** Successfully contacted; reasons for defaulting.  **Assessed at:** 6 months. |
| **Chikuse 2019** | **Design:** Cohort  **Participants in the Cohort**: HIV+ patients in Malawi  **Unit of randomization/study**: Patients  **Duration:** October to December 2017 | **City/State/Country:** Malawi  **Setting:** 69 health facilities supported by EQUIP across Malawi.  **Inclusion criteria:** Clients with missed ART appointment  **Participants at baseline:** 5651 clients in Malawi who had missed an appointment  **Age at baseline**: Not given.  **Race/ethnicity at baseline**: Not given.  **Median viral load at last known lab:** Not given.  **Median CD4 at last known lab:** Not given. | **Intervention Description:** Expert Clients (ECs) were HIV-positive people who trained in a 2-day workshop to assist to re-engage in care their fellow HIV+ community members who missed appointments. Patients were contacted by phone calls, SMS, or home visits to be encouraged to re-engage in care.  **Control:** Not applicable. | **Primary:** Number of patients returned to care. Definition not given.  **Assessed at:** Not clear. Assumed to be 3 months (October to December 2017). |
| **Deery 2014** | **Design:** Cohort  **Participants in the Cohort**: HIV+ patients in Johannesburg  **Unit of randomization/study**: Patients  **Duration:** June until December 2012 | **City/State/Country:** Johannesburg, South Africa  **Setting:** Witkoppen Health and Welfare Center in Johannesburg, South Africa.  **Inclusion criteria:** One of five types of index case: Newly diagnosed TB or HIV case, or individuals lost to TB care (2 weeks or later to dispensing appointment), pre-ART care (no CD4 test or missed ART initiation appointment by one month or more), or ART care (missed dispensing appointment by one month or more). Index cases had to present between June and December 2012.  **Exclusion criteria:** Deceased, incarcerated, missing outcomes.  **Participants at baseline:** 419 household successfully contacted. 755 lost to ART care and treatment  **Age at baseline**: Not given.  **Race/ethnicity at baseline**: Not given. 28.1% of study population were immigrants.  **Median viral load at last known lab:** Not given.  **Median CD4 at last known lab:** Not given. | **Intervention Description:** Eligible index cases identified in chart reviews. Lay health workers certified in HCT and trained in TB services called patients to confirm details, and a home visit was planned. At household visits, HIV testing offered, TB screening, adherence counseling provided, sputum sample collected, and patients encouraged to return to care and schedule an appointment.  **Control:** Not applicable. | **Primary:** Returned to care. Defined as returning to receive care within one month of home visit.  **Secondary:** Percent of households successfully visited; median time to reengagement in care  **Assessed at:** One month (primary), six months (secondary) |
| **Donovan 2018** | **Design:** Cohort  **Participants in the Cohort**: Patients attending HIV clinics in North Carolina  **Unit of randomization/study**: Patients  **Duration:** 1 January 2013 to 31 December 2014 | **City/State/Country:** North Carolina, USA  **Setting:** The NC-LINK study was conducted across five clinics in four different regions of HIV service delivery in North Carolina. Clinics hosted between 400 and 1950 HIV patients. Two were infectious disease clinics, two were rural clinics, and one was a clinic within a regional medical facility.  **Inclusion criteria:** 18+ years of age who did not attend any clinic appointments for 6 or 9 months (exact length of time depends on clinic).  **Participants at baseline:** 1118 HIV+ attendees identified as out-of-care across the 5 clinics. 1038 used in the final analysis: 40% MSM, 9% IDU/IDU-MSM, 49% heterosexual. 70% male.  **Age at baseline**: 41% were 18-29 years of age, 31% were 30-39 years old, 21% were 40-49 years old, 7% were 50 or older.  **Race/ethnicity at baseline**: 23% white, 71% black, 3% Hispanic, 3% other.  **Viral load at last known lab:** 62% suppressed (<200 copies/mL).  **Median CD4 at last known lab:** Not given. | **Intervention Description:** Staff contacted out-of-care patients to encourage them to return to care. Contact was first attempted by phone and letter. If patient could not be contacted or did not return to care, then clinic staff referred patients to specially trained State Bridge Counselors to attempt contact and an in-person visit.  **Control:** Not applicable. | **Primary:** Returned to care. Defined as the presence of a viral load test result within 90 days of referral.  **Secondary:** Retention defined as two lab markers within the follow-up year at least 90 days apart; virally suppressed at 180 days;  **Assessed at:** 90 days (primary), 180 days (virally suppressed). |
| **Dufour 2018** | **Design:** Cohort  **Participants in the Cohort**: HIV+ patients in the United Kingdom  **Unit of randomization/study**: Patients  **Duration:** Not clear. Database of lost to follow-up patients built in September 2017. | **City/State/Country:** United Kingdom  **Setting:** Large HIV clinic located in the United Kingdom, no further information available.  **Inclusion criteria:** HIV+ patients at the clinic who had not presented for HIV care in 8 months or longer and with no scheduled appointments.  **Exclusion criteria:** Transferred clinics, incorrectly received HIV care (HIV negative), deceased.  **Participants at baseline:** Not given.  **Age at baseline**: Not given.  **Race/ethnicity at baseline**: Not given.  **Median viral load at last known lab:**  Not given.  **Median CD4 at last known lab:** Not given. | **Intervention Description:** Out-of-care patients were identified using the hospital IT system and marked as potentially lost to follow-up. These patients were then assessed using clinic notes to assess those truly out-of-care, and these patients were contacted by patients through two phone call attempts. On the calls, patients were encouraged to return to care by clinic staff.  **Control:** Not applicable. | **Primary:** Re-engaged in care. Defined as returning for a clinic appointment.  **Secondary:** Percent successfully contacted; percent of those successfully contacted re-engaged in care.  **Assessed at:** Not given. |
| **Edwards 2019** | **Design:** Retrospective Cohort  **Participants in the Cohort**: Patients lost to follow-up between July 2016 and March 2017 in Trinidad and Tobago  **Unit of randomization/study**: Patients  **Duration:** April 2017 to September 2017. | **City/State/Country:** Trinidad and Tobago  **Setting:** The Medical Research Foundation of Trinidad and Tobago, the largest HIV treatment site in Trinidad and Tobago. Cares for about half of the nation’s people living with HIV.  **Inclusion criteria:** Patients who had been out of care for 3 months or longer.  **Participants at baseline:** 1058 patients identified as potentially lost to follow-up, of these 866 eligible, 701 reached. For those reached: 6.1% MSM, 6.6% bisexual, 6.3% drug user, 48.7% male.  **Race/ethnicity at baseline**: 62.9% African, 5.1% East Indian, 16.7% mixed.  **Median viral load at last known lab:** Not given.  **Median CD4 at last known lab:** Not given. | **Intervention Description:** Trained social workers used EMR to develop a list of patients. Patients were then traced and contacted via phone. Once contacted, patients were consulted on reasons for not returning to care, barriers to accessing care, and encouraged to return to care. Some patients were contacted multiple times.  **Control:** Not applicable. | **Primary:** Returned to care. Defined as patients who attend their clinic visits after contact within their scheduled time frame for follow up.  **Secondary:** Restarted ART, retained in care  **Assessed at:**  retained in care > 6 months |
| **Fanfair 2019** | **Design:** Randomized Control Trial (individual level)  **Participants in the Cohort**: HIV+ patients attending CoRECT clinics in Connecticut, Massachusetts, and Philadelphia.  **Unit of randomization/study**: 1:1 randomization at the individual level to the intervention and the standard of care (control) arms.  **Duration:** 16 August 2016 to 31 July 2018. | **City/State/Country:** Connecticut, Massachusetts, and Philadelphia  **Setting:** 41 clinics in Connecticut (23), Massachusetts (10 clinics), and Philadelphia (8). Clinics supervised by the Connecticut Department of Public Health/Yale University School of Medicine, Massachusetts Department of Public Health, and the Philadelphia Department of Public Health, respectively.  **Inclusion criteria:** 18 years or older. Had received HIV care at a CoRECT clinic and subsequently was identified as out-of-care. Out-of-care defined as: having no CD4 or viral load surveillance data for 6 months or longer, or having a missed appointment/no clinic appointment in 6 months or longer.  **Exclusion criteria:** Deceased, out of jurisdiction, changed providers, incarcerated  **Participants at baseline:** 1894 HIV+ attendees across the 41 CoRECT clinics (655 in Connecticut, 630 in Massachusetts, 600 in Philadelphia). 38.7% MSM, 19.7% IDU, 23.1% heterosexual.  **Age at baseline**: Not given.  **Race/ethnicity at baseline**: 25.6% white, 48.4% black, 23.8% Hispanic, 2.2% other.  **Median viral load at last known lab:** Not given.  **Median CD4 at last known lab:** Not given. | **Intervention Description:** Specialists and health workers actively attempt to locate and contact patients in an effort to encourage and facilitate re-entry to care.  **Control:** Standard of care for re-engagement. No active locating and contacting of patients by specialists or health workers to return to HIV care. | **Primary:** Re-engaged to care defined as having CD4 or viral load tests taken.  **Secondary:** Median time to re-engagement in care.  **Assessed at:** All outcomes assessed at 90 days post-randomization. |
| **Fox 2018** | **Design:** Randomized Control Trial (cluster level)  **Participants in the Cohort**: HIV+ patients in four provinces across South Africa  **Unit of randomization/study**: 1:1 matched randomization across 24 clinics. Matched on size, setting, proportion virally suppressed, and location  **Duration:** 20 June 2016 until 16 December 2016 | **City/State/Country:** South Africa  **Setting:** 24 clinics (12 to each arm) across four provinces in South Africa: Limpopo, North West, Gauteng, KwaZulu Natal.  **Inclusion criteria:** HIV+ on first-line ART, 18 years of age or older, living in one of the clinic’s catchment areas, in care between 20 June and 16 December 2016 who failed to attend scheduled appointment in 5 to 90 days of appointment date.  **Exclusion criteria:** Outside of catchment area from participating clinics, pregnant, or planning to transfer facilities.  **Participants at baseline:** 403 HIV+ patients enrolled in the Early Tracing study and 863 in the Enhanced Adherence Counselling study. 33% male. Analysis included all eligible patients during the intervention period at intervention (18707) and control site (17097) for Early Tracing.  **Age at baseline**: 19% are 18-29 years of age, 36% are 30-39 years of age, 27% are 40-49 years of age, 17% are 50 or older.  **Race/ethnicity at baseline**: Not given.  **Median viral load at last known lab:** 100 (IQR: 20-169)  **Median CD4 at ART Initiation:** 212 (114-335). | **Intervention Description:**  Maintenance of an EHR to identify patients at risk of being out-of-care (missed scheduled appointment in past 5-90 days). Tracing by trained outreach workers given a list of patients with missed appointments, with contact by phone and home visits.  **Control:** Standard of care for re-engagement. | **Primary:** Percent returning to care within 3 months.  **Secondary:** Percent retained in care for 12 months  **Assessed at:** 3 months (primary); 12 months (secondary) |
| **Healey 2018** | **Design:** Cohort  **Participants in the Cohort**: HIV+ patients in Sydney, Australia  **Unit of randomization/study**: Patients  **Duration:** Not clear. Study period began in 2013, with follow-up for new HIV patients through 2016 | **City/State/Country:** Sydney, New South Wales, Australia  **Setting:** HIV+ Patients in the Sydney Local Health District Sexual Health Service. Located in Inner City Sydney, Australia.  **Inclusion criteria:** HIV+ patients not attended for more than 4 months since their last appointment. Receiving clinic service in Sydney.  **Participants at baseline:** 23 patients identified as lost to care and contacted for follow-up. 69.6% MSM, 8.7% both sexes, 21.7% heterosexual. 90% male.  **Age at baseline**: Mean age: 35. Median age: 33 (range: 21-59).  **Race/ethnicity at baseline**: Not given.  **Median viral load at last known lab:** Not given.  **Median CD4 at last known lab:** Not given. | **Intervention Description:** The clinic social worker used a data audit to identify patients out of care in mid-2013. Out of care patients received personalized messages to return to care phone calls and texts, letters, and emails to patients, encouraging them to return to care.  **Control:** Not applicable. | **Primary:** Reengaged in care, definition not given.  **Secondary:** mean time absent from care before reengagement.  **Assessed at:** Not given. |
| **Keller 2017** | **Design:** Cohort  **Participants in the Cohort**: Patients attending HIV clinics in North Carolina  **Unit of randomization/study**: Patients  **Duration:** March 2013 until May 2014 | **City/State/Country:** North Carolina, USA  **Setting:** Un unspecified pilot site located in North Carolina. Site was host to the first testing of the NC-LINK retention Protocol.  **Inclusion criteria:** HIV+ patients identified as out-of-care, defined as not having received/attended any medical visits in the previous nine months.  **Exclusion criteria:** Patients with upcoming scheduled visits, patients with designated HIV clinic visits greater than 9 months apart, incarcerated, deceased, relocated  **Participants at baseline:** 1951 patients in the clinic population, 452 of whom are eligible. Of those eligible: 46% MSM, 4% MSM youth, 8% IDU, 43% heterosexual, 69% male, 1% transgender.  **Age at baseline**: 6% 17-24 years old, 23% 25-34 years old, 24% 35-44 years old, 33% 45-54 years old, 14% 55 or older.  **Race/ethnicity at baseline**: 70% African American, 25% White, 3% Hispanic, 2% other.  **Median viral load at last known lab:** Not given.  **Median CD4 at last known lab:** Not given. | **Intervention Description:** Monthly lists of out-of-care patients at the clinic were generated. Staff would evaluate the generated lists to verify if patients were out-of-care. Once verified, staff contacted out-of-care patients to encourage them to return to care using phone calls and letters. Contact was first attempted by phone and letter. If contacted, patients encouraged to re-engage in care at walk-in appointments or add-on appointments. Counseling with patients allowed personnel to identify and attempt to alleviate burdens to receipt of care. Patients unable to be successfully contacted were referred to a specialized State Bridge Counselor, to attempt to contact.  **Control:** Not applicable. | **Primary:** Percent returned to care.  **Assessed at:** during March 2013 and May 2014 (~ 12 months) |
| **Lubelchek 2016** | **Design:** Cohort  **Participants in the Cohort**: Individuals living in Cook County, Illinois  **Unit of randomization/study**: Patients  **Duration:** 1 April 2014 to 30 September 2014 | **City/State/Country:** Chicago, Illinois, USA  **Setting:** The Ruth M. Rothstein CORE Center, which is the Cook County Health and Hospital System’s (CCHHS) ambulatory HIV clinic. The clinic is located in Chicago, IL  **Inclusion criteria:**  HIV+, registered for a non-primary care visit at the Ruth M. Rothstein CORE Center. Lost-to-care, defined as not having a primary care visit in the past 7 months. No follow-up HIV clinic appointments in the next 3 months. Patients are not in care at another facility, and patients must reside in Cooke County, Illinois.  **Participants at baseline:** Hospital sees 5500 HIV+ individuals in the Chicago area. 396 alerts were sent, aimed to 198 patients. 115 determined eligible, 55 of whom had no upcoming HIV-specific clinic visits. Of those 55: 41% MSM, 73% male.  **Age at baseline**: Mean age: 45 years. IQR: (42-47).  **Race/ethnicity at baseline**: 78% African American, 12% Hispanic.  **Median viral load at last known lab:** 53% viral load undetectable.  **Median CD4 at last known lab:** 344 cells/mL IQR: (192-623). | **Intervention Description:** Program coordinators received real-time text message alert when lost-to-care patients registered for non–primary care visits. The coordinator attempted to visit the patients at their other appointment and encourage them to return to care. If unable to find them in-person, coordinator then attempts to call the patient on the phone.  **Control:** Not applicable. | **Primary:** Returned to care and attended HIV-specific clinic appointment.  **Secondary:** undetectable viral load at the latest laboratory test before the visit with program coordinator.  **Assessed at:** 3 months (Primary). |
| **Magnus 2012** | **Design:** Case-Cohort  **Participants in the Cohort**: HIV+ patients seeking medical care in Louisiana  **Unit of randomization/study**: Patients  **Duration:** 1 February 2009 until 31 July 2011. | **City/State/Country:** Louisiana, USA  **Setting:** Monitoring implemented across the state of Louisiana. In total 60 clinics identified patients, and clinicians were based at 7 facilities across the state of Louisiana.  **Inclusion criteria:** HIV+ patients presenting for non-HIV care at a participating Louisiana clinic, no CD4 or viral load testing in over 1 year.  **Exclusion criteria:** Decline HIV care or getting care outside the clinic system.  **Participants at baseline:** 996 HIV+ participants overall, 419 in intervention and 577 in the control. For intervention patients: 13.8% MSM, 3.8% IDU, 21.5% heterosexual. 63.0% male. For control patients: 9.4% MSM, 12.8 IDU, 31.7% heterosexual, 48.0% male.  **Age at baseline**: In intervention group: 46.4% younger than 35. In control group: 34.0% younger than 35.  **Race/ethnicity at baseline**: In intervention group: 68.0% black, 32.0% white or other. In control group: 89.1% black, 10.9% white/other.  **Median viral load at last known lab:** Intervention: 242 (IQR: 75-407). Control: 271 (IQR: 117-434).  **Median CD4 at last known lab:** Intervention: 31,333 (IQR: 2762 – 14,732). Control: 1255 (IQR: 248 – 32,380). | **Intervention Description:** Specialized information exchange between an Integrated Delivery Network and the Louisiana Public Health Information Exchange. System would send alerts to attending clinician when a known HIV+ individual who had not presented for HIV-specific care in over a year presented at a clinic for non-HIV care. Patients received counseling from clinicians with the aid of system provided prompts, and are encouraged to return to care and receive CD4 and viral load testing.  **Control:** Time-matched random sample of HIV patients in the Integrated Delivery Network who had presented for care at least once in the prior 5 years who had experienced a one-year or greater gap in care during that time span. | **Primary:** Percentage of patients receiving follow-up care (re-engaged in care). Defined as receiving a CD4 or viral load test.  **Secondary:** Retention in care of patient; median time out of care; median VL load  **Assessed at:** 6 months (for primary), 12 months (for secondary) |
| **McMahon 2015** | **Design:** Cohort  **Participants in the Cohort**: HIV+ patients in Victoria, Australia  **Unit of randomization/study**: Patients  **Duration:** February 2011 to June 2013 | **City/State/Country:** Victoria, Australia  **Setting:** Six different HIV clinics across the state of Victoria in Australia. Three GP clinics, two hospitals, and a sexual health clinic.  **Inclusion criteria:** Individual with unknown outcome at one of six study sites who received care from 3/1/2011-5/1/2013 (lab test) but did not return for follow-up before 2/28/2014.  **Participants at baseline:** 164 people with unknown outcomes pre-intervention, 91.5% male  **Age at baseline**: 39.5  **Race/ethnicity at baseline**: non-English speaking 24.2%  **Median viral load at last known lab:** 127 copies/ml | **Intervention Description:** Patients thought to have transferred or been lost to care had their medical records cross-referenced with other HIV care hospitals in the area to see if presenting for care elsewhere. If no transfer information found, patients contacted via phone to re-engage in care.  **Control:** Not applicable | **Primary:**  Returned in care.  **Assessed at:** 6 months (the tracing is done in the second six months of 2013) |
| **Nabaggala 2016** | **Design:** Cohort  **Participants in the Cohort**: Patients in North-Eastern Uganda  **Unit of randomization/study**: Patients  **Duration:** January 2014 until August 2015 | **City/State/Country:** Moroto, Uganda  **Setting:** Moroto Regional Referral Hospital is a government-run hospital located in rural North-Eastern Uganda, in the town of Moroto.  **Inclusion criteria:** HIV+ individuals attending the Moroto clinic between January 2014 and August 2015 who missed a clinic visit and had a medical record available in the clinic’s EHR database.  **Exclusion criteria:** Deceased, incarcerated, transferred.  **Participants at baseline:** 381 HIV+ attendees identified as missing an appointment. 32% male.  **Age at baseline**: Median age: 30 (IQR: 25-36).  **Race/ethnicity at baseline**: Not available.  **Median viral load at last known lab:** Not given.  **Median CD4 at last known lab:** Not given. | **Intervention Description:** Specially designated counsellor to track patients who missed a visit using phone calls and home visits (home visits by counsellor or hospital staff). Patients who were successfully contacted were encouraged to return to care. The counselor would spend three months trying to contact them for a missed visit before stopping.  **Control:** Not applicable. | **Primary:** Returned to care. Defined as attending a clinic visit during follow-up period.  **Secondary:** Returned to care after phone call; returned to care after home visit.  **Assessed at:** 3 months |
| **Nakiwogga-Muwanga 2015** | **Design:** Prospective Cohort  **Participants in the Cohort**: Patients at the Infectious Diseases Clinic in Kampala, Uganda  **Unit of randomization/study**: **Patient**  **Duration:** April 2011 to September 2013 | **City/State/Country:** Kampala, Uganda  **Setting:** The Infectious Diseases Clinic at Mulago Hospital. Mulago is the main HIV referral hospital in Uganda, located in Kampala. The clinic is associated also with Makerere University.  **Inclusion criteria:** 18 or older who missed their appointments for 8–90 days.  **Participants at baseline:** 4582 patients. Random sample of 25% selected. Baseline information given for those traced for 18 months after returning to care.  41% male.  **Age at baseline**: 27% were 25-34 years of age, 42% were 35-44 years old, 31% were 45 or older.  **Race/ethnicity at baseline**: Not given.  **Median viral load at last known lab:** Not given.  **Median CD4 at last known lab:** 345. IQR: 243-513. | **Intervention Description:** Standard health services (ART refills and prescriptions, CD4 tests) as well as patient tracking using home visits and phone calls.    **Control:** Not applicable. | **Primary:** Return to care.  **Secondary:** Retained in care; CD4 level  **Assessed at:** 3 months (Return to care); 6, 12 and 18 months (Retained in care); and 18 months (CD4 levels). |
| **Sitapati 2012** | **Design:** Cohort  **Participants in the Cohort**: Patients attending the Owen Clinic in San Diego  **Unit of randomization/study**: Patients  **Duration:** 1 February 2010 until 1 February 2011 | **City/State/Country:** San Diego, California, USA  **Setting:** Implemented at University of California, San Diego Medical Center’s Owen Clinic. The intervention was implemented as part of a Continuous Quality Improvement (CQI) program at the clinic.  **Inclusion criteria:** HIV+ patients attending the Owen Clinic with a clinic visit between Feb 1, 2009 and Jan 31, 2010; or between September 1, 2009 and August 31, 2010 in the second analysis group, with a subsequent gap of six months or longer before next clinic visit.  **Participants at baseline:** 2793 HIV+ participants identified in the database. 62.1% MSM, 12.6% IDU, 17.6% heterosexual. 85.4% male.  **Age at baseline**: 1.1% were 13-24 years of age, 43.1% were 25-44 years old, 52.8% were 45-64 years old, 3.1% were 65 or older.  **Race/ethnicity at baseline**: 52.6% white, 14.4% black, 26.9% Hispanic, 6.1% other.  **Median viral load at last known lab:** Not given.  **Median CD4 at last known lab:** Not given. | **Intervention Description:**  Training of retention specialists with database management, customer service, and navigating different EHRs. Also included training on adherence, substance abuse, and health education.  Retention specialist, quality specialist, and CQI committee to develop new retention tools including an algorithm to identify out-of-care patients, secure database, voicemail system for returned patient calls, and development of brochures.  Weekly meetings and monthly reports performed to refine tools throughout the trial.  **Control:** Not applicable. | **Primary:** Returned to care with intervention  **Secondary:** Returned to care on own; lost or unable to contact; future appointment scheduled  **Assessed at:** 6 months |
| **Tesoriero 2017** | **Design:** Cohort  **Participants in the Cohort**:  **Unit of randomization/study**: Patients  **Duration:** 1 September 2013 to 1 August 2014 | **City/State/Country:** New York (state), USA  **Setting:** 4 clinics in upstate New York, home to about 1/3 of all PLWHIV outside of NYC in the state.  **Inclusion criteria:** Alive and living in one of 4 specified counties in New York State (Erie, Monroe, Onondaga, Westchester). HIV+ with no diagnostic or prognostic lab results in the past 13-24 months (defined as out-of-care).  **Participants at baseline:** 1155 HIV+ individuals identified as out-of-care across the 4 counties. 233 confirmed out-of-care. 37.3% MSM, 12% IDU, 1.7% IDU-MSM, 36.9% heterosexual. 60.9% male.  **Age at baseline**: 16.3% were 20-29 years of age, 21.5% were 30-39 years old, 27% were 40-49 years old, 28.3% were 50-59, 6.9% were 60+ years of age.  **Race/ethnicity at baseline**: 26.2% white, 50.2% black, 12.9% Hispanic, 9% other, 1.7% unknown.  **Median viral load at last known lab:** Not given.  **Median CD4 at last known lab:** Not given. | **Intervention Description:** Specially trained Health workers searched the Regional Health Information database for New York, identifying individuals suspected to be out of care. Attempted to contact patients and run vital identification of patients to confirm identify and discuss about eligibility. Medical providers were contacted, and phone, letter, and in-person house visits were conducted to attempt to contact patients. Once contacted and identified, out of care patients were screened for behavioral risk factors, given risk-reduction counseling, helped patients self-notify partners, offered supportive service referrals, and updated database based on trained data entry procedures.  **Control:** Not applicable. | **Primary:** Re-engaged in care. Defined as having a laboratory test within 6 months of case closure.  **Secondary:** Relinkage to care (attending at least one medical appointment prior to case assignment); retention in care (two or more lab tests within 6 months of case closure).  **Assessed at:** 6 months |
| **Tweya 2010** | **Design:** Cohort  **Participants in the Cohort**: Patients attending HIV clinics in Lilongwe, Malawi  **Unit of randomization/study**: Patients  **Duration:** April 2006 until March 2009 | **City/State/Country:** Lilongwe, Malawi  **Setting:** Two ART clinics located in urban Lilongwe, Malawi: Kamzu Central Hospital, part of Lighthouse, and the Martin Preuss Centre.  **Inclusion criteria:**  HIV+; attending either the Lighthouse Clinic or Martin Preuss Center; missed ART dispensing by three weeks or more; receiving ART at clinic between April 2006 and March 2009.  **Exclusion criteria:** Deceased, did not consent/rejected, missing outcomes.  **Participants at baseline:** 2690 HIV+ attendees participated in the study. 44% male (intervention group).  **Age at baseline**: 6% were children aged 14 or less (intervention group).  **Race/ethnicity at baseline**: Not given.  **Median viral load at last known lab:** Not given.  **Median CD4 at last known lab:** Not given. | **Intervention Description:**  A receptionist and three trained health workers would receive weekly lists of patients lost to follow-up (defined as having missed an ART dispensing by 3 weeks or more). They would attempt phone call and home visits to ascertain status of patient and confirm eligibility. Patients who were alive and had not transferred clinics were encouraged to return to care, and when possible a visit was scheduled. Patients who missed these new appointments were contacted two additional times to encourage returning to care.  **Control:** Not applicable. | **Primary:** Percent returned to ART clinic (attended a visit after being contacted).  **Secondary:** Percent promised to return to ART clinic; percent successfully traced; percent lost to follow-up.  **Assessed at:** Not given. |
| **Udeagu 2013** | **Design:** Cohort  **Participants in the Cohort**: Patients attending HIV clinics in New York City  **Unit of randomization/study**: Patients  **Duration:** July 2008 until December 2010 | **City/State/Country:** New York City, New York, USA  **Setting:** HIV+ patients attending HIV clinics in New York City.  **Inclusion criteria:** HIV+ patients in the New York City HIV surveillance registry. Lost to follow-up between July 2008 and December 2010. Lost to follow-up defined as no CD4 or viral load testing in prior 9 months. Had NYC address and care provider at their last study visit.  **Exclusion criteria:** Incarcerated, current to care, deceased.  **Participants at baseline:** 797 thought eligible, 684 located, 409 met eligibility criteria. Of 409 patients: 15% MSM, 25% IDU, 3% perinatal, 24% heterosexual. 55% male.  **Age at baseline**: <1% were 13-19 years of age, 12% were 20-29 years old, 22% were 30-39 years old, 42% were 40-49 years of age, 18% were 50-59, 6% were 60 or older.  **Race/ethnicity at baseline**: 2% white, 67% black, 30% Hispanic, <1% Asian/Pacific Islander, <1% other.  **Median viral load at last known lab:** Not given. 62% suppressed (<200 copies/mL).  **Median CD4 at last known lab:** Not given. | **Intervention Description:** NYC registry used to identify HIV+ individuals and those lost to follow-up during the study period. Case workers attempted to trace and contact out-of-care via phone calls, letter or home visits. When contacted, case workers would encourage patients to return to care.  **Control:** Not applicable. | **Primary:** Returned to care, defined as any CD4/VL test.  **Assessed at:** 12 months |
| **Udeagu 2018** | **Design:** Cohort  **Participants in the Cohort**: HIV+ patients in New York City  **Unit of randomization/study**: Patients  **Duration:** January 2009 to December 2013 | **City/State/Country:** New York City, New York, USA  **Setting:** Patients and clinics in New York City, USA, who were present in the New York City Health Department and NYC HIV Surveillance Registry.  **Inclusion criteria:** Traced in the NYC HIV Surveillance Registry, 18+ years of age, defined as out-of-care (lack lab reports in previous 9 months), agree to link to care following contact by a public health worker.  **Participants at baseline:** 543 patients re-engaged in care and had lab tests after return visit. 23% MSM, 23% IDU, 40% heterosexual. 59% male.  **Age at baseline**: 12% 19-29 years of age, 55% 30-49 years of age, 33% 50 years or older.  **Race/ethnicity at baseline**: 6% white, 59% black, 34% Hispanic, 1% other.  **Median viral load at last known lab:** Not given.  **Median CD4 at last known lab:** Not given. | **Intervention Description:** Patients identified as out-of-care were assigned to a case worker, who would attempt to contact and re-engage the patients through phone calls, emails, and home visits, and patients were interviewed to identify out-of-care status and reasons for being out-of-care. Patients were then linked to the best resource (counseling, clinic, etc) to get them to re-engage in care.  **Control:** Not applicable. | **Primary:** Had one or more HIV care visit (VL test) within first year of follow-up  **Secondary:** Median time to viral testing from re-engagement in care; number with two or more CD4 tests in last 3 months; out-of-care within one year and never re-engaged; percent continuously engaged in care; viral load <1500 mL; virally suppressed (<200/mL);  **Assessed at:** 365 days for primary. All outcomes assessed annually at 1, 2, 3, 4, and 5 years. |
| **Udeagu 2019** | **Design:** Trial with a control arm (assessed as RCT)  **Participants in the Cohort**: Patients attending HIV clinics in New York City  **Unit of randomization/study**: Patients  **Duration:** March 2016 until October 2017 | **City/State/Country:** New York City, New York, USA  **Setting:** A collaboration between an HIV clinic in New York City and the New York City Department of Health and Mental Hygiene.  **Inclusion criteria:** PLWH in NYC, found in the NYC HIV surveillance system, had no viral load or CD4 cell count reports in the NYC surveillance registry for at least 9 months. Primary clinic is the collaborating clinic in the study.  **Exclusion criteria:** Deceased, incarcerated, or missing data  **Participants at baseline:** 3527 individuals, 184 in the intervention group and 3343 in the routine care group. 38.3% MSM, 15.3% IDU, 24.9% heterosexual. 68.6% male, 1.3% transgender male-to-female.  **Age at baseline**: Not given.  **Race/ethnicity at baseline**: 11.5% white, 57.3% black, 28.9% Hispanic, 2.2% other.  **Median viral load at last known lab:** Not given.  **Median CD4 at last known lab:** Not given. | **Intervention Description:** The Department of Health and Mental Hygiene identified patients out-of-care at the collaborating HIV clinic and verified out-of-care status by comparing their medical records. Out-of-care patients were compiled in a list and shared with the clinic’s patient navigator and a disease intervention specialist from the Department of Health and Mental Hygiene to attempt to contact the patient and encourage returning to care through phone calls and home visits.  **Control:** Standard of care. OOC patients identified by the NYC Department of Health, but no special attempts made to contact them. | **Primary:** Percent reengaged in care  **Secondary:** Percent reengaged in care at the collaborating clinic; percent reengaged who re-initiated ART; virally suppressed.  **Assessed at:** during March 2016 until October 2017 (18 months) |
| **Villanueva 2019** | **Design:** Randomized Control Trial (individual level)  **Participants in the Cohort**: HIV+ patients attending CoRECT clinics in Connecticut.  **Unit of randomization/study**: 1:1 randomization at the individual level to the intervention and the standard of care (control) arms.  **Duration:** November 2016 to July 2018. | **City/State/Country:** Connecticut, USA  **Setting:** 23 HIV clinics in Connecticut. The Yale School of Medicine and Connecticut Department of Health supervised and worked at the HIV centers for the study.  **Inclusion criteria:** Had received HIV care at a CoRECT clinic and subsequently was identified as out-of-care. Out-of-care defined as: having no CD4 or viral load surveillance data for 6 months or longer, or having a missed appointment/no clinic appointment in 6 months or longer.  **Exclusion criteria:** Deceased, out of jurisdiction, changed providers, incarcerated  **Participants at baseline:** 655 HIV+ patients attending CoRECT HIV clinics in Connecticut. 29.6% MSM%, 27.2% IDU, 29.0% heterosexual, 3.5% MSM and IDU. 62.4% male.  **Age at baseline**: Mean age: 46.1 years. Median age: 47.3 years.  **Race/ethnicity at baseline**: 20.8% white, 40.3% black, 36.9% Hispanic, 2.0% other.  **Median viral load at last known lab:** Mean: 7,514.7 copies/mL. Median: 20 copies/mL.  **Median CD4 at last known lab:** Mean: 616.9 cells/µL. Median: 570 cells/µL. | **Intervention Description:** Specialists and health workers actively attempt to locate and contact patients in an effort to encourage and facilitate re-entry to care.  **Control:** Standard of care for re-engagement. No active locating and contacting of patients by specialists or health workers to return to HIV care. | **Primary:** Number re-engaged/returned to care. RE-engaged in care was defined as having CD4 or viral load tests taken, or having a patient or kept their scheduled clinic appointments.  **Secondary:** Median time to re-engagement in care. Number re-engaged in care (by meeting the lab test definition). Number re-engaged in care (by meeting the appointment definition). Viral load and CD4 levels post-randomization.  **Assessed at:** All outcomes assessed at 90 days post-randomization. |
| **Wohl 2016** | **Design:** Single-arm trial  **Unit of randomization/study**: out-of-care patients  **Duration:** January 2012 to August 2014 | **City/State/Country:** LA, California, USA  **Setting:** Seven publicly funded Los Angeles Country (LAC) HIV clinics  **Inclusion criteria:** Over 18 years, a resident of LAC, HIV positive, and a current or past patient at one of the study clinics who had any of the following criteria (1) no HIV care visits in the last 6–12 months and last viral load was greater than 200 copies/ml; (2) no HIV care visits in 12 months; (3) newly diagnosed and never in care; or (4) recently released from jail/prison/other institution with no regular HIV medical provider.  **Participants at baseline:** 78 out-of-care patients.  Demographic characteristics:  65% 40 years or older, 78% male, 4% transgender, 18% African American, 6% White, 71% Latino, 5% other  Sexual Orientation: 50% homosexual / 6% bisexual / 44% heterosexual  Insurance coverage: 57% no insurance  Annual income: 64% more than $10,000  Employment: 31% employed  Education: 21% less than high school  Substance use in past 6 months: 8% injection/ 25% non-injection | **Intervention description:**  Six navigators with bachelor-level degree and experience in HIV case management who most were bilingual in Spanish trained to locate and enroll patients to a modified 90-day CDC-approved ARTAS intervention. The navigators contacted patients by phone, text, e-mail, letter, and an in-person home visit. Each technique was attempted no more than 3 times before the next technique was used. Additional clinical and public websites, jail and shelter databases also searched if the above techniques were not successful. The modified intervention eliminated the incentives but included 10 navigation program visits (in 4 parts: building the relationship, assessment, linking to resources/enhancing strengths, and disengagement), and added a new tool to assess readiness to engage in care and collection of details locator information. Participants received telephone, text, or e-mail reminders about upcoming navigation program visits. At the completion of the intervention or any time during the intervention, participants could be linked to medical care.  **Control:** Not applicable | **Primary:**  Linkage to care was defined as either 2 medical visits or 1 medical and 1 case management visit.  Retention in care that was defined as a second viral load (VL) test 3–12 months after linkage to care.  **Assessed at:**  Linkage to care measured at 3, 6 and 12 months after intervention  **Secondary:** The percentage of participants who were virally suppressed (<= 200 copies/ml)  **Assessed at:** pre-enrollment, at the time of linkage, and 3–12 months after linkage to care. |
| **Rebeiro 2017** | **Design:** Retrospective Cohort  **Participants in the Cohort**: Patients attending Academic Model Providing Access to Healthcare (AMPATH) clinics  **Unit of randomization/study**: Patients  **Duration:** 1 January 2001 to 31 December 2011 (estimated) | **City/State/Country:** Kenya  **Setting:** AMPATH clinics across Western Kenya. AMPATH is a partnership between Moi University School of Medicine, Brown Medical School, and Indiana University School of Medicine. There are 19 AMAPATH HIV clinics located across western Kenya, serving 50,000+ patients. Most care is provided for free, including ART and lab tests.  **Inclusion criteria:** Receiving care at an AMPATH clinic. Missed one or more study appointments during the years 2001-2011. Not deceased or in care at another clinic at time of missed appointment.  **Exclusion criteria:** Deceased, transferred care.  **Participants at baseline:** 108221 enrolled patients, 34522 lost to clinic (missed an appointment). 15331 (44.4%) confirmed to have missed an appointment (gap in care). 2754 (8%) deceased, 837 (2.4%) transferred care. 15600 (45.2%) unsuccessfully contacted. Of those unsuccessfully contacted, 8762 (56.2%) later returned to care of their own accord. In total, 24093 had a confirmed gap in care. Vital status was imputed for a further 6838 patients who were unsuccessfully contacted.  21091 (68.2%) female.  **Age at baseline**: Median age: 35.9 years (IQR: 29.8, 43.2)  **On ART at time of missing appointment**: 16972 (54.9%) patients.  **Had Disclosed HIV Status:** 18599 (64.4%) patients.  **Median CD4 at last known lab:** 255 cells/µL (IQR: 117, 444) | **Intervention Description:** The AMPATH clinics established an outreach program for following up with patients who missed a scheduled appointment. Patients traced based on information they shared with the clinic at enrollment. If patients who missed an appointment are found, they are counseled and encouraged to return to care.  Successful outreach was either obtaining vital status from patients who were deceased, or establishing contact with a living patient.  **Control:** Not reached by outreach. | **Primary:** Returned to care at one year after missed appointment. No definition given for returned to care (probably presenting for care at any point during follow-up).  **Assessed at:** one year |
| **Sharp 2019** | **Design:** Retrospective Cohort  **Participants in the Cohort**: Patients living with HIV who registered in emergency department without CD4 or HIV1-RNA in the prior 14 months in the department public health database.  **Unit of randomization/study**: Patients  **Duration:** 1 January 2017 to 31 January 2018 | **City/State/Country:** United States  **Setting:** The Grady Health System (GHS) in Atlanta, Georgia in partnership with the Georgia Department of Public Health has generated a health information exchange (HIE) alarm system that utilizes GHS social workers to provide linkage resources and motivation to out-of-care patients who present to the GHS emergency department.  **Inclusion criteria:** All adult patients (18+ years) who generated an HIE alert (as they did not have CD4 or HIV1-RNA in the prior 14 months in public health data) and also self-reported out of care.  .  **Exclusion criteria:** Patients who were admitted to the hospital were excluded from the analysis.  **Participants at baseline:** 98 enrolled patients (78 received the social work intervention, 20 did not).  **Age at baseline**: Mean age: 41.0 years (SD 12.3)  **Median CD4 at last known lab:** 395.8 cells/µL (SD: 236.6) | **Intervention Description:** The HIV alerts were sent to two clinicians, who notified one or two social workers (SW) at the emergency department. Then, the SWs approached the patient, explained the system, and ascertained if the patient was engaged in HIV care. If not, the SW educated the patient, assessed barriers to care, and offered re-linkage support, either to the prior site of care or the Grady HIV clinic. Patients were able to walk into the Grady HIV clinic to initiate enrollment and see a prescribing physician within 72 hours without an appointment. Depending on patient preferences, the SW did  follow up with reminder calls.  **Control:** The group of patients who generated an HIE alert but who were not contacted by the HIE SW in the emergency department or admitted to the hospital. | **Primary:** Returned to care that was defined as any visit with an HIV provided within GHS and/or any CD4 or HIV1-RNA result within department of public dataset during 6-months follow up.  **Assessed at:** 6 months |
| **Saafir-Callaway 2020** | **Design:** Prospective Cohort  **Participants in the Cohort**: Patients identified as out-of-care by seven Ryan White funded primary care providers  **Unit of randomization/study**: Patients  **Duration:** April 2012 to April 2013 | **City/State/Country:** Washington, DC, USA  **Setting:** Seven Ryan White clinics around the District of Columbia. Surveillance data reported from the clinic by primary care providers to the District of Columbia Department of Health.  **Inclusion criteria:** HIV+, receiving HIV care in the District of Columbia. Identified as out of care (no evidence of a viral load result, CD4 result, or care visit for the immediate past 6-12 months).  **Participants at baseline:** 686 individuals identified as potentially out of care, of whom 103 re-reengaged in care of whom did not have evidence of being in care.  **Age at baseline**: 40.7% 20-39 years old.  **Race/ethnicity at baseline**: 77.7% black | **Intervention Description:** HIV care and health services offered to patients, with additional surveillance, monitoring, tracking, and identification of patients lost to follow-up. Patients matched to the Washington DC Department of Health surveillance databases. Clinical providers attempted 60 days to trace those identified as out-of-care and re-engage in care. Re-engagement attempts include phone calls, letters, e-mail, in-person conversations, and text messages among other strategies.  **Control:** Not applicable | **Primary:** Number of patients successfully re-engaged in care.  **Secondary:** retention in care defined as at least one CD4 or VL test results during 6, 12, and 18 months after returning to care, and viral suppression defined as HIV VL < 200 copies/ml at the same time periods after re-engagement.  **Assessed at:** 6, 12, and 18 months. |
| **Alizadeh 2019** | **Design:** Prospective Cohort  **Participants in the Cohort**: Patients identified as out-of-care in a rural district hospital in Kisoro, Uganda.  **Unit of randomization/study**: Patients  **Duration:** May 2015 to April 2016. | **City/State/Country:** Uganda  **Setting:** HIV clinic in a rural district hospital in Kisoro, Uganda.  **Inclusion criteria:** HIV+ patients with missed 2 monthly appointments as identified through bi-weekly chart review.  **Participants at baseline:** 691 individuals identified as out of care.  **Baseline data**: Not provided | **Intervention Description:** hospital HIV clinic worked with staff who spend about 20 days monthly making outreach visits by motorcycle in search of approximately 130 out of care patients. If the patient is located, the fieldworkers first administered a brief disease-specific survey inquiring about reasons for not returning to the clinic and then discusses important aspects of the disease emphasizing the role of continuous care. Patients were encouraged to return and given an appointment and a note to provide to the clinic staff. The fieldworkers motivated by a point-based stipend system if they successfully found and interview with the lost to care patient or determined what happened to them.  **Control:** Not applicable | **Primary:** Number of patients successfully return to clinic.  **Secondary:** None  **Assessed at:** unknown |
| **Kunzweiler 2019** | **Design:** Cohort  **Participants in the Cohort**: Patients who were not in care at Massachusetts Department of Public Health and participating community health centers  **Unit of randomization/study**: Patients  **Duration:** Between October 2015 and June 2017 | **City/State/Country:** Massachusetts, USA  **Setting:** 6 funded CHCs  **Inclusion criteria:** HIV+ patients at a participating CHC, over18 years of age, and a current resident of Massachusetts.  LTFU were defined as not having a CD4 or viral load test completed in the previous 6 months.  **Participants at baseline:** 1418 patients were considered as potentially lost to care. 69.9% were male, 27.7% were MSM, and 22.9% were PWID.  **Age at baseline**: 19.3% were 18-29 years of age, 54.2% were 30-49 years, and 26.5% were over 50 years old.  **CD4 at enrollment:** Not Given | **Intervention Description:**  Patients who were confirmed by both the MDPH and the CHC as out-of-care were assigned to MDPH field epidemiologists in order to perform follow-up and contact patients by telephone, text messages, letters, or home visits.  **Control:** NO | **Primary:** re-engaged in care  **Assessed at:** up to 90 days after being identified as out of care.  **Secondary**: Retention to Care  **Assessed at:** having at least 2 CD4 and/or viral load tests completed before 90 days apart within 1 year of being identified as out-of-care  **Tertiary:** Viral Suppression  **Assessed at:** 200 viral copies/mL at the last viral load completed in the year after being identified as out-of-care. |
| **Fernández-Luis 2019** | **Design:** Prospective Cohort  **Participants in the Cohort**: HIV+ children at the MDH between February 2013 and March 2017  **Unit of randomization/study**: Patients  **Duration:** (Tracing) July 2016 to March 2017 | **City/State/Country:** Mozambique  **Setting:** A District Hospital  **Inclusion criteria:** HIV+ children of age over 15 years in care at MDH.  LTFU were defined as not attending the clinic for 120 days after last attended visit  **Participants at baseline:** 269 children were considered as potentially lost to care. 59.7% were male.  **Age at baseline**: median 7.68 for children who were reached and median 8.62 for children who could not be found  **CD4 at enrollment:**  High: 51.35% among reached children and 49.53% among not found children  Low: 35.14% among reached children and 20.56% among not found children  Unknown: 13.51% among reached children and 29.90% among not found children | **Intervention Description:** Children LTFU were identified monthly from the MDHIVPed, and caregivers were contacted by telephone using the contact information that was registered in the patient chart on enrollment in HIV care.  **Control:** NO | **Primary:** re-engaged in care  **Assessed at:** Up to 3 months after intervention |
| **Naidoo 2019** | **Design:** Cohort  **Participants in the Cohort**: HIV+ patients attended to 12 PHC facilities in rural Mopani District, South Africa  **Unit of randomization/study**: Patients  **Duration:** January through September 2017 | **City/State/Country:** South Africa  **Setting:** 12 PHC facilities  **Inclusion criteria:** HIV+ patients visited by CHWs from the PHC facilities  LTFU was defined as not patients who were not found in at least one register within 2 weeks after referral.  **Participants at baseline:** 864 patients were considered for tracing. 27% were male.  **Age at baseline**: median age was 38 years (0–92 years).  **CD4 at enrollment:**  Not Assessed | **Intervention Description:** CHWs tried to conduct home visit for LTFUs and to refer them back to the PHC facility.  **Control:** NO | **Primary:** re-engaged in care  **Assessed at:** Not Given |
| **Chang 2019** | **Design:** Prospective Cohort  **Participants in the Cohort**: HIV+ patients residing in King County, Washington who enrolled in PHSKC’s D2C program, the Care and Antiretroviral Promotion Program (CAPP).  **Unit of randomization/study**: Patients  **Duration:** Between 3/2012 and 5/2016 | **City/State/Country:** Seattle & King County, Washington, USA  **Setting:** Public Health—Seattle and King County (PHSKC)  **Inclusion criteria:** HIV+ patients participated in PHSKC’s D2C program. Poorly engaged in care was defined as having no CD4 or viral load reported to surveillance for≥12 months or a viral load of>500 copies/mL at last report≥6 months after HIV diagnosis.  **Participants at baseline:** 408 patients who were participated in the CAPP program. 86% were male, 55% were MSM, and 10% were PWID.  **Age at baseline**: 25% were below 34 years, 29% were 35-44 years, 35% were 45-54 years, and 11% were over 55 years. (1% were missing)  **CD4 at enrollment:**  13% had a CD4 count of over 500 (stage 1)  25% had a CD4 count of 200-499 (stage 2)  11% had a CD4 count of below 200 (stage 3)  50% were missing | **Intervention Description:** health department disease intervention specialists (DIS) contacted the last known medical provider for each eligible case then attempted to contact the individual PLWH and offer enrollment in CAPP  **Control:** NO | **Primary:** re-engaged in care  **Assessed at:**  12 months after the initial CAPP encounter |

## Appendix E - Supplementary Figures and Tables

## Re-engagement contact outcome


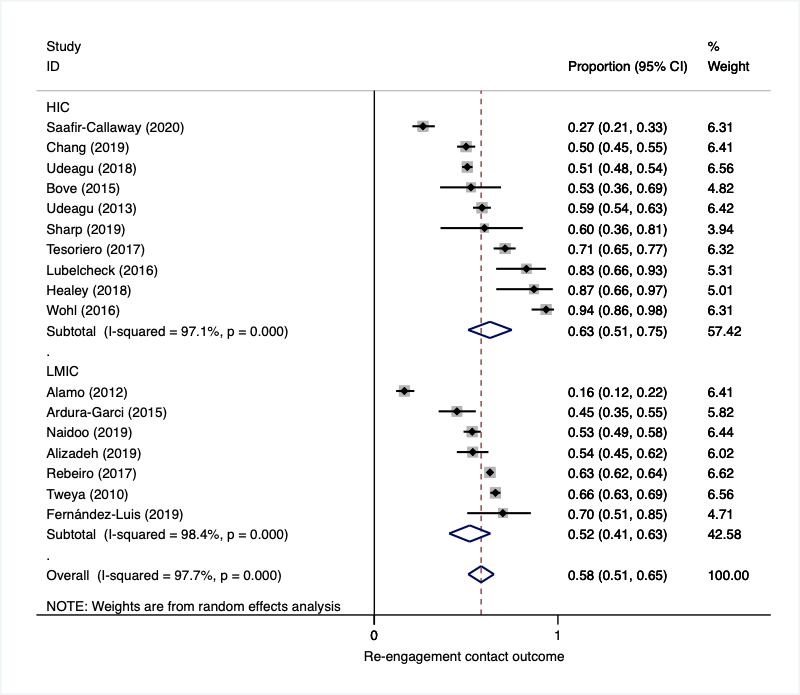


## Supplementary Figure 1: Re-engagement contact outcome: proportion returned to care in intervention arm of comparative and single arm studies, by world bank country income classification


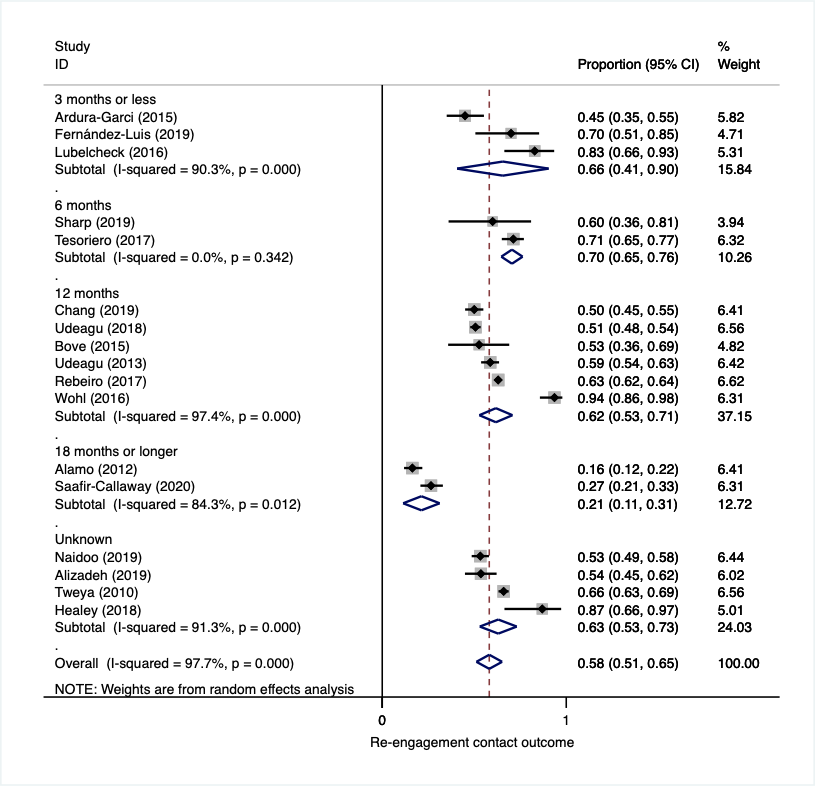


## Supplementary Figure 2: Re-engagement contact outcome: proportion returned to care in intervention arm of comparative and single arm studies, by the time when outcome was measured


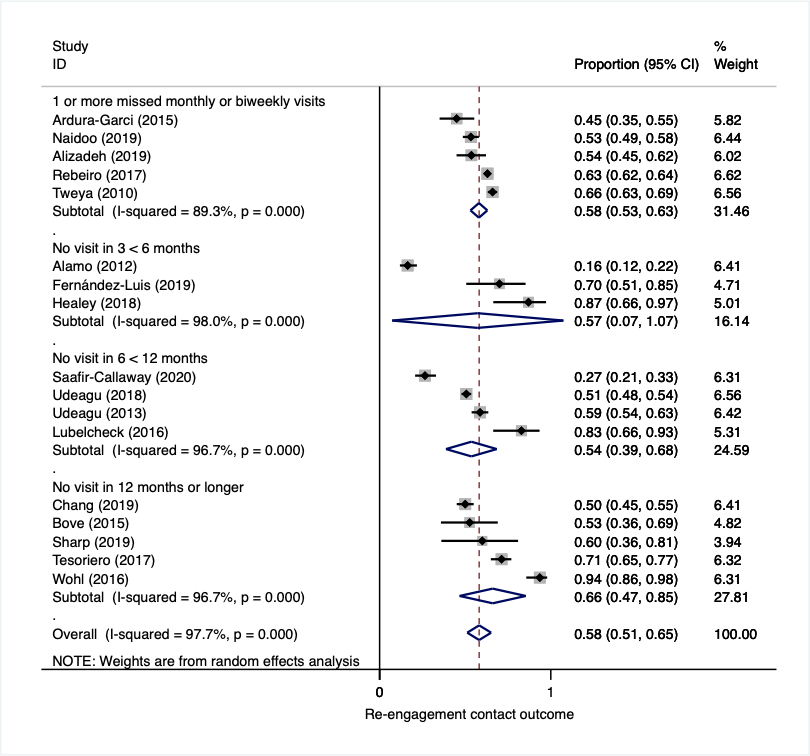


## Supplementary Figure 3: Re-engagement contact outcome: proportion returned to care in intervention arm of comparative and single arm studies, by lost to follow-up definition


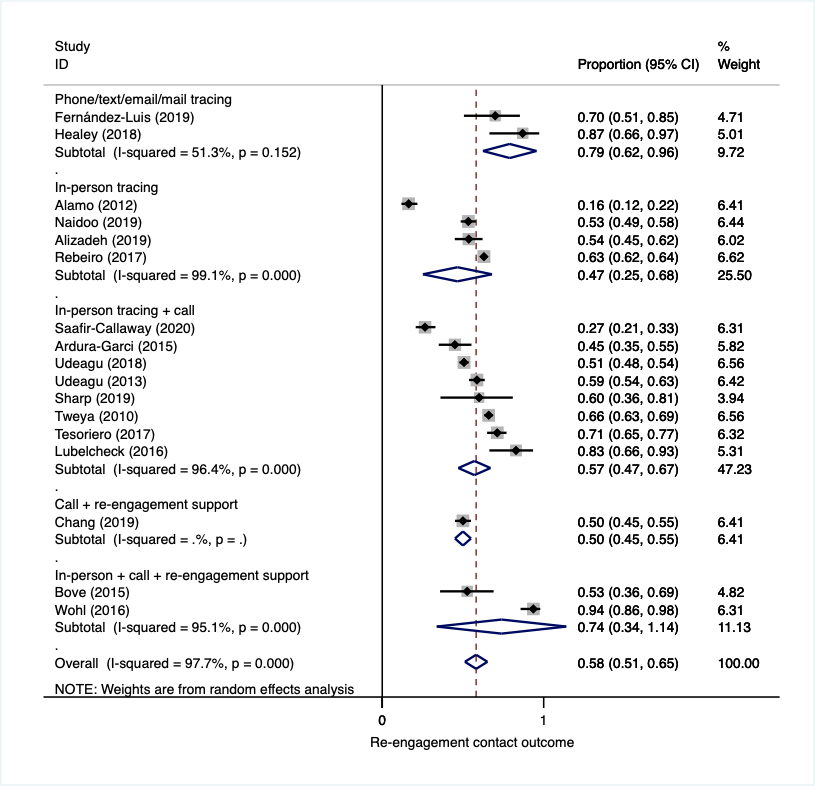


## Supplementary Figure 4: Re-engagement contact outcome: proportion returned to care in intervention arm of comparative and single arm studies, by method of tracing

## Supplementary Table 1: Re-engagement contact outcome: proportion returned to care in intervention arm of comparative and single arm studies, sub-group analyses

| **Subgroups** | **Number of studies** | **Proportion returned (95%CI)*** | **P-value**** | **I-squared***** |
| --- | --- | --- | --- | --- |
| **Overall** | 17 | 0.58 (0.51, 0.65) | 0.001 | 97.70% |
| **Study Design** |  |  |  |  |
| Cohort | 17 | 0.58 (0.51, 0.65) | 0.001 | 97.70% |
| RCT | 0 | ---- | ---- | ---- |
| **Country income** |  |  |  |  |
| High income | 10 | 0.63 (0.51, 0.75) | 0.001 | 97.10% |
| Low-Middle income | 7 | 0.52 (0.41, 0.63) | 0.001 | 98.40% |
| **Tracing type** |  |  |  |  |
| Phone/text/email/mail | 2 | 0.79 (0.62, 0.96) | 0.152 | 51.30% |
| In-person tracing | 4 | 0.47 (0.25, 0.68) | 0.001 | 99.10% |
| In-person tracing + Call | 8 | 0.57 (0.47, 0.67) | 0.001 | 96.40% |
| Call + re-engagement support | 1 | 0.50 (0.45, 0.55) | ---- | ---- |
| In-person tracing + Call + re-engagement support | 2 | 0.74 (0.34, 0.99) | 0.001 | 95.10% |
| **Outcome measured at** |  |  |  |  |
| 3 months or less | 3 | 0.66 (0.41, 0.90) | 0.001 | 90.30% |
| 6 months | 2 | 0.70 (0.65, 0.76) | 0.342 | 0.0% |
| 12 months | 6 | 0.62 (0.53, 0.71) | 0.001 | 97.40% |
| 18 months or longer | 2 | 0.21 (0.11, 0.31) | 0.012 | 84.30% |
| Unknown | 4 | 0.63 (0.53, 0.73) | 0.001 | 91.30% |
| **Definition of lost to follow-up** |  |  |  |  |
| 1 or more missed monthly or biweekly visits | 5 | 0.58 (0.53, 0.63) | 0.001 | 89.30% |
| No visit in 3 < 6 months | 3 | 0.57 (0.07, 0.99) | 0.001 | 98.00% |
| No visit in 6 < 12 months | 4 | 0.54 (0.39, 0.68) | 0.001 | 96.70% |
| No visit in 12 months or longer | 5 | 0.66 (0.47, 0.85) | 0.001 | 96.70% |
| * Random effect model; ** P-value is for Heterogeneity. H0: variation is only by chance; *** the variation in the proportion (outcome) attributable to heterogeneity; RCT: randomized controlled trial | | | | |

## Comparative estimates: Re-engagement program effects (effectiveness)


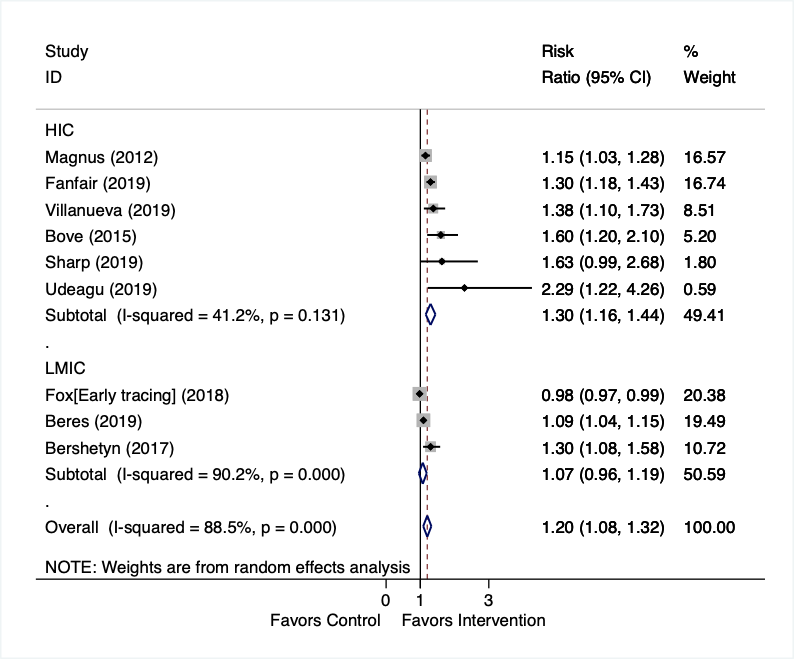


## Supplementary Figure 5: Re-engagement program effects (effectiveness): proportion returned to care in comparative studies, by world bank country income classification


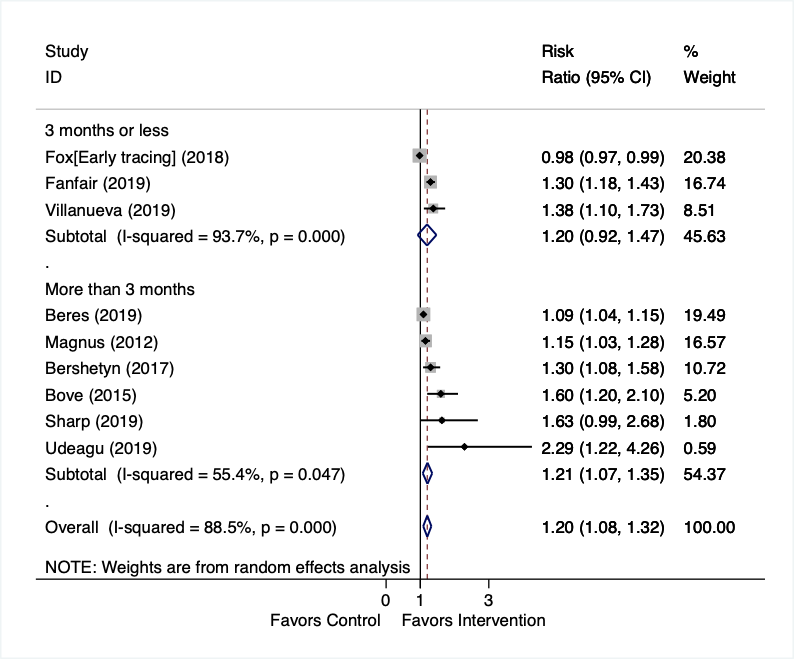


## Supplementary Figure 6: Re-engagement program effects (effectiveness): proportion returned to care in comparative studies, by the time when outcome was measured


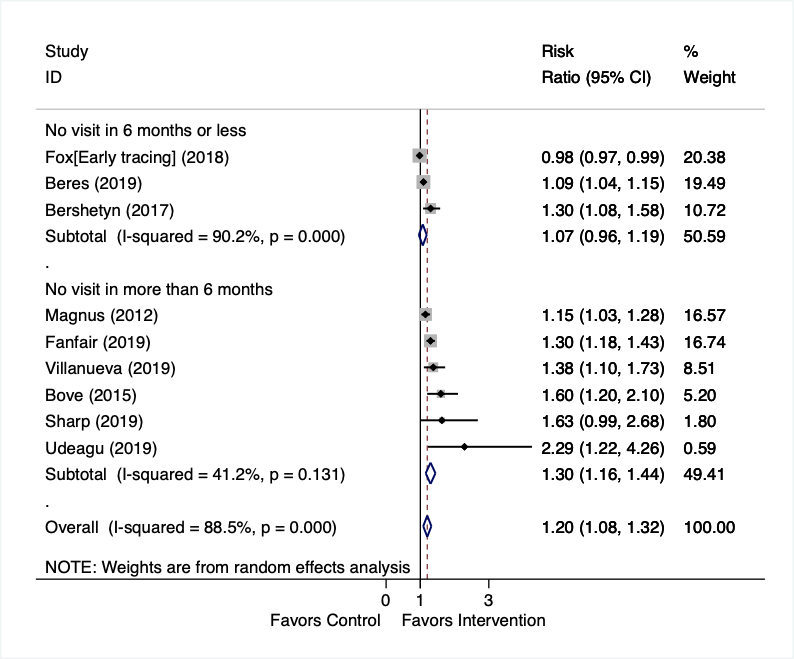


## Supplementary Figure 7: Re-engagement program effects (effectiveness): proportion returned to care in comparative studies, by lost to follow-up definition


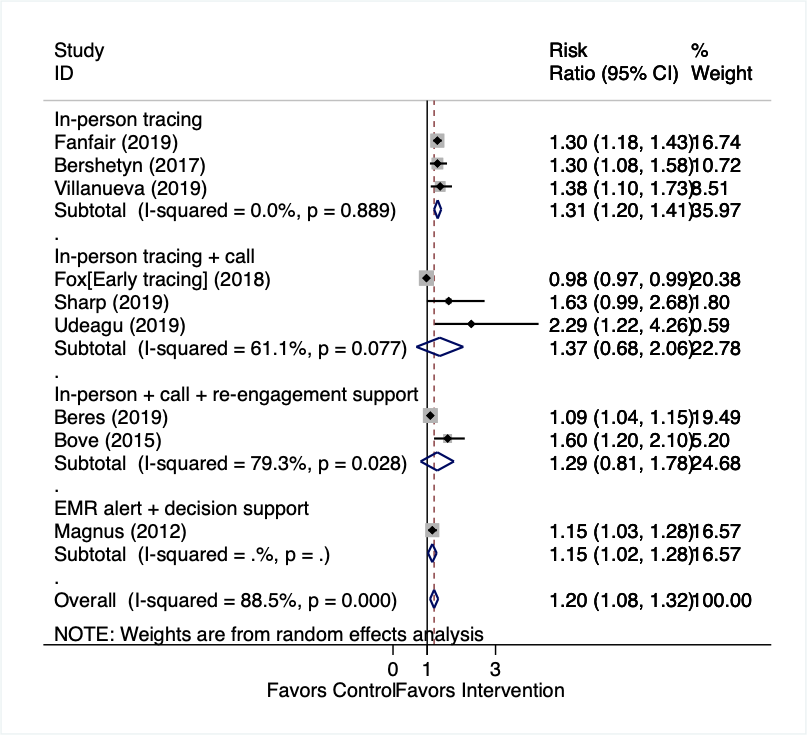


## Supplementary Figure 8: Re-engagement program effects (effectiveness): proportion returned to care in comparative studies, by method of tracing

## Retention in care


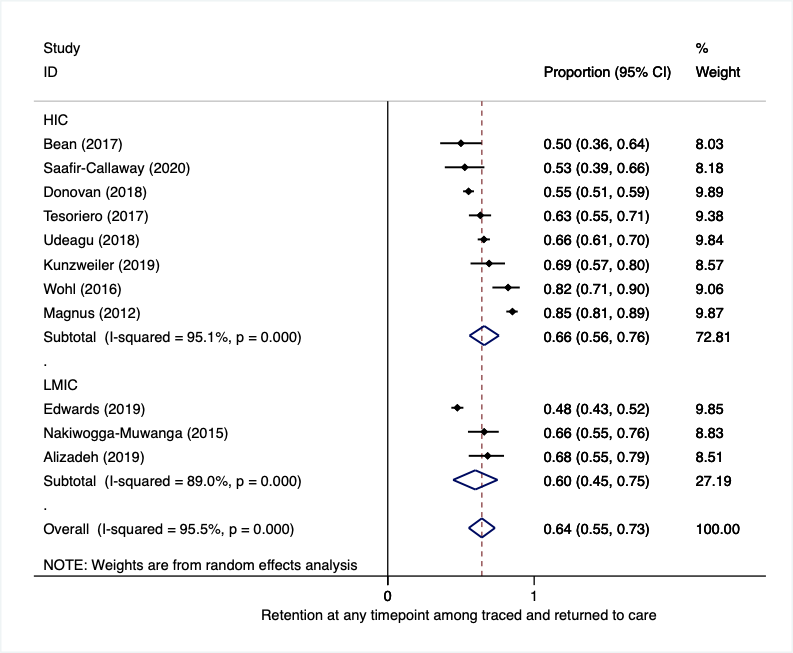


## Supplementary Figure 9: Proportion retained in care after return to care, by world bank country income classification


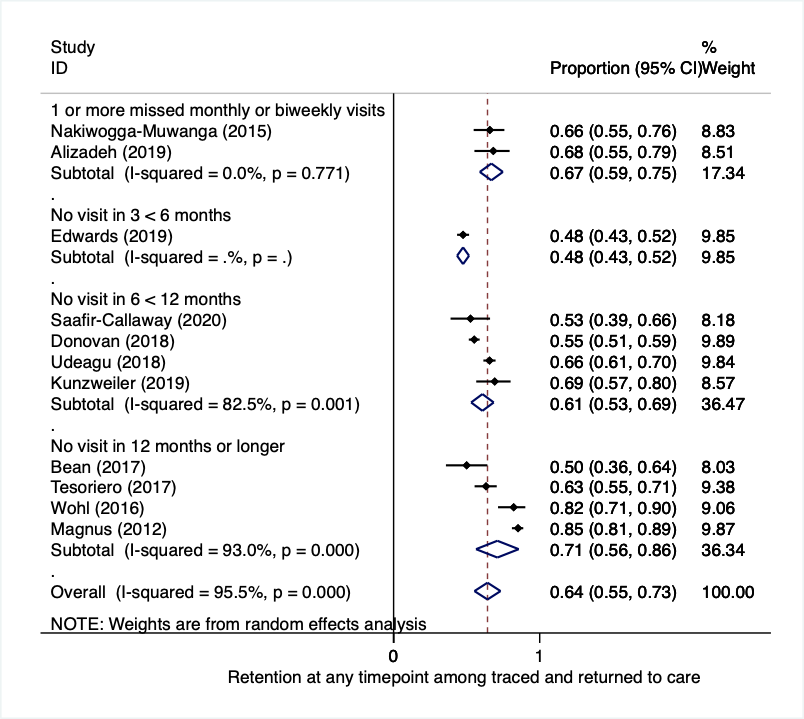


## Supplementary Figure 10: Proportion retained in care after return to care, by lost to follow-up definition


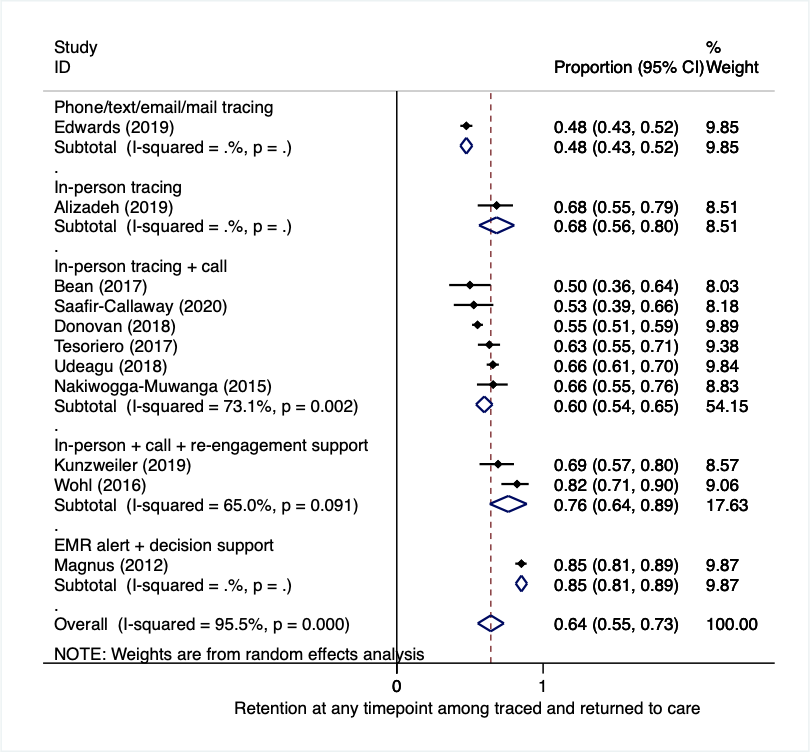


## Supplementary Figure 11: Proportion retained in care after return to care, by tracing method

## Viral suppression


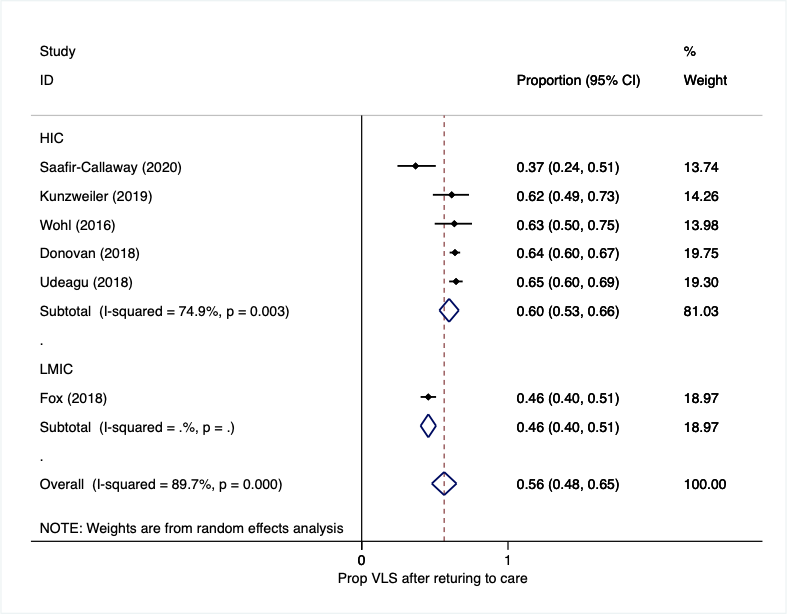


## Supplementary Figure 12: Proportion virally suppressed after return to care, by world bank country income classification


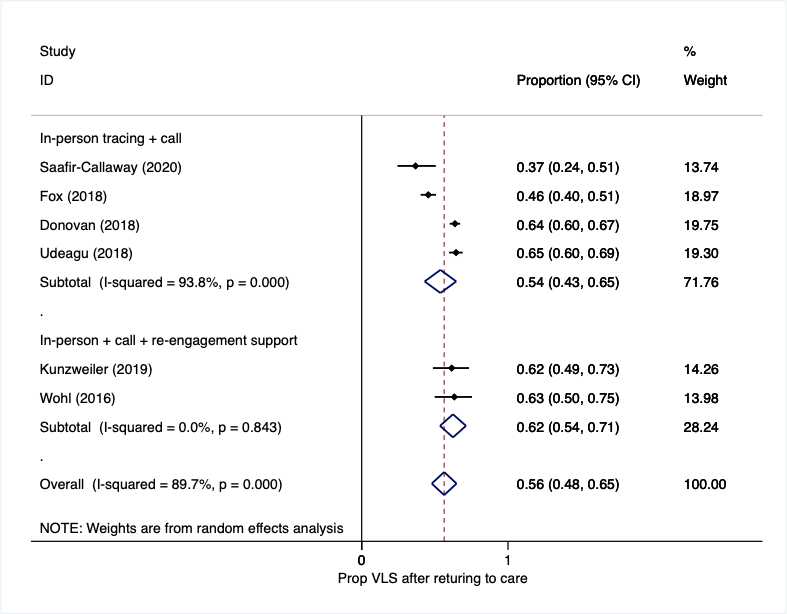


## Supplementary Figure 13: Proportion virally suppressed after return to care, by tracing method


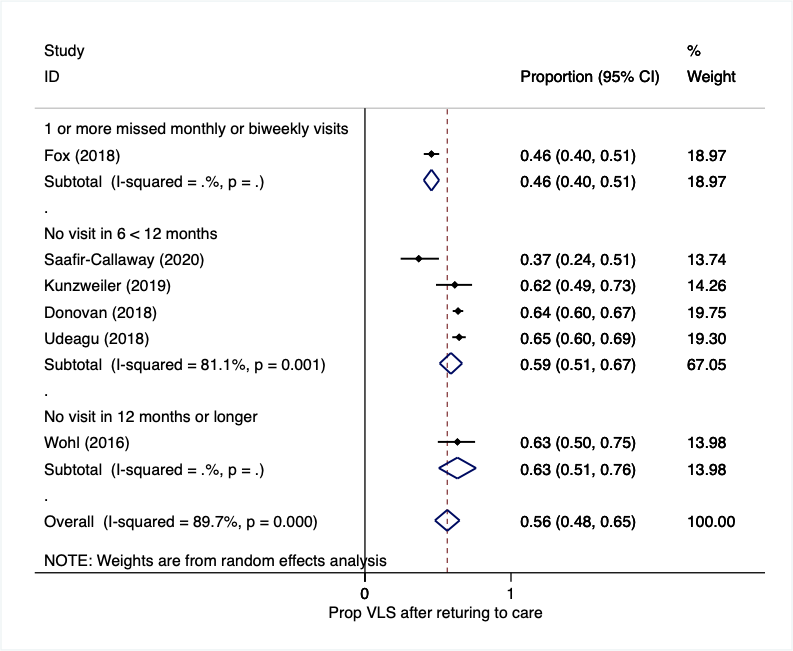


## Supplementary Figure 14: Proportion virally suppressed after return to care, by lost to follow-up definition

## Appendix F – list of included studies

1. Alamo ST, Colebunders R, Ouma J, Sunday P, Wagner G, Wabwire-Mangen F, et al. Return to Normal Life After AIDS as a Reason for Lost to Follow-up in a Community-Based Antiretroviral Treatment Program. JAIDS Journal of Acquired Immune Deficiency Syndromes. 2012;60(2):e36-e45.
2. Amy Rock Wohl M, Rhodri Dierst-Davies, Alla Victoroff, Saloniki James M, Jesse Bendetson, BA, Jeff Bailey, MPH,† Eric Daar, LaShonda Spencer M, Sonali Kulkarni, and Mario J. Pérez. The Navigation Program: An Intervention to Reengage Lost Patients at 7 HIV Clinics in Los Angeles County, 2012–2014. J Acquir Immune Defic Syndr 2016;71:e44–e50. 2016.
3. Ardura-Garcia C, Feldacker C, Tweya H, Chaweza T, Kalulu M, Phiri S, et al. Implementation and Operational Research, JAIDS Journal of Acquired Immune Deficiency Syndromes: December 15, 2015 - Volume 70 - Issue 5 - p e160-e167 2015.
4. Bean MC, Scott L, Kilby JM, Richey LE. Use of an Outreach Coordinator to Reengage and Retain Patients with HIV in Care. AIDS Patient Care STDS. 2017;31(5):222-6.
5. Bershetyn A, Odeny TA, Lyamuya R, Nakiwogga-Muwanga A, Diero L, Bwana M, et al. The Causal Effect of Tracing by Peer Health Workers on Return to Clinic Among Patients Who Were Lost to Follow-up From Antiretroviral Therapy in Eastern Africa: A "Natural Experiment" Arising From Surveillance of Lost Patients. Clin Infect Dis. 2017;64(11):1547-54.
6. Chang EJ, Fleming M, Nunez A, Dombrowski JC. Predictors of Successful HIV Care Re-engagement Among Persons Poorly Engaged in HIV Care. AIDS Behav. 2019;23(9):2490-7.
7. Colin Kunzweiler P, Nina Kishore, MPA, MPH, Betsey John, MPH, Kathleen Roosevelt, MPH,, Sophie Lewis B, R. Monina Klevens, DDS, MPH, Monica Morrison, MPH, Liisa M. Randall, PhD, and, Alfred DeMaria J, MD. Using HIV Surveillance and Clinic Data to Optimize Data to Care Efforts in Community Health Centers in Massachusetts: The Massachusetts Partnerships for Care Project. J Acquir Immune Defic Syndr 2019;82:S33–S41. 2019.
8. Deery CB, Hanrahan CF, Selibas K, Bassett J, Sanne I, Van Rie A. A home tracing program for contacts of people with tuberculosis or HIV and patients lost to care. Int J Tuberc Lung Dis. 2014;18(5):534-40.
9. Donovan J, Sullivan K, Wilkin A, Fadul N, Heine A, Keller J, et al. Past Care Predicts Future Care in Out-of-Care People Living with HIV: Results of a Clinic-Based Retention-in-Care Intervention in North Carolina. AIDS Behav. 2018;22(8):2687-97.
10. E. Chikuse1 KP, P. Kalande1, K. Dovel1,2, M. Sarena1, A. Schooley1,2, EQUIP Innovations for Health. High rates of successful tracing and re-engagement in HIV care using Expert Clients in Malawi. 2018.
11. Faraz Alizadeh, Gideon Mfitumuhoza, Joseph Stephens, Christopher Habimaana, KwiringiraMyles. Michael Baganizi,b Gerald Paccionec, Identifying and Reengaging Patients Lost to Follow-Up in Rural Africa: The “Horizontal” Hospital-Based Approach in Uganda. 2019.
12. Fernandez-Luis S, Fuente-Soro L, Augusto O, Bernardo E, Nhampossa T, Maculuve S, et al. Reengagement of HIV-infected children lost to follow-up after active mobile phone tracing in a rural area of Mozambique. J Trop Pediatr. 2019;65(3):240-8.
13. Fox MP, Pascoe SJS, Huber AN, Murphy J, Phokojoe M, Gorgens M, et al. Effectiveness of interventions for unstable patients on antiretroviral therapy in South Africa: results of a cluster-randomised evaluation. Trop Med Int Health. 2018;23(12):1314-25.
14. Healey L, O'Connor CC. Retaining HIV-positive patients in HIV care: a personalised approach for those at risk of loss to follow-up at an inner city sexual health service. Sex Health. 2018;15(1):91-2.
15. Jeffrey Edwards R, Lyons N, Bhatt C, Samaroo-Francis W, Hinds A, Cyrus E. Implementation and outcomes of a patient tracing programme for HIV in Trinidad and Tobago. Glob Public Health. 2019;14(11):1589-97.
16. Joanna M. Bove, Matthew R. Golden, Shireesha Dhanireddy, Robert D. Harrington, and Julia C. Dombrowski. Outcomes of a Clinic-Based Surveillance-Informed Intervention to Relink Patients to HIV Care. J Acquir Immune Defic Syndr 2015;70:262–268. 2015.
17. Keller J, Heine A, LeViere AF, Donovan J, Wilkin A, Sullivan K, et al. HIV patient retention: the implementation of a North Carolina clinic-based protocol. AIDS Care. 2017;29(5):627-31.
18. L Dufour1 CJ, J Sheppard1, M Marshall1, H Morrin1, G McKinley1, D Chilton2, Bilinska2 aJ. Reducing lost to follow-up (LTFU) rate in a large HIV clinic: a quality improvement project (QIP) to correctly identify those LTFU, improve engagement in care and inform allocation of administrative and clinical resource. 2018.
19. Laura K. Beres1 AM, Kombatende Sikombe4, Lauren Hersch Nicholas3, Ingrid 5 Eshun-Wilson2, Paul Somwe4, Nancy L. Czaicki5Ϯ, Sandra Simbeza4, Jake Pry4, Paul Kaumba4, 6 Sheree Schwartz6, John McGready, Charles B. Holmes1,7, Carolyn Bolton-Moore4, Izukanji 7 Sikazwe4, Julie A. Denison1, Elvin H. Geng2. . The effect of tracer contact on return to care among lost to follow-up adult patients living 1 with HIV in Zambia: An Instrumental Variable Analysis. (Manuscript). 2020.
20. Lubelchek RJ, Fritz ML, Fritz KJ, Trick WE. Use of a Real-Time Alert System to Identify and Re-Engage Lost-to-Care HIV Patients. 2016.
21. M. Bupamba RM, M. Strachan, G. Nkobelerwa, S. Spendi, A. Mkamballah, A. Cunningham,. Ambassadors for adherence": provision of highly effective defaulter tracing and re-engagement by peer educators in Tanzania. 2010.
22. Magnus M, Herwehe J, Gruber D, Wilbright W, Shepard E, Abrams A, et al. Improved HIV-related outcomes associated with implementation of a novel public health information exchange. Int J Med Inform. 2012;81(10):e30-8.
23. McMahon JH, Moore R, Eu B, Tee BK, Chen M, El-Hayek C, et al. Clinic Network Collaboration and Patient Tracing to Maximize Retention in HIV Care. PLoS One. 2015;10(5):e0127726.
24. Merceditas Villanueva1 CC, Janet Miceli1, Suzanne Speers2, Lisa Nichols1 , Frederick Altice1 and Heidi Jenkins2. Project CoRECT: Preliminary Results of Data to Care With CT DPH and HIV Clinics. 2019.
25. Nabaggala MS, Parkes-Ratanshi R, Kasirye R, Kiragga A, Castlenuovo B, Ochaka I, et al. Re-engagement in HIV care following a missed visit in rural Uganda. BMC Res Notes. 2018;11(1):762.
26. Naidoo N, Matlakala N, Railton J, Khosa S, Marincowitz G, Igumbor JO, et al. Provision of HIV services by community health workers should be strengthened to achieve full programme potential: a cross-sectional analysis in rural South Africa. Trop Med Int Health. 2019;24(4):401-8.
27. Nakiwogga-Muwanga A, Musaazi J, Katabira E, Worodria W, Talisuna SA, Colebunders R. Patients who return to care after tracking remain at high risk of attrition: experience from a large HIV clinic, Uganda. Int J STD AIDS. 2015;26(1):42-7.
28. Peter F. Rebeiro GB, Beverly S. Musick, Ronald S. Braithwaite, Kara K. Wools-Kaloustian, Winstone Nyandiko, Fatma Some,, Paula Braitstein, Constantin T. Yiannoutsos. Observational Study of the Effect of Patient Outreach on Return to Care: The Earlier the Better. 2017.
29. Robyn Neblett Fanfair1 GK, Nasima Camp1,6 Kathleen Brady2, Alfred DeMaria3, Merceditas Villanueva4, Liisa Randall3, Heidi Jenkins5, Crystal Lucas2, Frederick Altice4, Anthony Gerard2, Nina Kishore3, Tiffany Williams1,6 Taraz Samandari1, Paul J Weidle. Health Department Randomized Trial to Re-engage Out-of-Care HIV Infected Persons - The Cooperative Re-Engagement Controlled Trial. 2019.
30. Saafir-Callaway B, Castel AD, Lago L, Olejemeh C, Lum G, Frison L, et al. Longitudinal outcomes of HIV- infected persons re-engaged in care using a community-based re-engagement approach. AIDS Care. 2020;32(1):76-82.
31. Sharp J, Angert CD, McConnell T, Wortley P, Pennisi E, Roland L, et al. Health Information Exchange: A Novel Re-linkage Intervention in an Urban Health System. Open Forum Infect Dis. 2019;6(10):ofz402.
32. Sitapati AM, Limneos J, Bonet-Vazquez M, Mar-Tang M, Qin H, Mathews WC. Retention: building a patient-centered medical home in HIV primary care through PUFF (Patients Unable to Follow-up Found). J Health Care Poor Underserved. 2012;23(3 Suppl):81-95.
33. Tesoriero JM, Johnson BL, Hart-Malloy R, Cukrovany JL, Moncur BL, Bogucki KM, et al. Improving Retention in HIV Care Through New York's Expanded Partner Services Data-to-Care Pilot. J Public Health Manag Pract. 2017;23(3):255-63.
34. Tweya H, Gareta D, Chagwera F, Ben-Smith A, Mwenyemasi J, Chiputula F, et al. Early active follow-up of patients on antiretroviral therapy (ART) who are lost to follow-up: the 'Back-to-Care' project in Lilongwe, Malawi. Trop Med Int Health. 2010;15 Suppl 1:82-9.
35. Udeagu C, Huang J, Eason L, Pickett L. Health department-HIV clinic integration of data and human resources to re-engage out of care HIV-positive persons into clinical care in a New York City locale. AIDS Care. 2019;31(11):1420-6.
36. Udeagu CC, Webster TR, Bocour A, Michel P, Shepard CW. Lost or just not following up: public health effort to re-engage HIV-infected persons lost to follow-up into HIV medical care. AIDS. 2013;27(14):2271-9.
37. Udeagu CN, Shah S, Misra K, Sepkowitz KA, Braunstein SL. Where Are They Now? Assessing if Persons Returned to HIV Care Following Loss to Follow-Up by Public Health Case Workers Were Engaged in Care in Follow-Up Years. AIDS Patient Care STDS. 2018;32(5):181-90.

## Appendix G – PRISMA

| **Section and Topic** | **Item #** | **Checklist item** | **Location where item is reported** |
| --- | --- | --- | --- |
| **TITLE** | | |  |
| Title | 1 | Identify the report as a systematic review. | Title |
| **ABSTRACT** | | |  |
| Abstract | 2 | See the PRISMA 2020 for Abstracts checklist. | Abstract |
| **INTRODUCTION** | | |  |
| Rationale | 3 | Describe the rationale for the review in the context of existing knowledge. | Intro – 2^nd^ paragraph |
| Objectives | 4 | Provide an explicit statement of the objective(s) or question(s) the review addresses. | Intro – last paragraph |
| **METHODS** | | |  |
| Eligibility criteria | 5 | Specify the inclusion and exclusion criteria for the review and how studies were grouped for the syntheses. | Methods – 2^nd^ paragraph |
| Information sources | 6 | Specify all databases, registers, websites, organisations, reference lists and other sources searched or consulted to identify studies. Specify the date when each source was last searched or consulted. | Methods – Search strategy section |
| Search strategy | 7 | Present the full search strategies for all databases, registers and websites, including any filters and limits used. | Methods – Search strategy section |
| Selection process | 8 | Specify the methods used to decide whether a study met the inclusion criteria of the review, including how many reviewers screened each record and each report retrieved, whether they worked independently, and if applicable, details of automation tools used in the process. | Methods - Screening and data extraction section |
| Data collection process | 9 | Specify the methods used to collect data from reports, including how many reviewers collected data from each report, whether they worked independently, any processes for obtaining or confirming data from study investigators, and if applicable, details of automation tools used in the process. | Methods - Screening and data extraction section |
| Data items | 10a | List and define all outcomes for which data were sought. Specify whether all results that were compatible with each outcome domain in each study were sought (e.g. for all measures, time points, analyses), and if not, the methods used to decide which results to collect. | Methods - Analysis section |
|  | 10b | List and define all other variables for which data were sought (e.g. participant and intervention characteristics, funding sources). Describe any assumptions made about any missing or unclear information. | Methods - Analysis section |
| Study risk of bias assessment | 11 | Specify the methods used to assess risk of bias in the included studies, including details of the tool(s) used, how many reviewers assessed each study and whether they worked independently, and if applicable, details of automation tools used in the process. | Methods - Assessments of methodological quality section |
| Effect measures | 12 | Specify for each outcome the effect measure(s) (e.g. risk ratio, mean difference) used in the synthesis or presentation of results. | Methods - Analysis section |
| Synthesis methods | 13a | Describe the processes used to decide which studies were eligible for each synthesis (e.g. tabulating the study intervention characteristics and comparing against the planned groups for each synthesis (item #5)). | Methods - Analysis section |
|  | 13b | Describe any methods required to prepare the data for presentation or synthesis, such as handling of missing summary statistics, or data conversions. | Methods - Analysis section |
|  | 13c | Describe any methods used to tabulate or visually display results of individual studies and syntheses. | Methods - Analysis section |
|  | 13d | Describe any methods used to synthesize results and provide a rationale for the choice(s). If meta-analysis was performed, describe the model(s), method(s) to identify the presence and extent of statistical heterogeneity, and software package(s) used. | Methods - Analysis section |
|  | 13e | Describe any methods used to explore possible causes of heterogeneity among study results (e.g. subgroup analysis, meta-regression). | Methods - Analysis section |
|  | 13f | Describe any sensitivity analyses conducted to assess robustness of the synthesized results. | NA |
| Reporting bias assessment | 14 | Describe any methods used to assess risk of bias due to missing results in a synthesis (arising from reporting biases). | Methods - Assessments of methodological quality section |
| Certainty assessment | 15 | Describe any methods used to assess certainty (or confidence) in the body of evidence for an outcome. | Methods - Assessments of methodological quality section |
| **RESULTS** | | |  |
| Study selection | 16a | Describe the results of the search and selection process, from the number of records identified in the search to the number of studies included in the review, ideally using a flow diagram. | Results - Characteristics of included studies section |
|  | 16b | Cite studies that might appear to meet the inclusion criteria, but which were excluded, and explain why they were excluded. | Characteristics of included studies and Figure 2 |
| Study characteristics | 17 | Cite each included study and present its characteristics. | Results + Table 1 |
| Risk of bias in studies | 18 | Present assessments of risk of bias for each included study. | Results + Table 3 & 4 |
| Results of individual studies | 19 | For all outcomes, present, for each study: (a) summary statistics for each group (where appropriate) and (b) an effect estimate and its precision (e.g. confidence/credible interval), ideally using structured tables or plots. | Results – Figure 4 |
| Results of syntheses | 20a | For each synthesis, briefly summarise the characteristics and risk of bias among contributing studies. | Results – Table 6 |
|  | 20b | Present results of all statistical syntheses conducted. If meta-analysis was done, present for each the summary estimate and its precision (e.g. confidence/credible interval) and measures of statistical heterogeneity. If comparing groups, describe the direction of the effect. | Results – Figure 4 + Table 6 |
|  | 20c | Present results of all investigations of possible causes of heterogeneity among study results. | Results – Table 6 |
|  | 20d | Present results of all sensitivity analyses conducted to assess the robustness of the synthesized results. | NA |
| Reporting biases | 21 | Present assessments of risk of bias due to missing results (arising from reporting biases) for each synthesis assessed. | Results – Table 5 |
| Certainty of evidence | 22 | Present assessments of certainty (or confidence) in the body of evidence for each outcome assessed. | Results - GRADE Evidence certainty |
| **DISCUSSION** | | |  |
| Discussion | 23a | Provide a general interpretation of the results in the context of other evidence. | Discussion - 1^st^ Paragraph |
|  | 23b | Discuss any limitations of the evidence included in the review. | Discussion – 2^nd^ and 3^rd^ paragraphs |
|  | 23c | Discuss any limitations of the review processes used. | Discussion – Limitations |
|  | 23d | Discuss implications of the results for practice, policy, and future research. | Discussion – Conclusion |
| **OTHER INFORMATION** | | |  |
| Registration and protocol | 24a | Provide registration information for the review, including register name and registration number, or state that the review was not registered. | Methods – 1^st^ paragraph |
|  | 24b | Indicate where the review protocol can be accessed, or state that a protocol was not prepared. | Methods – 1^st^ paragraph |
|  | 24c | Describe and explain any amendments to information provided at registration or in the protocol. | NA |
| Support | 25 | Describe sources of financial or non-financial support for the review, and the role of the funders or sponsors in the review. | Journal Metadata |
| Competing interests | 26 | Declare any competing interests of review authors. | Journal Metadata |
| Availability of data, code and other materials | 27 | Report which of the following are publicly available and where they can be found: template data collection forms; data extracted from included studies; data used for all analyses; analytic code; any other materials used in the review. | NA |

## Appendix H – Publication Bias

| **Re-engagement contact outcome**    Regression-based Egger test for small-study effects: P = 0.319  There is no indication for publication bias and  small-study effects. | **Re-engagement program outcome**    Regression-based Egger test for small-study effects: P = 0.149  There is no indication for publication bias and  small-study effects. |
| --- | --- |
| **Re-engagement program effects** (proportion LTFU returned to original clinic), re-engagement program versus no intervention or SOC)    Regression-based Egger test for small-study effects: P = 0.006  There is an indication for publication bias and  small-study effects. | **Proportion virally suppressed after return to care**    Regression-based Egger test for small-study effects: P = 0.556  There is no indication for publication bias and  small-study effects. |
| **Retention at any timepoint among traced and returned to care**    Regression-based Egger test for small-study effects: P = 0.739  There is no indication for publication bias and  small-study effects. | **Proportion retained in care after return to care**    Regression-based Egger test for small-study effects: P = 0.319  There is no indication for publication bias and  small-study effects. |
| **Re-engagement contact effects**: proportion returned to care among patients who were contacted and found out of care in comparative studies  Only two studies reported this outcome, and so, we did not assess the publication bias and small-study effects. |  |
